# A Role for Barley Calcium-Dependent Protein Kinase CPK2a in the Response to Drought

**DOI:** 10.3389/fpls.2016.01550

**Published:** 2016-10-25

**Authors:** Agata Cieśla, Filip Mituła, Lucyna Misztal, Olga Fedorowicz-Strońska, Sabina Janicka, Małgorzata Tajdel-Zielińska, Małgorzata Marczak, Maciej Janicki, Agnieszka Ludwików, Jan Sadowski

**Affiliations:** ^1^Biotechnology Department, Faculty of Biology, Adam Mickiewicz UniversityPoznań, Poland; ^2^Institute of Plant Genetics, Polish Academy of SciencePoznań, Poland

**Keywords:** calcium-dependent protein kinase, *Hordeum vulgare* L., drought stress, mass spectrometry, protein function

## Abstract

Increasing the drought tolerance of crops is one of the most challenging goals in plant breeding. To improve crop productivity during periods of water deficit, it is essential to understand the complex regulatory pathways that adapt plant metabolism to environmental conditions. Among various plant hormones and second messengers, calcium ions are known to be involved in drought stress perception and signaling. Plants have developed specific calcium-dependent protein kinases that convert calcium signals into phosphorylation events. In this study we attempted to elucidate the role of a calcium-dependent protein kinase in the drought stress response of barley (*Hordeum vulgare* L.), one of the most economically important crops worldwide. The ongoing barley genome project has provided useful information about genes potentially involved in the drought stress response, but information on the role of calcium-dependent kinases is still limited. We found that the gene encoding the calcium-dependent protein kinase *HvCPK2a* was significantly upregulated in response to drought. To better understand the role of HvCPK2a in drought stress signaling, we generated transgenic Arabidopsis plants that overexpressed the corresponding coding sequence. Overexpressing lines displayed drought sensitivity, reduced nitrogen balance index (NBI), an increase in total chlorophyll content and decreased relative water content. In addition, *in vitro* kinase assay experiments combined with mass spectrometry allowed HvCPK2a autophosphorylation sites to be identified. Our results suggest that HvCPK2a is a dual-specificity calcium-dependent protein kinase that functions as a negative regulator of the drought stress response in barley.

## Introduction

Drought stress is one of the most critical threats to world agriculture. The severity and duration of drought are largely unpredictable, but even short periods of water deficit reduce plant growth and crop productivity. The ensuing reduced crop yields affect certain regional economies by limiting their ability to compete in agricultural markets. Consequently, the improvement of yields under drought conditions is now a major goal of plant breeding programs worldwide. Because the effect of drought on crop yield is determined by multiple factors, understanding the plant response to dry conditions is fundamental to the generation of drought-tolerant cultivars (Zhu, 2002; Rampino et al., 2006).

Plant responses and adaptation to drought are achieved at the molecular, biochemical, and physiological levels (Morari et al., 2015). Abscisic acid (ABA) is recognized as the most critical hormone in plant responses to water deficit. In addition, many functionally diverse second messengers are involved in drought stress tolerance (Zeng et al., 2015), including calcium and calcium sensors, which are important for signaling and subsequent adaptation to stress conditions (Klimecka and Muszynska, 2007; Kudla et al., 2010; Reddy et al., 2011; Vivek et al., 2013). Plants have evolved several classes of calcium-binding proteins, including calcineurin B-like (CBL), calmodulin (CaM) and calmodulin-related proteins (Cheng et al., 2002; Luan et al., 2002). Calcium-dependent protein kinases (CPKs), which convert calcium signals into phosphorylation events, have been identified as key intracellular protein kinases in the drought stress response. Calcium-binding proteins also play a crucial role in numerous other physiological processes, including hormone signaling and pathogen responses (Sheen, 1996; Romeis et al., 2001; Guo et al., 2002; Fan et al., 2004; Ludwig et al., 2004; Boudsocq and Sheen, 2013).

CPKs are highly conserved proteins involved in plant growth, development, stress signaling, defense responses and proteasome regulation (Sheen, 1996; Romeis et al., 2001; Harper et al., 2004; Boudsocq et al., 2010; Asano et al., 2012; Hubbard et al., 2012). They are monomeric proteins consisting of five domains: a kinase domain, an autoinhibitory domain, a regulatory domain, a variable domain located at the N-terminus, and a short C-terminal domain. CPKs are regulated by Ca^2+^ ions that bind to four EF-hand motifs with distinct calcium affinities (Harmon et al., 2000; Klimecka and Muszynska, 2007; Boudsocq and Sheen, 2013). In addition to activation by calcium binding, CPKs are also regulated by phospholipids, reversible phosphorylation events and adapter proteins (Romeis et al., 2000; Chehab et al., 2004; Harper et al., 2004; Hegeman et al., 2006; Klimecka and Muszynska, 2007; Witte et al., 2010; Oh et al., 2012). Although some CPKs are cytosolic, the great majority of CPKs identified have potential N-myristoylation and palmitylation motifs for membrane association in the N-terminal domain (Harper et al., 2004; Klimecka and Muszynska, 2007; Benetka et al., 2008; Martin et al., 2012; Boudsocq and Sheen, 2013).

Growing evidence indicates that CPKs positively or negatively regulate drought stress responses. Thus, the *Arabidopsis thaliana* CPK, AtCPK6, which controls accumulation of the compatible osmolyte proline (Xu et al., 2010), is a positive regulator of the drought stress response. Other examples of CPKs with a positive regulatory effect include AtCPK10 and OsCPK13 (first named as CDPK7), which are necessary for enhanced drought tolerance in *A. thaliana* and rice (*Oryza sativa*), respectively (Saijo et al., 2000, 2001; Zou et al., 2010). In contrast, AtCPK23, which controls the plasma membrane-bound slow-voltage gated ion channels slow anion channel-associated (SLAC1) and SLAC1 homolog 3 (SLAH3) (Geiger et al., 2010, 2011), negatively regulates drought and salt resistance (Ma and Wu, 2007).

Recently, members of the CPK family were also identified as ABA signaling components that regulate plant responses to abiotic stresses (Ma and Wu, 2007; Zhu et al., 2007; Zhao et al., 2011a,b). For example, AtCPK4, AtCPK11, AtCPK10, AtCPK30, and AtCPK32 all mediate ABA responses by phosphorylating ABA-responsive transcription factors (ABFs), leading to modulation of gene expression (Choi et al., 2005; Zhu et al., 2007. AtCPK12 is involved in ABA signaling in seeds and young seedlings (Zhao et al., 2011b), while AtCPK3, AtCPK6, and AtCPK23 have been identified as players in ABA-regulated stomatal signaling (Mori et al., 2006; Ma and Wu, 2007). Certain AtCPKs, including CPK4, CPK11, and CPK23, which regulate calcium-mediated ABA signaling pathways, are also involved in tolerance to drought and salt stresses (Ma and Wu, 2007; Zhu et al., 2007; Zhao et al., 2011a,b). In addition, 10 out of 14 wheat *CPK* genes appeared to respond to abiotic stresses including drought and salt stress, as well as ABA stimulus (Li et al., 2008). Five CPK family members in wheat (TaCPK1, TaCPK6, TaCPK7, TaCPK9, and TaCPK18) were implicated in abiotic stress response including drought, osmotic stress, salt and temperature (Li et al., 2008; Geng et al., 2011).

Barley (*Hordeum vulgare* L.) is the fourth most significant cereal crop plant after wheat, maize and rice (FAO). The recent availability of the barley genome sequence, together with various classical and large-scale approaches, facilitate the identification of candidate genes involved in drought-stress adaptation (Sreenivasulu et al., 2008; Mochida and Shinozaki, 2013). Despite the importance of a variety of calcium sensors in the response to drought, CPK-dependent signal transduction in barley has not yet been characterized. Here, we investigate the role of HvCPK2a in the barley drought response.

## Methods

### Plant growth and treatments

For liquid chromatography-tandem mass spectrometry (LC-MS/MS) and protoplast isolation, barley cultivar *H. vulgare* L. cv. “Sebastian” was sown in pots filled with a mixture of peat and sand (2:7) with high content of phosphorus and potassium, a standard content of magnesium and a low sulfur content. Pots with 12 barley plants (28 pots for each experimental variant) were grown in a growth room at 21°C/15°C (day/night) with a 16-h photoperiod (photon flux density 800 μmol m^−2^ s^−1^) and 50% air humidity. The soil water content was maintained at 65%. Plants were grown for ~6 weeks (33 growth stage according Zadocs scale). Drought stress was imposed by withholding water, while in control plants the watering regime was unchanged. After 3 days, the soil water content in drought-exposed plants decreased to 35–45%. *A. thaliana* Col-0 was grown and stably transformed using the floral dip method as described in Ludwików et al. (2009). For the survival test, 3-week old plants that overexpressed *HvCPK2a* were subjected to drought stress. After 10 days, plants were rehydrated and assessed after a 4-day recovery period. Plants were scored as survivors if the base of the shoots remained green after the recovery period. Leaf relative water content was calculated according to the following formula: RWC = (fresh weight – dry weight)/(turgid weight – dry weight) × 100.

### Sequence alignment and phylogenetic analysis

Multiple alignments of Arabidopsis, rice and 23 barley CPK amino acid sequences were performed using Clustal W (Thompson et al., 1997). Unrooted phylogenetic trees were constructed with the MEGA6 program (Tamura et al., 2013) using the neighbor joining method. Amino acid sequences were obtained from NCBI. Sequence identity and sequence similarity were calculated using the SIAS tool at http://imed.med.ucm.es/Tools/sias.html, where sequence identity is the number of exactly matching residues (expressed as a percentage) in a sequence alignment between two sequences of the alignment. Percent similarity is used to quantify the amino acid similarity between two sequences of the alignment.

### Immune complex kinase assay

To identify barley calcium protein kinases (CPKs) involved in the drought stress response, total protein was isolated from drought-stressed barley plants using protein isolation buffer (20 mM Hepes pH 7.5, 10 mM MgCl_2_, 1 mM DTT, 60 mM β-glycerophosphate, 1 mM NaF, 1 mM phenylmethylsulfonyl fluoride, 0.1 mM NaVO_3_, Roche inhibitors). Cell extracts containing 2 mg total protein were immunoprecipitated overnight at 4°C with 25 μl serum of anti-CPK antibodies AS1 or AS2 (AS13 2754, AS16 3836, respectively; Agrisera); the antibodies were pre-coupled to Dynabeads for 1 h at room temperature in 1x PBS. Alternatively, 2–5 mg cell extracts were centrifuged at 10,000 × g for 20 min at 4°C. The resulting soluble (supernatant) and total microsomal fractions (pellet resuspended in 1 ml isolation buffer) were subjected to immunoprecipitation as indicated above. The immunoprecipitated proteins were washed three times with wash buffer I (20 mM Tris-HCl pH 7.5, 5 mM EDTA, 100 mM NaCl, 1% Triton X-100), once with the same buffer but containing 1 M NaCl, then finally washed with kinase buffer (20 mM Hepes pH 7.5, 10 mM MgCl_2_, 1 mM DTT, 1 mM CaCl_2_) and analyzed by *in vitro* kinase assays at 30°C in kinase buffer containing 25 μM ATP, [γ-^32^P]ATP (2 μCi per reaction) with or without 2 mM EGTA and myelin basic protein (MBP) as a substrate. The reactions were stopped by the addition of SDS-loading buffer after 60 min. SDS-PAGE reaction products were analyzed by autoradiography. For LS-MS/MS analysis, immune complexes were resuspended in Tris pH 7.0 and 150 mM NaCl and analyzed.

### Preparation of soluble and membrane protein fractions

For membrane fractionation experiments, 5 g tobacco leaves expressing HvCPK2a-GFP-His protein were homogenized with mortar and pestle in homogenization buffer [1xPBS pH 7.4, 0.1 M NaCl, 0.1% NP-40, 1 mM DTT, 1 mM PMSF a cocktail of protease inhibitors (Roche)]. After filtration through two layers of Miracloth (Millipore), the homogenate was centrifuged at 10,000 × g for 20 min at 4°C to remove intact organelles and cell walls. The resulting supernatant was used as a soluble protein fraction. The membrane pellet was resuspended in homogenizing buffer and sonicated (15 cycles, each of 30 s power on/30 s off). The HvCPK2a-His-GFP protein was then purified from the microsomal fraction using Ni-NTA affinity chromatography and analyzed by immunoblot or by LC-MS/MS in the Laboratory of Mass Spectrometry, Institute of Biochemistry and Biophysics, Polish Academy of Sciences (Warsaw, Poland).

### LC-MS/MS analysis and data processing

For protein identification, independent samples were concentrated and desalted on a RP-C18 pre-column (Waters). Peptide separation was achieved on a nano-Ultra Performance Liquid Chromatography (UPLC) RP-C18 column (Waters, BEH130 C18 column, 75 μm i.d., 250 mm long) of a nanoACQUITY UPLC system using a 45 min linear acetonitrile gradient. Peptides were subjected to electrospray ionization (ESI) using the ion source of an Orbitrap Velos mass spectrometer (Thermo). Higher-energy collisional dissociation (HCD) was used to obtain ion fragments. An electrospray voltage of 1.5 kV was used. Raw data files were pre-processed with Mascot Distiller software (version 2.4.2.0, MatrixScience). The peptide masses and fragmentation spectra obtained were matched against the *A. thaliana* database TAIR10 (70772 sequences/28965710 residues) using the probability based protein identification algorithm Mascot (Mascot Daemon v. 2.4.0, Mascot Server v. 2.4.1, Matrix Science). Search parameters were set as follows: enzyme specificity: semi trypsin; peptide mass tolerance: ±30 ppm; fragment mass tolerance: ±0.1 Da; fixed modifications: carbamidomethylation of cysteine; variable modifications: oxidation of methionine, phosphorylation of serine, threonine, tyrosine and ubiquitination of lysine. The protein mass was left as unrestricted, and mass values as monoisotopic with two missed cleavages allowed. To avoid false positive results, a decoy search was performed. The expected value threshold used for analysis was 0.05, which means that all peptide identifications had less than 1 in 20 chance of being a random match. Unique proteins identified in at least three or more replicates were regarded as putative HvCPK2a protein complex elements. The mass spectrometry proteomics data have been deposited to the ProteomeXchange Consortium via the PRIDE partner repository with the dataset identifier PXD005095 (Vizcaíno et al., 2014, 2016). To identify HvCPK2a phosphorylation sites, tryptic peptides were enriched in phosphopeptides using a magnetic Ti-IMAC method according to the manufacturer's procedure (MagReSyn, ReSyn Biosciences). The spectra of peptides reported as being phosphorylated were examined manually to confirm the precise sites of phosphorylation.

### qPCR analysis

The third leaves from 10 barley plants were ground into fine powder in liquid nitrogen and total RNA was extracted using TRIZOL reagent according to the manufacturer's manual (Life Technologies). The isolated RNA was purified with SV Total RNA Isolation System (Promega) and cDNA synthesis was performed in duplicate using a SuperScript cDNA Synthesis Kit (Invitrogen). Quantitative RT-PCR (qPCR) analysis was performed using the Stratagene Mx3000P Cycler system with Brilliant III Ultra-Fast SYBR QPCR MM Supermix (Stratagene) in a total volume of 20 μl. The reactions were performed as technical duplicates using independent cDNA synthesis reactions. For each condition three biological replicates were conducted. Expression values were normalized against ADP-ribosylation factor cDNA, which was amplified using the primers 5′-TGCTGAATGAGGATGAGCTG-3′ and 5′-GTCCCTCGTACAACCCTTCA-3′. Gene-specific primers used for amplification of *HvCPK2a* transcripts were 5′-GGGCAAGCTACACAAAGGAG-3′ and 5′-ACGTAGCCGTGGAGGTTGTA-3′.

### Plasmid construction

To prepare *HvCPK2a* constructs, cDNA clone 3093 NII, which encodes the barley kinase, was obtained from the National Institute of Agrobiological Sciences, Japan. *HvCPK2a* cDNA was amplified by *Pfu* DNA polymerase using the primers For 5′-CACCATGGGAAACTGCTGCGC-3′ and either N-Rev 5′-TAGCACGACGTCGCGCCGCTT-3′ or C-Rev 5′-CACGACGTCGCGCCGCTTCTT-3′, followed by cloning into the pENTR™/SD/D-TOPO® vector (Thermo Fisher Scientific) as either N- or C- terminal fusion constructs. To generate an N-terminal His-HvCPK2a fusion protein, the *HvCPK2a* clone was recombined with the Gateway® pDEST17™ vector (Invitrogen) using Gateway® LR Clonase® II Enzyme Mix (Invitrogen). To generate variants of *HvCPK2a*, a mutagenesis reaction was performed as described in Mitula et al. (2015) using the following primer pairs: K94M (For 5′-GCCTGCA**T**GACCATCGCCAAG-3′, Rev 5′-CTTGGCGATGGTC**A**TGCAGGC) and D189A (For 5′-CATCCACCGCG**C**CCTCAAGCC-3′, Rev 5′-GGCTTGAGG**G**CGCGGTGGATG-3′). The vector construct 3*5S:HvCPK2a-His-GFP* (Earley et al., 2006) was used for Arabidopsis protoplast transformations and transient expression assays in *Nicotiana benthamiana*. To localize HvCPK2a and mutated versions of the protein in barley protoplasts, we generated a C-terminal HvCPK2a-GFP fusion in the pS5-DEST-EGFP vector using Gateway® LR Clonase® (Invitrogen).

### Protoplast isolation, transformation, and transient expression assays

Transient expression assays in Arabidopsis protoplasts or in tobacco leaves were performed as described in Ludwików et al. (2014) and Mitula et al. (2015). For the preparation of barley protoplasts, the central parts of 20 leaves from 6- to 10-day-old spring barley seedlings were dissected and cut into 0.5 mm strips. Strips were transferred to 10 ml enzyme solution (1.5% cellulase, 0.3% macerozyme, 0.5 M sorbitol, 1 mM CaCl_2_, 10 mM MES pH 5.7) and incubated at 30°C for 3 h in the dark without shaking. After digestion, protoplasts were transferred to 50 ml tubes, centrifuged for 4 min (50 × g, 4°C), washed twice with wash solution (0.5 M sorbitol, 20 mM KCl, 4 mM MES pH 5.7) and resuspended in MMg solution (0.5 M sorbitol, 15 mM MgCl_2_,4 mM MES pH 5.7) to a final concentration of 2 × 10^4^ cells per 100 μl. For protoplast transfections, 5–10 μg plasmid DNA was combined with 100 μl protoplasts and 110 μl PEG solution (40% PEG 4000, 200 mM sorbitol, 10 mM CaCl_2_). After gentle mixing, the solution was incubated for 15 min at RT in a horizontal position. Then, 450 μl W1 buffer (0.5 M sorbitol, 20 mM KCl, 4 mM MES pH 5.7) was added, mixed and centrifuged for 4 min, 50 × g. Supernatant was removed, protoplasts were resuspended in 300 μl W1 solution and incubated overnight at 22°C in the dark in a horizontal position.

### Chlorophyll content

Pigment composition was analyzed using a Dualex 4 leaf-clip optical sensor based on the protocols described in Cerovic et al. (2012, 2015). The leaf chlorophyll content, the flavonoid index and the nitrogen balance index (NBI) were quantified for the upper side of 10 leaves/plant by measuring light transmission absorbed by chlorophyll at 710 nm and in the near-infrared at 850 nm. Flavonoid content was quantified by absorption of UV light. The NBI was then calculated as the chlorophyll to flavonoid ratio, which is directly related to nitrogen/carbon allocation in leaves. We analyzed 3-week old Arabidopsis plants, five plants of each line, and performed 10 measurements on five mature leaves of each plant. Measurements were made on the laminae, avoiding midribs.

### His-HvCPK2a overexpression and purification

*Escherichia coli* Rosetta 2 (DE3) (Novagen) transformed with pDEST17-*HvCPK2a* plasmid was grown overnight in 20 ml LB medium supplemented with 100 μg/ml ampicillin at 37°C with shaking. Twenty ml fresh LB (plus 100 μg/ml ampicillin) were inoculated with 1 ml overnight culture and grown to an optical density (OD_600_) of 0.4–0.6. The culture was then induced with 0.5 mM IPTG and grown for 3 h, bacteria were collected by centrifugation and the pellet stored at −80°C. The pellet was resuspended in lysis buffer (50 mM Tris-HCl pH 7.5, 150 mM NaCl, 0.5% Nonidet P-40, 1 mM PMSF). His-HvCPK2a protein was purified from membrane fractions using Ni Sepharose resin according to standard procedures recommended by the manufacturer (GE Healthcare).

### Immunoblotting

Proteins were fractionated on 10% resolving gels by SDS-PAGE and then transferred onto Immobilon-P membrane (Millipore) by semi-dry transfer. The membranes were blocked in PBS-T (PBS plus 0.01% Tween 20) with 3% skim milk for 1 h with agitation. Next, membranes were washed three times with PBS-T and incubated with primary antibody AS1 (1:1000, AS13 2754 Agrisera), anti-H^+^-ATPase (1:1000, Agrisera), anti-GST (1:6000, MoBiTec Molecular Biotechnology) or anti-GFP (1:200; sc-8334, Santa Cruz Biotechnology) for 1 h with agitation. After washing, the membranes were incubated with appropriate secondary antibodies for 1 h. Detection was performed using ECL (Thermo Scientific) and visualized by G:BOX (Syngene).

## Results

### Identification, sequence comparison, and phylogeny of barley CPKs

Plant CPKs constitute a multigene family, but relatively few CPK family members have been characterized. To identify specific CPK gene copies in barley, we performed a BLASTP search against available databases using full length protein sequences from rice and *Arabidopsis*. A reciprocal analysis was performed to confirm that identified entries indeed gave the best match against the relevant rice and *Arabidopsis* sequences. Gene names and the corresponding accession numbers are indicated in Supplementary Table S1.

To investigate the relatedness of the HvCPKs identified to other known CPKs, a phylogenetic tree was generated including full length protein sequences form *H. vulgare, O. sativa*, and *A. thaliana*. A comparison of kinase domain sequences showed that HvCPKs are distributed very unevenly among the four groups (Supplementary Figure S1). Each group contains CPKs from all three species investigated, and groups are not dominated by particular species. While the first group contains nine HvCPKs, the next three groups (2nd, 3rd, and 4th) include seven, six and two HvCPKs respectively. Interestingly, of the 24 HvCPKs identified, twenty members have orthologous loci in rice. Importantly, OsCPK9 has neither an orthologous nor a paralogous copy in barley, suggesting that the *OsCPK9* gene has evolved since the two plant species diverged.

### Identification of CPKs involved in the drought stress response

**Figure 1:**
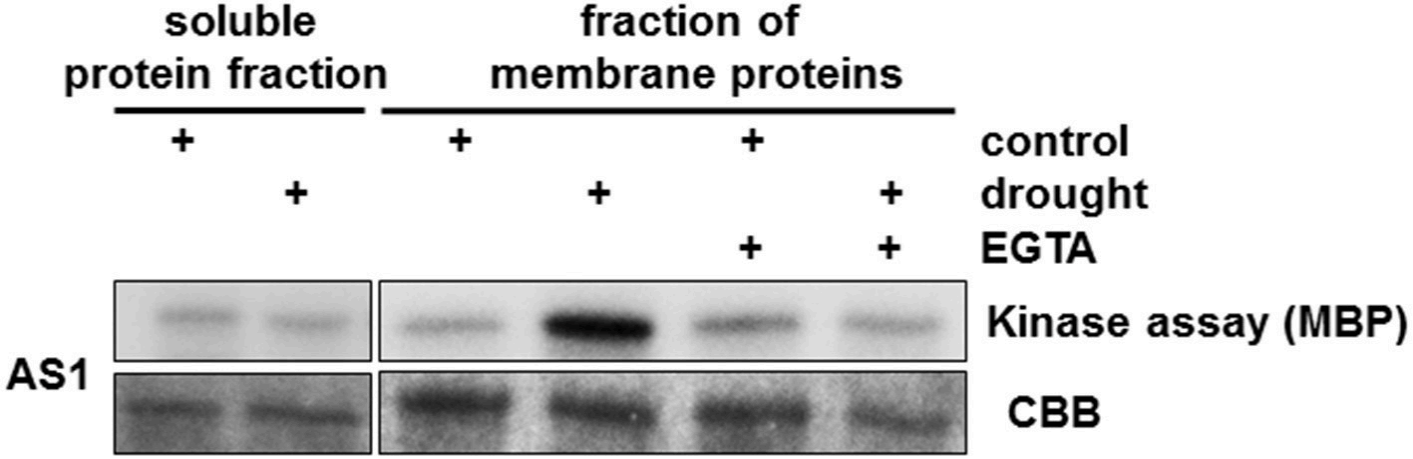
**Identification of drought-induced CPKs in *Hordeum vulgare***. We used both the soluble protein fraction and membrane-bound proteins isolated from a microsomal fraction from drought-stressed barley to define the subcellular location of AS1-associated kinase activity. Strong kinase activity was present in membrane proteins. Treatment with EGTA (Ca^2+^ chelator) abolished HvCPK2a kinase activity. MBP was used as a substrate; Coomassie Brilliant Blue (CBB) staining confirmed equal loading of MBP.

The structure of the CPK proteins is reported to be highly conserved among plants (Hamel et al., 2014). Our results support this: the sequences of HvCPK kinase domains showed remarkable conservation of a 13 amino acid residue sequence in the active site. We hypothesized that this core sequence is likely to be useful for the identification of barley CPKs involved in the drought stress response. Therefore, we raised polyclonal antisera (AS1 and AS2) against two peptides that correspond various regions of the kinase domain; both peptides contain the conserved 13 amino acid core residue. Alignments of HvCPK proteins indicate that they share sequence identity to the AS1 and AS2 peptides in the ranges 76.9–100% and 69.2–100%, respectively (Supplementary Table S2). The AS1 and AS2 antisera were used for the immunoprecipitation (IP) of protein complexes containing CPKs from 6-week-old barley plants subjected to drought stress. AS1 immune complexes contained strong kinase activity, but AS2 did not precipitate active kinases under any conditions used (Supplementary Figure S2). CPKs are localized in various cell compartments, including the cytosol and plasma membrane; therefore, to identify as many putative HvCPKs as possible, we used the soluble protein fraction as well as membrane-bound proteins recovered from sedimenting (microsome) fractions. To confirm that the AS1 antiserum recognizes CPKs, we performed in vitro kinase assays on the resulting immune complexes using MBP as a substrate. AS1 immune complexes identified strong kinase activity in the membrane protein fraction of drought-stressed barley plants (Figure 1).

**Figure 2:**
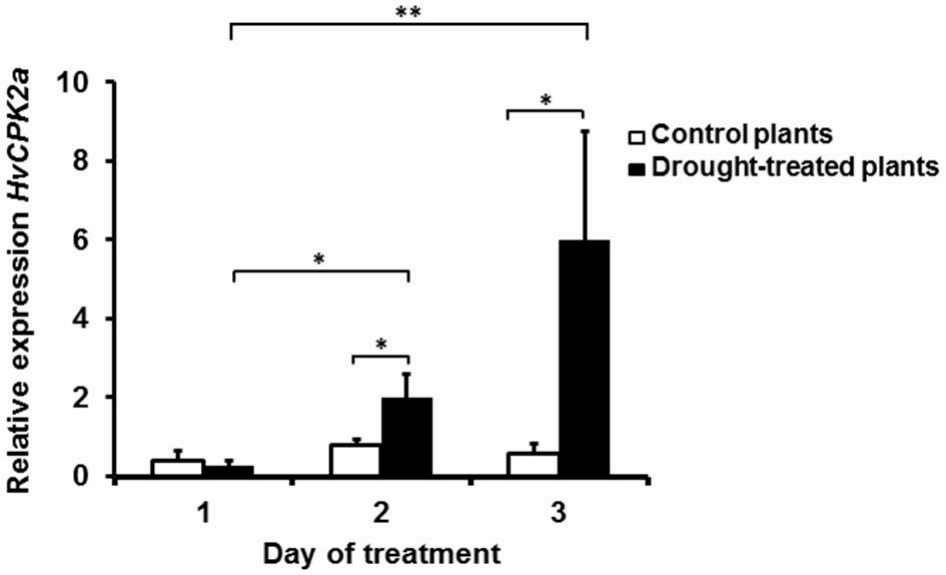
*****HvCPK2a*** gene expression profile in ***Hordeum vulgare*** L. cv. “Sebastian” during drought stress**. Barley *HvCPK2a* gene expression under intensifying drought stress and control conditions: a significant increase in *HvCPK2a* expression occurred on the third day of drought. Relative quantification was determined by qPCR analysis (with ADP-ribosylation factor genes as internal control). Vertical bars correspond to standard errors of the mean. The asterisks indicate significant differences according to Student's *t*-test; ^*^P < 0.05, ^**^P < 0.005.

To identify CPKs within proteins immunoprecipitated using AS1 antibodies, the same immune complexes were analyzed using LC-MS/MS in conjunction with database searching against the TAIR database and the Triticum, Oryza, and Hordeum subsets of the National Center for Biotechnology Information (NCBI). Using this approach, several putative barley CPKs were found in the drought-tolerant cultivar; these included HvCPK24, HvCPK2a, and HvCPK14, which showed 92.7, 95.2, and 92.9% similarity to OsCPK24, OsCPK2, and OsCPK14 from rice, and 77, 85.8, and 84.1% identity to CPK11, CPK34, and CPK17 from Arabidopsis, respectively (Supplementary Table S3). Although none of the putative HvCPKs identified have previously been implicated in the drought response, the HvCPK24-orthologous kinases AtCPK11 and OsCPK24 are known to be involved in drought, high-salt or cold stress responses (Wan et al., 2007). In contrast, the HvCPK2a-orthologous kinases AtCPK34, AtCPK17, and OsCPK2, which are almost undetectable in vegetative tissues but are abundant in pollen, flower or seed tissues, are reported to be critical only for male reproduction and have not been linked to any stress response (Valmonte et al., 2014). To confirm the role of HvCPK2a in the response to drought, total RNA was extracted from drought-stressed barley at different time points. The level of *HvCPK2a* transcript abundance was significantly increased after 3 days of drought (Figure 2), showing that *HvCPK2a* is detectable in leaves collected from control and drought-treated plants. Our results suggest functional divergence between HvCPK2a and its orthologs in Arabidopsis and rice. Therefore, our further analyses concentrate on the role of HvCPK2a.

### Subcellular localization of HvCPK2a

**Figure 3:**
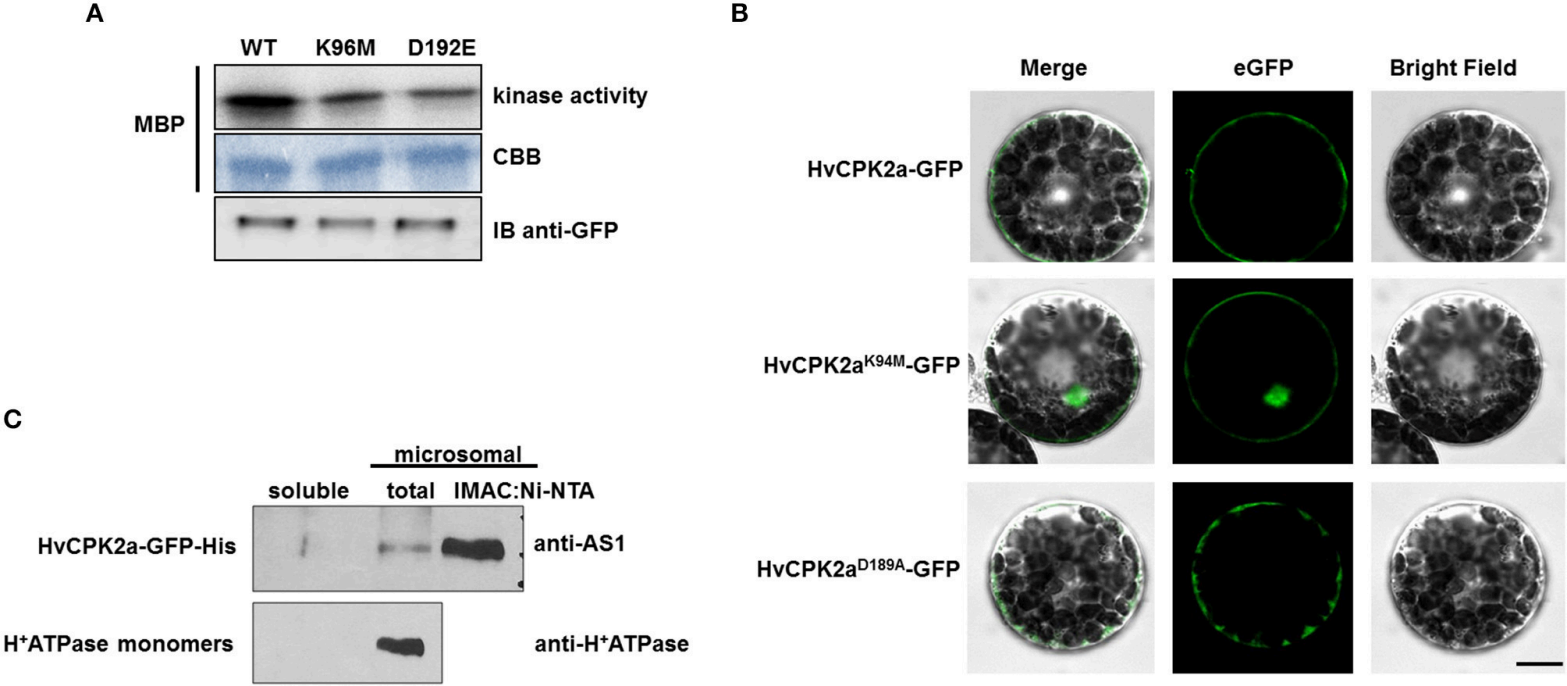
**Subcellular localization of the HvCPK2a-GFP fusion protein. (A)** Microscopy images show that WT HvCPK2a and the D189A variant, visualized as GFP fusion proteins, are associated with the plasma membrane in barley protoplasts. A variant with a single amino acid change within the ATP-binding site (HvCPK2a^K94M^-GFP) showed localization to both membrane and cytoplasm. Scale bar: 10 μm. **(B)** Expression of all three forms of HvCPK2a-GFP in barley protoplasts was confirmed by IB using an anti-GFP antibody. **(C)** HvCPK2a-GFP co-fractionates with the plasma membrane fraction in tobacco plants. Total proteins from tobacco leaves transiently expressing HvCPK2a-GFP-His were separated into soluble and total microsomal fractions. The microsomal fraction, which contained HvCPK2a-GFP-His, was enriched by nickel-affinity chromatography, then separated on a 10% SDS-PAGE gel and transferred to Immobilon-P membrane. H^+^-ATPase monomers of mass 95 kDa, which are known to be membrane-associated, were detected in the microsomal fraction.

HvCPK2a is a canonical CPK with putative myristoylation/palmitoylation sites suggesting that this kinase might be associated with membranes. However, it is known that numerous factors can affect CPK localization, including protein activity, calcium and posttranslational modifications (PTMs) (Hegeman et al., 2006; Schulz et al., 2013; Simeunovic et al., 2016). Because CPKs integrate different signaling cascades, rapid CPK inactivation is crucial for proper signal transduction; therefore, we were interested to know whether the kinase activity of HvCPK2a affects its subcellular localization. Accordingly, constructs for the *in vivo* expression of wild type (WT) HvCPK2a, as well as K94M and D189A variants which are reported to have reduced or abolished kinase activity (Patharkar and Cushman, 2000; Franz et al., 2011; Matschi et al., 2013), were generated. The CPK2^K94M^ version of the protein possesses a single amino acid change in its ATP-binding loop, while the CPK2^D189A^ variant carries a single amino acid change within its kinase active site. C-terminal GFP fusions were transiently expressed under the control of the *35S* promoter in *A. thaliana* protoplasts. We immunoprecipitated the WT, K94M, and D189A versions of HvCPK2a from protoplasts extracts and confirmed that both mutants have significantly reduced kinase activity compared to WT. The presence of all three HvCPK2a versions was demonstrated by immunoblotting (Figure 3A).

Next, we determined the subcellular localization of HvCPK2a variants in barley protoplasts. Because we were unable to transform barley protoplasts using pEarlyGate vectors, we generated a separate set of vector constructs that facilitated protein localization in a barley transient expression system. Thus, coding sequences for WT HvCPK2a and the two mutant forms were expressed in pS5N3 vectors under control of the UBQ promoter as C-terminal fusions. We observed WT HvCPK2a and both variants localized to the plasma membrane, but the variant with a mutation in the ATP-binding site (K94M) was also found in the nucleus (Figure 3B), suggesting that HvCPK2a is important for the amplification of calcium signals in various cellular compartments. A similar distribution was observed in Arabidopsis as in barley protoplasts: as shown in Supplementary Figure S3, WT HvCPK2 was mostly located along the inner face of the plasma membrane, as was the D189A mutant. Interestingly, however, the K94M variant localized to the nucleus as well as the plasma membrane.

The membrane localization of HvCPK2a was further demonstrated by AS1 immunoblotting of protein extracts from tobacco leaves expressing HvCPK2a-GFP-His (Figure 3C). We identified the HvCPK2a fusion protein in the total microsomal fraction, and also after enrichment of this fraction by nickel-affinity chromatography, but not in the soluble protein fraction. Antibodies against H^+^-ATPase confirmed the presence of membrane proteins in the microsomal fraction. The localization of the barley kinase is supported by a previous finding showing that the HvCPK2a orthologous kinase AtCPK34 is also associated with the plasma membrane (Myers et al., 2009).

### Identification of HvCPK2a-interacting proteins

Despite the importance of CPKs in plants, relatively little is known about their substrates. To improve our understanding of the HvCPK2a signal transduction pathway, we attempted to identify proteins that interact with HvCPK2a using LC-MS/MS. For this purpose, we expressed HvCPK2a-GFP-His in a *Nicotiana benthamiana* transient expression system. Fully developed leaves from 4-week-old tobacco plants were infiltrated with *Agrobacterium* carrying a *35S:HvCPK2a-His-GFP* construct and plant material was collected 5 or 6 days later. As a control, non-infiltrated tobacco leaves were used. Because HvCPK2a is associated with membranes, we recovered HvCPK2a-GFP-His protein from the microsomal fraction using nickel-affinity chromatography and subjected co-purifying proteins to LC-MS/MS. WT tobacco leaves were processed similarly as a negative control.

We initially used the tobacco database with LC-MS/MS data to attempt to identify proteins that interact with HvCPK2a, but this approach mainly highlighted proteins connected with the plant response to *Agrobacterium* infection. Therefore, in further analysis we used the TAIR10 Arabidopis database and as a result identified 29 candidate HvCPK2a-interacting proteins that were not present in control samples (Supplementary Table S4). Identification of AtCPK34 validated the method used to purify HvCPK2a. The most abundant proteins found in HvCPK2a complexes were citrate synthase 5 (CSY5), sucrose synthase 4 (SUS4), NAD-dependent malic enzyme 1 (NAD-ME1), selenium binding protein 1 (SBP1), formate dehydrogense (FDH), 40S ribosomal protein SA B (RSAB), chlorophyll A-B binding family protein (NPQ4), GTP-binding elongation factor Tu family protein and glutathione S-transferase TAU23. In four of the seven MS/MS runs we identified pyruvate orthophosphorate dikinase (PPDK), indole-3-butyric acid response 1 (IBR1), BHB domain containing membrane associated protein family, translation elongation factor EF1B, adenine nucleotide alpha hydrolases-like superfamily protein, beta-ureidopropionase and aldehyde dehydrogenase 2B4. Among proteins identified in three or fewer samples were serine hydroxmethyltransferase 2 (SHM2), two members of the family of 90 kDa heat shock proteins (HSP90.2/ERD8 and HSP90.7), phospholipase D alpha 1 (PLDALPHA1) and two isoforms of the H^+^ATPase family, AHA1 and AHA3.

The candidate proteins listed above were analyzed further with regards to their role in biological processes. The majority of these proteins are known to play essential roles in the stress responses to salt, drought, cold and wounding. These stress-responsive proteins included early response to dehydration (HSP90.2/ERD8), an ER-resident HSP90-like protein (SHD), NAD-ME1, SBP1, UDP-glucose:glycoprotein glucosyltransferases (EBS1, UGGT, PSL2), SUS4 and formate dehydrogense (FDH). Other interesting candidates were proteins involved in ABA signaling (H^+^-ATPase 1, PLDALPHA1) and development (PLDALPHA1; IBR1; aspartate aminotransferase 3). These results suggest that HvCPK2a is involved in a variety of cellular functions. More information on this is given in Supplementary Table S4.

### HvCPK2a overexpression reduces tolerance to drought in transgenic arabidopsis

**Figure 4:**
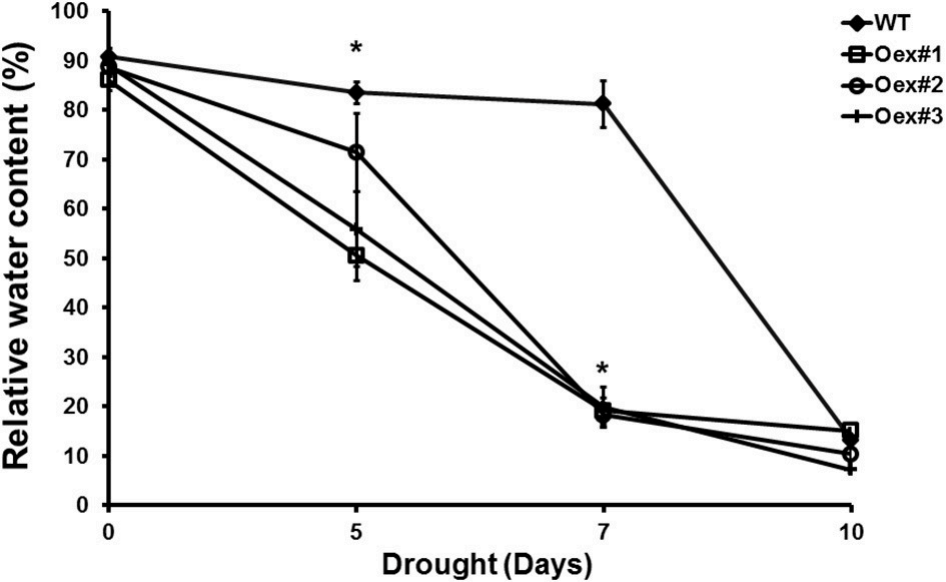
**Drought-stressed HvCPK2a overexpression lines exhibit reduced water capacity**. Relative water content of WT and Oex lines in response to drought stress: drought-stressed transgenic plants lose more water than WT. Data shown are the means of 10 measurements taken from 10 plants ±SE from two independent experiments. The asterisks indicate significant differences in Oex lines compared with WT Col-0 according to Student's *t*-test; ^*^P < 0.01.

To confirm a role for HvCPK2a in the drought stress response we generated transgenic *A. thaliana* lines that overexpress *HvCPK2a*. Ten independent lines were selected and all were included in further analysis; representative results of three lines are shown. There was no difference in growth between WT and *HvCPK2a*-overexpressing plants, but after exposure to drought, *HvCPK2a*-overexpressing plants (Oex lines #1–3) exhibited lower leaf relative water content (RWC) and lower survival rates than WT plants (Figure 4), indicating that *HvCPK2a*-overexpression reduces tolerance to drought.

**Figure 5:**
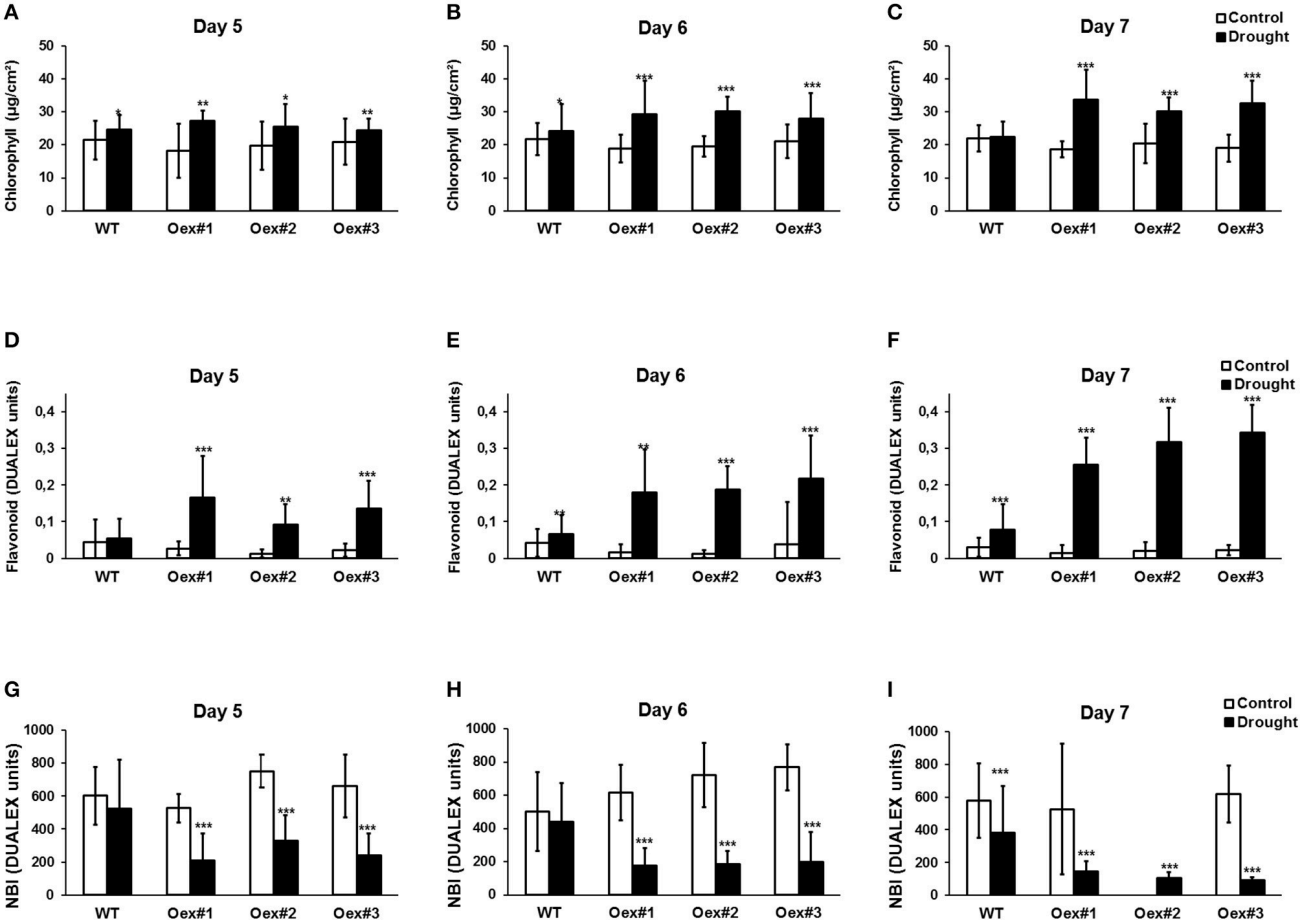
**Overexpression of ***HvCPK2a*** in Arabidopsis reduces plant tolerance to drought**. Drought-stressed *HvCPK2a*-overexpressing plants have higher Chl index **(A–C)**, higher flavonoid content **(D–F)** and reduced NBI index **(G–I)** compared to WT plants. Data represent mean ± SE; *n* = 10 plants per line. The asterisks indicate significant differences in Oex lines compared with WT Col-0 according to Student's *t*-test; ^*^P < 0.01; ^**^P < 0.001; ^***^P < 0.0001.

Chlorophyll and flavonoid levels can be used to monitor senescence and both are good indicators of nitrogen status in plants (Agati et al., 2015, 2016). Therefore, we used a Dualex 4 optical sensor to investigate the influence of drought on chlorophyll (Chl index) and flavonoid (FLAV index) content, which also allowed us to calculate the NBI. As indicated in Figures 5A–C, the Chl index significantly increased during the period of drought in both WT and *HvCPK2a*-overexpressing lines #1–3. This is particularly clear on the 6th and 7th days of drought, when dehydration becomes more pronounced: the Chl index is significantly higher in *HvCPK2a*-overexpressing lines than in WT (Figures 5B,C). Next, we analyzed the FLAV index, and found that, as drought progressed, it also significantly increased in *HvCPK2a*-overexpressing lines compared to WT (Figures 5D–F), becoming three times higher than WT after 7 days (Figure 5F). For the NBI, there was no difference between *HvCPK2a*-overexpressing lines #1–3 and WT under fully hydrated conditions (Figure 5G), but after drought was imposed the NBI decreased significantly for all Oex lines compared to WT (Figures 5H,I). Taken together, these data indicate that the drought-sensitive phenotype of *HvCPK2a*-overexpressing plants is associated with reduced water retention capacity and diversification of specialized secondary metabolism.

### Recombinant HvCPK2a is regulated by autophosphorylation

**Figure 6:**
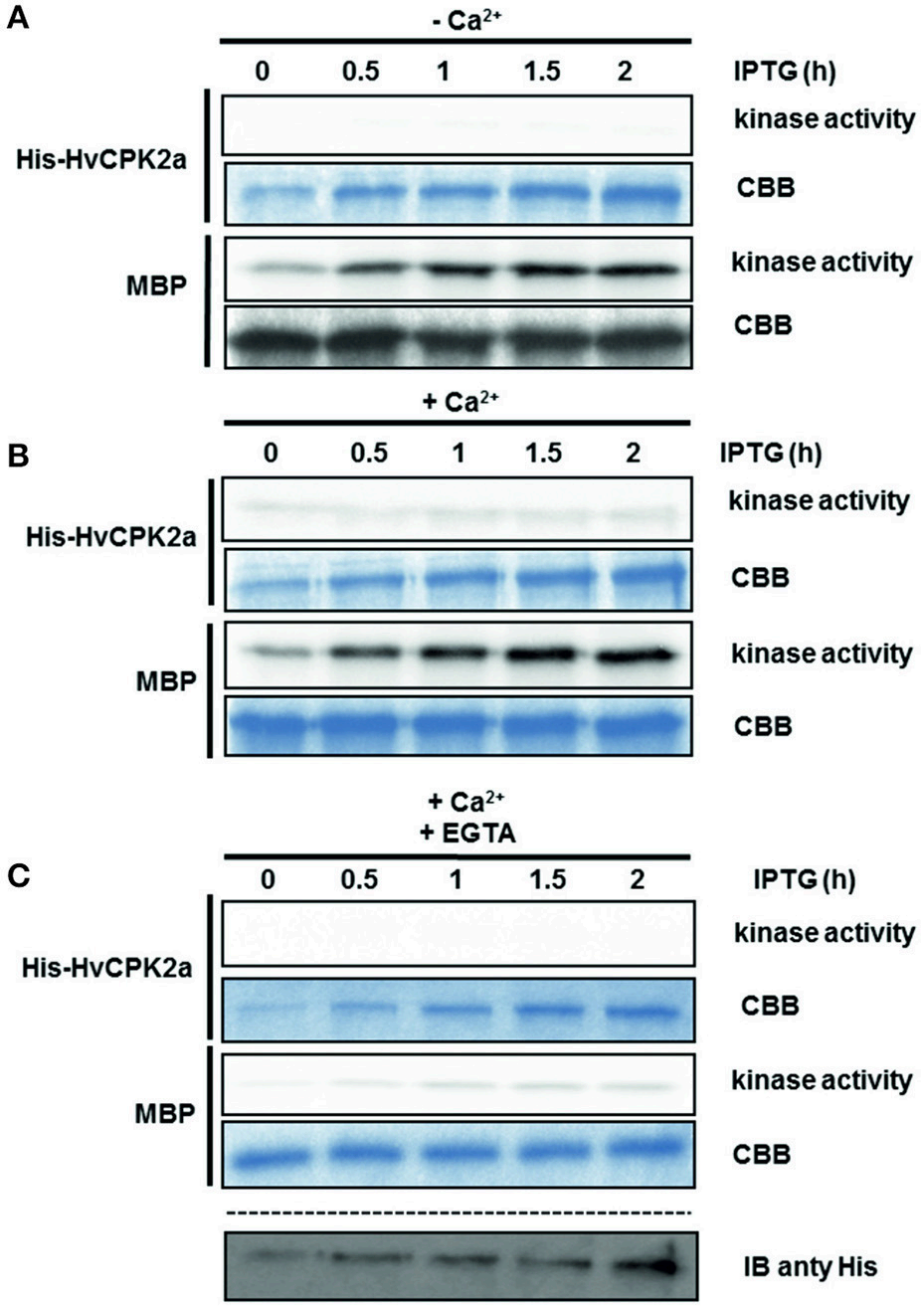
**Autoactivation of recombinant His-HvCPK2a**. Recombinant protein expression was induced by IPTG, collected at the indicated time points, then analyzed for kinase activity using MBP as artificial substrate in the presence **(B–C)** or absence **(A)** of Ca^2+^. EGTA was used as a calcium chelator to prevent calcium-dependent phosphorylation. Radioactivity of MBP bands was measured with a phosphoimager. Coomassie Brilliant Blue (CBB) staining confirmed equal loading. His-HvCPK2a was detected using anti-His antibody.

Recent studies indicate that the activity of CPK kinases is regulated by autophosphorylation (Chaudhuri et al., 1999; Harmon et al., 2000; Oh et al., 2012). To test whether HvCPK2a kinase is activated by autophosphorylation, His-tagged HvCPK2a protein was affinity purified and used in a kinase assay with MBP as a substrate. As shown in As shown in Figure 6A, at the initial time point His-*Hv*CPK2a was highly active and, accordingly, we did not observe a significant increase in kinase activity (with MBP as substrate) in the presence of Ca^2+^, suggesting that His-HvCPK2a autophosphorylates in bacterial systems. To confirm this observation, we monitored His-HvCPK2a activity in bacterial cultures after IPTG induction in a time course experiment. In the absence of Ca^2+^, we observed phosphorylation of the MBP substrate by His-HvCPK2a as soon as 30 min after IPTG induction. At the same time, de novo autophosphorylation of His-HvCPK2a was barely noticeable. Moreover, in the presence of Ca^2+^ ions there was a substantial increase in MBP substrate phosphorylation, as well as an increase in autophosphorylation of His-HvCPK2a (Figure 6B). When His-HvCPK2a was incubated with both Ca^2+^ and EGTA, a significant attenuation of kinase activity against MBP was observed. In line with this, there was nearly no His-HvCPK2a kinase activity against MBP in the presence of the calcium chelator, suggesting that His-HvCPK2a activity is indeed calcium dependent (Figure 6C). Overall, these results demonstrate that *HvCPK2a* encodes a functional calcium-dependent protein kinase.

**Figure 7:**
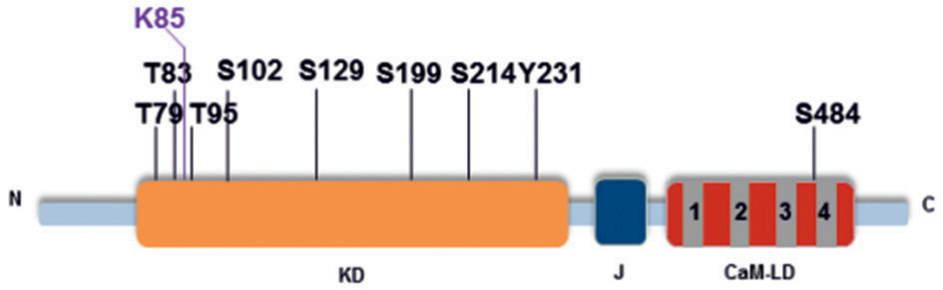
**HvCPK2a autophosphorylation sites**. Schematic of HvCPK2a showing the location of phosphorylation sites identified by LC-MS/MS. The conserved kinase domain (KD) is shown in orange, the junction domain (J) in black and the calmodulin-like domain (CaM-LD) in gray and red. Seven out of the eight phosphorylated residues identified were located within the kinase domain (KD) and one in the calmodulin-like domain in close proximity to the fourth EF-hand structure. Lysine 85 (K85) was identified as a ubiquitination site.

We used mass spectrometry to identify autophosphorylation sites and found nine phosphorylated residues in the activated His-HvCPK2a, comprising three threonine residues (T79, T83, and T95), five serine residues (S102, S199, S214, S229, and S484) and one tyrosine residue (Y231) (Table 1; Data Sheet 1). Seven of the first eight residues listed, namely T79, T83, T95, S102, S199, S214, and S229, were located in the kinase domain. The eighth residue, S484, was identified within the calmodulin-like domain, toward the C-terminus (Figure 7). As shown in Table 1, the most abundant phosphorylation sites were T95 and S229. One of these, T95, was identified in the immediate proximity to K94, a conserved lysine that is crucial for ATP-binding in CPKs (Sheen, 1996). Interestingly, the HvCPK2a homolog AtCPK34 is thought to be a dual specificity kinase (Oh et al., 2012, 2013), which is in accord with the identification of a single autophosphorylated tyrosine residue (Y231) in HvCPK2a, indicating that the barley protein does have tyrosine kinase activity. As well as phosphorylation sites, we also found a single ubiquitination event on K85 (Figure 7), suggesting that HvCPK2a undergoes proteasomal degradation.

**Table 1:** **Identification of His-HvCPK2a autophosphorylation sites**.

**Spectra count**		**Sites/ residues**	**Score**	**Observed**	**Mr (expt)**	**Mr (calc)**	**Delta**	**M**	**Expect**	**U**	**Peptide**
***In vivo* autophosphorylation sites**	***In vitro* autophosphorylation sites**										
1	0	T79	37	722.8186	1443.6226	1443.6040	0.0186	0	0.024		R.GQFGVTHLC**pT**QK.A + Phospho (ST)
2	0	T83, K85	37.0	793.3122	1584.6098	1584.6354	−0.0256	0	0.017	U	R.GQFGVTYLC**pT**E**ubqK**.S + GlyGly (K); Phospho (ST)
34	56	T95	57.5	504.2228	1006.4310	1006.4381	−0.0071	1	0.0041		Q.FACK**pT**IAK.R + Phospho (ST)
0	5	S102	66.4	755.8621	1509.7096	1509.7440	−0.0343	2	0.0011		R.KLI**pS**KEDVEDVR.R + Phospho (ST)
3	14	S199	71	748.8919	1495.7693	1495.7687	0.0006	0	0.00027	U	R.DLKPENFLLL**pS**K.D + Phospho (ST)
1	2	S214	76.7	656.3063	1310.5981	1310.5948	0.0033	0	9.1e-05	U	K.ATDFGL**pS**VFFK.P + Phospho (ST)
171	256	S229	114	859.4178	1716.8211	1716.8375	−0.0164	0	2.3e-08	U	K.DIVG**pS**AYYIAPEVLK.R + Phospho (ST)
0	14	Y231	106	859.4266	1716.8386	1716.8375	0.64	1	1.5e-07	U	K.DIVGSA**pY**YIAPEVLK.R + Phospho (Y)
6	9	S484	54.7	749.8043	1497.5940	1497.5984	−0.0044	0	0.013	U	K.EIL**pS**DVDADNDGR.I + Phospho (ST)

*Using LC MS/MS we identified nine autophosphorylation sites within recombinant HvCPK2a. Phosphorylation of residue T83 and ubiquitination of K85 were observed in the same peptide. Spectra count—the number of spectra assigned per peptide; Score—Mascot Score is the log of a probability that the observed match is a random event [–10log_10_(P), where P is the absolute probability], Observed—experimental m/z value, Mr (expt)—experimental m/z transformed to a relative molecular mass, Mr (calc)—calculated relative molecular mass of the matched peptide, Delta—difference (error) between the experimental and calculated masses, M—the number of missed cleavages, Expect—expectation value, directly equivalent to the E-value in a Blast search (the lower the value, the better), U—unique peptide for the protein hit, Peptide—sequence of identified phosphopeptides. Spectra for each peptide are included in Data Sheet 2*.

## Discussion

Calcium ions are one of the most important second messenger signaling systems in all eukaryotes. Plants have evolved a multigene family of CPKs, which are involved in development, stress responses and plant hormone signaling (Ludwig et al., 2004; Yoon et al., 2006; Kobayashi et al., 2007; Ma and Wu, 2007; Myers et al., 2009; Mehlmer et al., 2010; Xu et al., 2010; Asano et al., 2011, 2012; Boudsocq and Sheen, 2013). However, in barley, relatively few biological functions have been assigned to CPK family members. In the present study, we explored the possible role of HvCPK2a in the drought stress response. We showed that the *HvCPK2a* transcript level increases in response to drought stress treatment (Figure 2), but that overexpression of HvCPK2a increases drought sensitivity (Figures 4, 5). We also found that HvCPK2a-GFP and HvCPK2a^*D189A*^-GFP fusion proteins are associated with the plasma membrane (Figure 3A). Moreover, proteins that putatively interact with HvCPK2a are localized in various cellular compartments. Finally, we demonstrated that HvCPK2a is a dual-specificity kinase that autophosphorylates at T79, T83, T95, S102, S199, S214, S229, S484, and Y231 residues (Table 1; Figure 7).

The role of calcium-dependent kinases in the response to drought is best characterized in Arabidopsis and rice (Saijo et al., 2000, 2001; Ma and Wu, 2007; Zou et al., 2010). There are also examples of CPKs involved in drought signaling in *Vicia faba* (Liu et al., 2006) and wheat (Li et al., 2008). To identify barley CPKs regulated by drought, we used targeted antibodies and identified CPK activity in immune complexes from drought-stressed samples. Using LC-MS/MS, we identified HvCPK2a and HvCPK14 as drought-induced CPKs, although their Arabidopsis homologs are predominantly expressed in mature pollen (Harper et al., 2004; Myers et al., 2009). In contrast to the situation in Arabidopsis, we found that *HvCPK2a* is expressed in leaves in response to drought stress (Figure 2). Interestingly, the sequence identity of HvCPK2a and its rice homologs is rather high (~90%), and only slightly lower (~85%) for HvCPK2a and its Arabidopsis homolog. We conclude therefore that the apparent functional divergence is more likely due to differences in promoter regulatory sequences, resulting in divergent expression patterns between species. However, it cannot be excluded that variation in amino acid sequence may add an additional level of functional diversification, for example, by modifying target protein binding (Dun et al., 2014).

We investigated the role of HvCPK2a in the drought stress response using two approaches. In the first approach, we generated Arabidopsis plants that constitutively overexpress barley *CPK2a*. The transgenic plants were less able to adapt to adverse conditions compared to WT plants, indicating that HvCPK2a is a negative regulator of tolerance to drought stress. It has been reported that Arabidopsis CPK23 negatively regulates drought resistance by affecting ion transport (Ma and Wu, 2007). Importantly, *HvCPK2a*-overexpressing plants have a reduced RWC (Figure 4) and NBI (Figure 5), suggesting an important role for *HvCPK2a* in the regulation of leaf growth and biomass under drought conditions.

In the second approach, we investigated in detail how HvCPK2a contributes to regulation of the drought stress response by looking for proteins that interact with the barley kinase. Using LC-MS/MS, we identified several putative HvCPK2a-interacting partners including a membrane-localized H^+^-ATPase, which has been shown to be regulated by CPKs (Schaller and Sussman, 1988; Xing et al., 1996; Camoni et al., 1998a,b; Lino et al., 1998; De Nisi et al., 1999; Kinoshita and Shimazaki, 1999; Rutschmann et al., 2002), a glutathione S-transferase (GST), which is crucial in the response to various stress conditions (Bartlling et al., 1993; Cummins et al., 2011; Kumar et al., 2013), and a selenium binding protein (AtSBP) involved in tolerance to cadmium and selenium (Dutilleul et al., 2008; Hugouvieux et al., 2009; Schild et al., 2014). Another protein identified, a sucrose synthase (SUS), was found to be involved in long-term adaptation to drought stress conditions (Déjardin et al., 1999; Baud et al., 2004). Importantly, SUS protein sequences contain a predicted motif for CPK-mediated phosphorylation (Huber et al., 1996; Nakai et al., 1998; Zhang et al., 1999; Komina et al., 2002; Hardin et al., 2003, 2004; Fedosejevs et al., 2014). For example, maize SUS1 has two conserved phosphorylation sites for CPK (Ser-15 and Ser-170). N-terminal phosphorylation of Ser-15 stimulates SUS1 activity and affects its membrane association. On the other hand, S170 phosphorylation is thought to be involved in the proteolytic turnover of SUS1 (Hardin et al., 2003). N-terminal phosphorylation of SUS1 by CPK has also been demonstrated in *Ricinus communis*. These data suggest that the drought stress response in barley might be controlled in the long term by HvCPK2a phosphorylation of SUS.

Other enzymes identified in HvCPK2a complexes were formate dehydrogenase (FDH), NAD-dependent malic enzyme (NAD-ME) and pyruvate orthophosphate dikinase (PPDK). Recent studies showed that FDH contributes to stress responses; for example, it is involved in the response to iron deficiency in barley roots, and to osmotic stress in potato (Suzuki et al., 1998; Ambard-Bretteville et al., 2003). NAD-ME provides substrates for the tricarboxylic acid cycle and therefore belongs to a key pathway involved in carbon metabolism. In drought-stressed *Amaranthus cruentus*, NAD-ME activity is increased, although the protein level remains stable (Guo et al., 2009; Babayev et al., 2014), suggesting that enzyme activity might be regulated by posttranslational modifications. In rice, PPDK, also known for its role in C4-plant photosynthesis, is regulated by ABA and water deficit (Moons et al., 1998). Overall, these results indicate that HvCPK2a integrates metabolism (formate, malic and pyruvate metabolism) to sustain plant growth and yield during stress conditions (Supplementary Table S4).

To understand the role of HvCPK2a in the drought stress response, we examined the mechanism of its activation (Figure 6). CPKs are classified as serine/threonine protein kinases, but there are data demonstrating that CPKs are dual-specificity kinases, which autophosphorylate their own Ser/Thr and Tyr residues (Oh et al., 2012). Our data demonstrate that HvCPK2a is a Ca^2+^-dependent dual-specificity kinase that autophosphorylates in bacteria on T, S and Y residues localized within the catalytic (T79, T83, T95, S102, 199, S214, S229, Y231) and a calmodulin-like domain (S484) (Figure 7; Data Sheet 2). Previous studies have demonstrated that phosphorylation of the catalytic domain CPK is critical for kinase activity (Chaudhuri et al., 1999; Chehab et al., 2004; Oh et al., 2012). As eight out of nine HvCPK2a autophosphorylation sites are within the catalytic domain, it will be important to determine in future studies which specific phosphosites are kinase-activating. The function of phosphorylation within the calmodulin-like domain is currently unknown and likewise deserves investigation, particularly as corresponding phosphosites were found in tomato LeCPK1 (Ser-439) and ice plant MeCPK1 (Ser-420) (Rutschmann et al., 2002; Chehab et al., 2004).

In summary, this study provides new insights into the role of barley CPKs in the drought stress response. Compared to its Arabidopsis homologs, HvCPK2a has apparently acquired different, but key functions in the regulation of drought tolerance. The results presented here provide the basis for further analysis of CPK2a functions in barley.

## Author contributions

AC, FM, LM, OF, SJ, MT, MM, and AL conceived the study, carried out the experiments and analyzed the data. MT and MJ assisted in manuscript data acquisition and analysis. AC and MJ were involved in manuscript data deposition. AL and JS participated in study design and coordination. FM, LM and OF participated in drafting the article. AC and AL wrote the manuscript. All authors revised and approved the final manuscript.

### Conflict of interest statement

The authors declare that the research was conducted in the absence of any commercial or financial relationships that could be construed as a potential conflict of interest.

## Supplementary material

Supplementary Table S1 **Gene names and accession numbers**.

Supplementary Table S2 **Amino acid sequence identity (%) between AS1 and AS2 peptides and ***Hv***CPK sequence, respectively of the conserved residues of the active site located in kinase domain region**. The *Hv*CDPK2a protein selected for further analyses is highlighted in bold.

Supplementary Table S3 **Comparison of sequence similarity and identity between CPKs using SIAS method**.

Supplementary Table S4 **Proteins identified in His-HvCPK2a complexes**.

Data Sheet 1 **Multiple sequence alignment**.

Data Sheet 2 **MS/MS spectra of phosphopeptides in Table 1**.

Supplementary Figure S1 **Phylogenetic relationship of selected plant calcium-dependent protein kinases**. A neighbor-joining phylogenetic tree with 1000 bootstrap replications was generated with MEGA6 software using the full-length ORFs of 34 Arabidopsis (shown in red), 24 rice (green), and 24 barley (blue) CPK isoforms. Amino acid sequences were obtained from NCBI and aligned using Clustal W.

Supplementary Figure S2 **To identify drought-induced CPKs, we used custom-prepared antibodies (AS1 and AS2) against the conserved kinase domain**. Antibodies were used to immunoprecipitate protein complexes containing CPKs from drought-treated barley. CPK enzyme activities associated with immune complexes were identified using an *in vitro* kinase assay. Myelin basic protein (MBP) was used as substrate. The AS1 immune complex revealed strong kinase activity in the drought-treated sample, while the AS2 immune complex showed no kinase activity under either control and drought conditions. CBB staining confirmed equal loading of MBP.

Supplementary Figure S3 **Subcellular localization of HvCPK2a in Arabidopsis protoplasts**. The WT version of the HvCPK2a-GFP fusion protein is associated with the plasma membrane. HvCPK2a^K94M^-GFP is also present in the nucleus, while the HvCPK2a^D189A^ variant is present in the cytoplasm and on the cytoplasmic side of the plasma membrane. Hoechst H33342 was used as nuclear localization marker. Scale bar: 10 μm.
