# Supplementary material for: A Role for Barley Calcium-Dependent Protein Kinase CPK2a in the Response to Drought
Source: Front Plant Sci. 2016 Oct 25;7:1550. doi: 10.3389/fpls.2016.01550 (PMC5078816; doi:10.3389/fpls.2016.01550)
Supplement: Supplementary Table S1 — Gene names and accession numbers. [file Table1.DOCX]

**Supplementary Table S1.** Gene names and accession numbers.

| **gi number** | **HvCPK** |
| --- | --- |
| AK373165 | CPK17 |
| AK373462 | CPK2 |
| MLOC_59921 | CPK28 |
| MLOC_6391 | CPK11a |
| MLOC_6934 | CPK6a |
| BAJ86092 | CPK5 |
| BAJ86849 | CPK11 |
| BAJ88027 | CPK8 |
| BAJ91363 | CPK6 |
| BAJ93361 | CPK4 |
| BAJ94561 | CPK15 |
| BAJ95679 | CPK1 |
| BAJ96062 | CPK5a |
| BAJ96261 | CPK7 |
| BAJ96684 | CPK12 |
| BAJ99143 | CPK3 |
| BAK02486 | CPK24 |
| BAK03003 | CPK10 |
| BAK05737 | CPK29 |
| BAK05906 | CPK21 |
| **BAK06618** | **CPK2a** |
| BAK06838 | CPK25 |
| BAK07213 | CPK22 |
| BAK07881 | CPK14 |
