# Supplementary material for: A Role for Barley Calcium-Dependent Protein Kinase CPK2a in the Response to Drought
Source: Front Plant Sci. 2016 Oct 25;7:1550. doi: 10.3389/fpls.2016.01550 (PMC5078816; doi:10.3389/fpls.2016.01550)
Supplement: Supplementary Table S2 — Amino acid sequence identity (%) between AS1 and AS2 peptides and HvCPK sequence, respectively of the conserved residues of the active site located in kinase domain region. The HvCDPK2a protein selected for further analyses is highlighted in bold. [file Table2.DOCX]

**Supplemental Table S2.** Amino acid sequence identity (%) between AS1 and AS2 peptides and *Hv*CPK sequence of the conserved residues of the active site located in kinase domain region. The *Hv*CPK2a protein selected for further analyses is highlighted in bold.

| **gi number** | **% identity to AS1 peptide** | **% identity to AS2 peptide** |
| --- | --- | --- |
| AK373165 | 100 | 76.9 |
| AK373462 | 100 | 76.9 |
| MLOC_59921 | 100 | 76.9 |
| MLOC_6391 | 100 | 76.9 |
| MLOC_6934 | 92.3 | 69.2 |
| BAJ86092 | 69.23 | 100 |
| BAJ86849 | 69.23 | 100 |
| BAJ88027 | 100 | 76.9 |
| BAJ91363 | 92.3 | 76.9 |
| BAJ93361 | 92.3 | 76.9 |
| BAJ94561 | 100 | 76.9 |
| BAJ95679 | 92.3 | 76.9 |
| BAJ96062 | 76.9 | 69.2 |
| BAJ96261 | 100 | 76.9 |
| BAJ96684 | 76.9 | 69.2 |
| BAJ99143 | 92.3 | 69.2 |
| BAK02486 | 100 | 76.9 |
| BAK03003 | 84.61 | 76.9 |
| BAK05737 | 92.3 | 69.2 |
| BAK05906 | 84.61 | 76.9 |
| **BAK06618** | 76.9 | 69.2 |
| BAK06838 | 92.3 | 69.2 |
| BAK07213 | 100 | 76.9 |
| BAK07881 | 76.9 | 69.2 |
