# Supplementary material for: A Role for Barley Calcium-Dependent Protein Kinase CPK2a in the Response to Drought
Source: Front Plant Sci. 2016 Oct 25;7:1550. doi: 10.3389/fpls.2016.01550 (PMC5078816; doi:10.3389/fpls.2016.01550)
Supplement: Data Sheet 1 — Multiple sequence alignment. [file DataSheet1.PDF]

CLUSTAL 2.1 multiple sequence alignment

```
HvCPK6a         MG----RGAG--------------------------------------------------
OsCPK6          MGNYYSCGASSTSSPTSPSLVDYYYC---------------------YHRYPSSCSST--
HvCPK4          MGNACG-GSLRSKYLHSFKHPASQRHDPDRDYDPN--LNPNPAAAADSPKK-TKP--A--
OsCPK13         MGNACG-GSLRSKYLS-FKQTASQRHDTD---------DNNNAAAADSPKKPSRPPAA--
HvCPK5a         MGNTCG-VTFRSKYFSSFRG-ASQRHDSG--------YAPVAADADADSQPGKRPSR---
OsCPK5          MGNTCG-VTLRSKYFASFRG-ASQRHDEAG-------YAPVATSAAAAAAADEPAGKK--
AtCPK5          MGNSCR-GSFKDKLDEGDNNKPEDYSKTSTTNLSSNSDHSPNAADIIAQEFSKDNNSN--
AtCPK6          MGNSCR-GSFKDKIYEGNHSRPEENSKSTTTTVSS--VHSP----TTDQDFSKQNTN---
AtCPK26         MGLALF-SSDGKLIWKG----------STQTGKRR-------------------------
OsCPK7          MGNQCQNGTLGSDYHNRFPREHAVGY-------------------VQGDSYLDLKKFD--
OsCPK23         MGNSCQNGTYGNNYQNSNRFQNDR----------------------FASRYVDGNDTE--
HvCPK2          ------------------------------------------------------------
HvCPK24         ------------------------------------------------------------
OsCPK24         ------------------------------------------------------------
HvCPK28         ------------------------------------------------------------
OsCPK28         ------------------------------------------------------------
AtCPK4          ------------------------------------------------------------
AtCPK11         ------------------------------------------------------------
AtCPK12         ------------------------------------------------------------
HvCPK17         MGNTCSGPSATPDRHGFF------NTVAVAVLWRPGAARAEPAVPQ--PNSCP-------
OsCPK17         MGNTCVGPSSAADRHGFF------HSVSLAVLWRPGG-RAEPSQPPGYPPRES-------
HvCPK11a        ------------------------------------------------------------
OsCPK11         MGNNCVGPS-AAGQNGFF------ANVALWRPRPADAAPPALPPPSSAPSDQAP------
HvCPK8          MGNTCVGPS--IGKNGFF------HSVSTVLWTRRPDGD-ALPAATANANGGVADRAPPP
OsCPK10         MGNTCVGPS--ISKNGFF------QSVSTVLWKARQDGDDALPGANGAPDGGGQGRLPAP
OsCPK27         MGNVCIGPRRNFAKNGLLGILRPRHAAPSSPSQPTTTSRSIPVVLPSAPSSKPPPPTQTA
AtCPK20         MGNTCVGPN--LNPNGFL------QSVSAAVWRNQKPDDSIKSSKDESSRKKNDKSVNGD
AtCPK1          MGNTCVGP----SRNGFL------QSVSAAMWRPRDGDDSASMS---NGDIASEAVSGEL
AtCPK2          MGNACVGPN--ISGNGFL------QTVTAAMWRPRIGAEQASSSSHGNGQVSKEAAS---
AtCPK25         MGNVCVHMVNNCVDTKSNSWVRPTDLIMDHPLKPQLQDKPPQPMLMNKDDDKTKLNDTHG
HvCPK15         MGGRAS-------RQRSDP-----------------------PPPQAQQTPKQPNWPRPR
OsCPK15         MGARAS-------RHRQSPDQSQSQ----------------SPSPHHKHHHHHQTTRAPK
HvCPK1          MGNRTS-------RHRRAAA----------------------DQPAAAPPPTAQSKPQPQ
OsCPK1          MGNRTS-------RHHRAAP----------------------EQPPPQPKPKPQPQQQQQ
AtCPK3          MGHRHS-------KSKSSDP---------------------PPSSSSSSSGNVVHHVKPA
HvCPK2a         MGNCCA-------GSGDAEPV-------------------------PASSGDPSTRRA--
OsCPK2          MGNCCP-------GSGDAEPA-------------------------SS---DASTGNG--
HvCPK14         MGMCCR-------WFRKSHP-----------------------------AASGSSRPS--
OsCPK14         MGNCCP-------PGSSSEPD-------------------------PPPASSGSSRPAGS
AtCPK17         MGNCCS-------HGRDSADNG-------------------DALENGASASNAANSTGPT
AtCPK34         MGNCCS-------HGRDSDDN-----------------------KEEPRPENGGGGVG-A
OsCPK25         MGQCCT-GGGKAVAGDEAEPGTSKA----------------APPSRGTSSKNGSAKQQPC
OsCPK26         MGQCCT-GGGKAVAGDEAEPGTSKA----------------APPSRGTSSKNGSAKQQPC
HvCPK25         MGQCCTNGAGQAAAADAAAEAEPPA----------------PPKTPRGDNAPTNDGLAPG
HvCPK12         MGNCFTK------TYEIP----ITSGTFDRP------PPSFGPQQPAAG--HGTGKPPRP
OsCPK12         MGNCFTK------TYEIP----ITSGTMRRP------ASTAERSKARGG--DEPG-----
AtCPK29         MLQNQHK------TTKNQRNKNIGTKYFLRKKIMGFCFSKFGKSQTHEIPISSSSDSSPP
HvCPK3          MGQCCSR------ATS--------------------------PDSVQGG----ANGYGYS
OsCPK19         MGSCCSR------ATS--------------------------PDSGRGG----ANGYGYS
AtCPK9          MGNCFAK------NHG-L------------------------MKPQQNGNTTRSVEVGVT
AtCPK33         MGNCLAK------KYGLV------------------------MKPQQNG--ERSVEIENR
AtCPK21         MGCFSS-------KHRKT------------------------QN---DGG-EKSIPINPV
AtCPK23         MGCFSS-------KHRKT------------------------QN---DGGGERSIPIIPV
AtCPK15         MGCFSS-------KHRNT------------------------ESDIINGSVQSSIPTNQP
AtCPK19         MGCLCIN------LKKKV------------------------KKPTPDISGEQNTEVKSR
AtCPK27         MG-CFSS------KELQQ------------------------S-----------------
AtCPK31         MG-CYSS------KNLKQ------------------------S-----------------
AtCPK22         MGNCCGS------KPLTA------------------------SDIVSDQ-----------
HvCPK6          MGN-CCGAPSTQG---------------------------GGERKNNRRK----------
OsCPK8          MGN-CCGTPATAE--------------------------EGGKRRRRGKQ----------
HvCPK7          MGN-CCATPPAVD--------------------------GGGAGKQQQQQ---HKEPKQ-
OsCPK20         MGN-CCVTPEGS---------------------------GRGRKKQQQEQKQKQKEPKQQ
AtCPK7          MGN-CCGN-PSSA--------------------------TNQSKQGKPKN----------
AtCPK8          MGN-CCAS-PGSE--------------------------TG-SKKGKPKI----------
AtCPK14         MGN-CCGT-AGSL--------------------------IQD--KQKKGF----------
```

```
AtCPK32         MGN-CCGT-AGSL--------------------------AQNDNKPKKGR----------
OsCPK3          MGN-CCRSPAAAA--------------------------REDVKSSHFPASAG-------
OsCPK16         MGN-CCRSPAAAA--------------------------REDVKTSHFPASTGGG-----
HvCPK10         MGN-CCRSPAAAA--------------------------REDVKSSHFPASAAAAAAKK-
AtCPK13         MGN-CCRSPAAVA--------------------------REDVKSNYS------------
AtCPK10         MGN--CNACVRPD--------------------------SKESKP---------------
AtCPK30         MGN--CIACVKFD--------------------------PDNSKP---------------
OsCPK9          MGNTCCVAPATTD--------------------------EVGAPPRDHHHAAKKSPAPSA
HvCPK29         MGN-CCVARPS------F--------------------KRRGGGS---PRQRGGR--LGG
OsCPK29         MGN-CCVSRPSG----AD--------------------KRRRCGSSTAPHTRGGRRVIGA
HvCPK21         MGG-CYSAYACSRKLR----------------------GRLGNLSFVLPVTERDAAAANA
OsCPK21         MGG-CYSAYASSRKLR----------------------GRISKISLVIPDPVPDAEAA--
HvCPK22         MGG-CYSVIAAS-RML----------------------ARRRAAAAIMPVAGADD----C
OsCPK22         MGG-CSSAFAVSTRMIRF--------------------SRGRVPAAILPVTSNDEPCCSC
AtCPK24         MGS-CVSSPLKG--------------------------SPFGKRPVRRRHSSNSR-----
HvCPK5          MGACLSSSKQEARR-----------------------------RRPRG------------
OsCPK4          MGACFSSHTATAAADGGS-----------------------GKRQQRK------------
HvCPK11         MGLCTSSSA----------------------------------ASPVR------------
OsCPK18         MGLCSSSSAR-------R-----------------------DAGTPGG------------
AtCPK16         MGLCFSSAAKSSGHNRSS-----------------------RNPHPHPPLTVVKSRPPRS
AtCPK18         MGLCFS-SPKATRRGTGS-----------------------RNPNPDSPTQGKAS-----
AtCPK28         MGVCFS-AIRVTGASSSR-----------------------RSSQTKS------------


HvCPK6a         ----------------------------HQHQLS--------------------------
OsCPK6          --------------STATSSGGRMPIRSHQQRLS--------------------------
HvCPK4          --------------AKTDGHAAPA-QPAAAMRRGG-------------------------
OsCPK13         --------------AKTDDHPVSASAPAAAMRRG--------------------------
HvCPK5a         ---------------PAAVADAAAPPPAAGMRRG--------------------------
OsCPK5          --------------APRGSAAAADAPHAASMKRG--------------------------
AtCPK5          --------------NNSKDPALVIPLREPIMRRN--------------------------
AtCPK6          -------------------PALVIPVKEPIMRRN--------------------------
AtCPK26         ------------------------PQEEATMKHS--------------------------
OsCPK7          --------------DTWPEVNNFKPTAASILRRG--------------------------
OsCPK23         --------------DCYSGSS--RASLAGALRQG--------------------------
HvCPK2          ---------------MQPDASGNAAGGGGANPRP--------------------------
HvCPK24         ---------------MQPDASGNAAGGGGANPRP--------------------------
OsCPK24         ---------------MQPDPSG-SGGDGNANAKA--------------------------
HvCPK28         ---------------MQPDPK--GPGREKAH-----------------------------
OsCPK28         ---------------MQPDPQPHGRGREKAAGAG--------------------------
AtCPK4          ---------------ME-KPNPRRPSN---------------------------------
AtCPK11         ---------------METKPNPRRPSN---------------------------------
AtCPK12         ---------------MANKPRT--------------------------------------
HvCPK17         ---------------SICSSTSSAAPDLETIDDS--------------------------
OsCPK17         ---------------SHSSVTSSTAPERVTIADS--------------------------
HvCPK11a        ------------------------------------------------------------
OsCPK11         -----------EPVTIPPSEHSSHHSSRSTDPST--------------------------
HvCPK8          DVAVHIVSKAPEPVKIAPGTANTKPDEAAKAKAKPPAAAPK-------------------
OsCPK10         ----------PPPTSDAPLAVQNKPPEHVKIVST--------------------------
OsCPK27         P---------PVPVVISEPPPPQPQPEPQPAAPS--------------------------
AtCPK20         D--------SNGHVSSTVDPAPSTLPTPSTPPPP--------------------------
AtCPK1          R-------------SRLSDEVQNKPPEQVTMPKPGTDVETK---DREIRTESK-------
AtCPK2          --------------EPATDQVQNKPPEPITMPSSKTNPETKLKPDLEIQPEEKKEKVLAE
AtCPK25         D--------------PKLLEGKEKPAQKQTSQGQ--------------------------
HvCPK15         HRRQPQPPPPQPP-PAPAPPPQPQPALPP-------------------------------
OsCPK15         PKPKPQPPPPQQPRSQPPPPPRHQPQQAPQQA----------------------------
HvCPK1          PKPKPQ----------TAPAPAPTPEAGQV------------------------------
OsCPK1          QWPRPQQ---------PTPPPAAAPDA---------------------------------
AtCPK3          GERRGSSGSGTVGSSGSGTGGSRSTTSTQ-------------------------------
HvCPK2a         -GASIKAGG-------ASPSSAPAQNKPP-------------------------------
OsCPK2          -SSSFKAG--------ASPSSAPAQNKPP-------------------------------
HvCPK14         DGSSGATSS-------ALPSTAPQPVKPL-------------------------------
OsCPK14         AGAAASPAT-------ISPSAAPAPAKPP-------------------------------
AtCPK17         AEASVPQSK-------HAPPSPPPATKQ--------------------------------
AtCPK34         AEASVRASK-------HPPASPPPATKQ--------------------------------
OsCPK25         SPAAKAAAT-------EAAAAASSSKKPA-------------------------------
OsCPK26         SPAAKAAAT-------EAAAAASSSKKPA-------------------------------
```

```
HvCPK25         PGAAEAEPA-------SPATKADPNAKPP-------------------------------
HvCPK12         PSTASWR-PTLPKQQQPPPPPRPSGRPPLPGF----------------------------
OsCPK12         ----TWRRPSFPRHGAPPHRP-PTGSSSAAGA----------------------------
AtCPK29         HHYQPLPKPTVSQGQTSNPTSNPQPKPKPAPP----------------------------
HvCPK3          HQPKQAQTPPSYNNAQPPPQAEVRYTPPAMNP----------------------------
OsCPK19         HQTKPAQTTPSYNHPQPPPPAEVRYTPSAMNP----------------------------
AtCPK9          NQDPPSYTPQARTTQQPEKPGSVNSQPPPWRA----------------------------
AtCPK33         RRS---------THQDPSKISTGTNQPPPWRN----------------------------
AtCPK21         QTHVV---PEHRKP---QTPTPKPMTQPIHQQ----------------------------
AtCPK23         QTHIVDQVPDHRKP---QIPSP--------------------------------------
AtCPK15         ENHVSRDVLKPQKPPSPQIPTTTQSNHHHQQESKPVNQQIEKK-----------------
AtCPK19         EITPKEQ-PRQRQP----APRAKFQIVVQPHKLPLPLPQPQEK-----------------
AtCPK27         ------------------------------------------------------------
AtCPK31         ------------------------------------------------------------
AtCPK22         ------------------------------------------------------------
HvCPK6          --------------------AKANPYTVAY------------------------------
OsCPK8          --------------------KKANPFTVAY------------------------------
HvCPK7          -----------------RKGKKPNPFSIEY------------------------------
OsCPK20         QQ-------------QQKKGKKPNPFSIEY------------------------------
AtCPK7          --------------------KNNPFYSNEY------------------------------
AtCPK8          --------------------KSNPFYSEAY------------------------------
AtCPK14         --------------------KLPNPFSNEY------------------------------
AtCPK32         --------------------KKQNPFSIDY------------------------------
OsCPK3          -------------------KKKPHQARNGGVGGGGG------------------------
OsCPK16         -------------------KKKPHQARNG------G------------------------
HvCPK10         ------------------KKNKPHQPRSG-------------------------------
AtCPK13         ---------------------GHDHARKD-------------------------------
AtCPK10         ----------------SSKPKKPNRDRKL-NPFAG-------------------------
AtCPK30         ----------------NQK-KKPPRGRQR-NPYDDP------------------------
OsCPK9          TTTTATRQRHG----QEPKPKPKPRARAKPNPYDWA------------------------
HvCPK29         AN----------------LRCLSTVSSVTDT-----------------------------
OsCPK29         AN----------------MRCLSTVSSVSDA-----------------------------
HvCPK21         AAET--------------SSTSSAKKGDHGS-----------------------------
OsCPK21         ------------------SPRKDGVDGDGDD-----------------------------
HvCPK22         PPADDAANS--------GSASKKKRRSTKWR-----------------------------
OsCPK22         SPENNNKNNDGGGGGCDGGEHQKGKSWRRWQ-----------------------------
AtCPK24         ------------------TSSVPRFDSSTNL-----------------------------
HvCPK5          ------------------GKAEEKAAAAG-------------------------------
OsCPK4          ------------------GDHKGKLPDGGGGE----------------------------
HvCPK11         ------------------NKEKGWKGSGGGGG----------------------------
OsCPK18         ------------------GNGAGNKDNAGRKG----------------------------
AtCPK16         PCSFMAVTIQKDHRTQPRRNATAKKTPTRHTPPHGKVREKVIS-----------------
AtCPK18         ------------EKVSNKNKKNTKKIQLRHQG----------------------------
AtCPK28         -------------KAAP-TPIDTKASTKRRTG----------------------------


HvCPK6a         ------------------------------------------------------------
OsCPK6          ------------------------------------------------------------
HvCPK4          ------------------------------------------------------------
OsCPK13         ------------------------------------------------------------
HvCPK5a         ------------------------------------------------------------
OsCPK5          ------------------------------------------------------------
AtCPK5          ------------------------------------------------------------
AtCPK6          ------------------------------------------------------------
AtCPK26         ------------------------------------------------------------
OsCPK7          ------------------------------------------------------------
OsCPK23         ------------------------------------------------------------
HvCPK2          ------------------------------------------------------------
HvCPK24         ------------------------------------------------------------
OsCPK24         ------------------------------------------------------------
HvCPK28         ------------------------------------------------------------
OsCPK28         ------------------------------------------------------------
AtCPK4          ------------------------------------------------------------
AtCPK11         ------------------------------------------------------------
AtCPK12         ------------------------------------------------------------
HvCPK17         ---------------------------------------------DLSSPSSNP------
OsCPK17         ---------------------------------------------DLSSSTPNKG-----
```

```
HvCPK11a        -------------------------------------------------MAPVAK-----
OsCPK11         ---------------------------------------------PTSAAEQPAN-----
HvCPK8          ------------------------------PAAAAAAKEQDPKGSTDSSSSSGTGEAGAP
OsCPK10         --------------------------------TDTASAEQD---ASKSSAGSDSGEAARP
OsCPK27         --------------------------------QPPPPQEQPSPPPPASSNTTQQPPPPQQ
AtCPK20         --------------------------------VKMANEEPPPKPITENKEDPNSKP----
AtCPK1          --------PETLEEISLE------------SKPETKQETKS-----ETKPESKPDPPAKP
AtCPK2          ETKQKVVPEESKQEVPPEESKREVVVQPESAKPETKSESKPETTKPETTSETKPETKAEP
AtCPK25         ----------------------------------GGRKCSDEEYKKRAIACANSK-----
HvCPK15         ------------------------------------------------------------
OsCPK15         ------------------------------------------------------------
HvCPK1          ------------------------------------------------------------
OsCPK1          ------------------------------------------------------------
AtCPK3          ------------------------------------------------------------
HvCPK2a         ------------------------------------------------------------
OsCPK2          ------------------------------------------------------------
HvCPK14         ------------------------------------------------------------
OsCPK14         ------------------------------------------------------------
AtCPK17         ------------------------------------------------------------
AtCPK34         ------------------------------------------------------------
OsCPK25         ------------------------------------------------------------
OsCPK26         ------------------------------------------------------------
HvCPK25         ------------------------------------------------------------
HvCPK12         ------------------------------------------------------------
OsCPK12         ------------------------------------------------------------
AtCPK29         ------------------------------------------------------------
HvCPK3          ------------------------------------------------------------
OsCPK19         ------------------------------------------------------------
AtCPK9          ------------------------------------------------------------
AtCPK33         ------------------------------------------------------------
AtCPK21         ------------------------------------------------------------
AtCPK23         ------------------------------------------------------------
AtCPK15         ------------------------------------------------------------
AtCPK19         ------------------------------------------------------------
AtCPK27         ------------------------------------------------------------
AtCPK31         ------------------------------------------------------------
AtCPK22         ------------------------------------------------------------
HvCPK6          ------------------------------------------------------------
OsCPK8          ------------------------------------------------------------
HvCPK7          ------------------------------------------------------------
OsCPK20         ------------------------------------------------------------
AtCPK7          ------------------------------------------------------------
AtCPK8          ------------------------------------------------------------
AtCPK14         ------------------------------------------------------------
AtCPK32         ------------------------------------------------------------
OsCPK3          ------------------------------------------------------------
OsCPK16         ------------------------------------------------------------
HvCPK10         ------------------------------------------------------------
AtCPK13         ------------------------------------------------------------
AtCPK10         ------------------------------------------------------------
AtCPK30         ------------------------------------------------------------
OsCPK9          ------------------------------------------------------------
HvCPK29         ------------------------------------------------------------
OsCPK29         ------------------------------------------------------------
HvCPK21         ------------------------------------------------------------
OsCPK21         ------------------------------------------------------------
HvCPK22         ------------------------------------------------------------
OsCPK22         ------------------------------------------------------------
AtCPK24         ------------------------------------------------------------
HvCPK5          ------------------------------------------------------------
OsCPK4          ------------------------------------------------------------
HvCPK11         ------------------------------------------------------------
OsCPK18         ------------------------------------------------------------
AtCPK16         ------------------------------------------------------------
AtCPK18         ------------------------------------------------------------
AtCPK28         ------------------------------------------------------------
```

KINASE DOMAIN

```
HvCPK6a     --------------------SPTAVLGHHT-----PPLRDLYSLGRKLGQGQFGTTYLCT
OsCPK6      --------------------SPTAVLGHET-----PALREVYTVGRKLGQGQFGTTYLCT
HvCPK4      --------------AGAPADLGS-VLGHPT-----PNLRDLYALGRKLGQGQFGTTYLCT
OsCPK13     ---------------QAPADLGS-VLGHPT-----PNLRDLYAMGRKLGQGQFGTTYLCT
HvCPK5a     --------------SLAPAELTANVLGHPT-----PSLHDHYLLGRKLGQGQFGTTYLCT
OsCPK5      --------------APAPAELTANVLGHPT-----PSLSEHYALGRKLGQGQFGTTYLCT
AtCPK5      -----------------PDNQAYYVLGHKT-----PNIRDIYTLSRKLGQGQFGTTYLCT
AtCPK6      -----------------VDNQSYYVLGHKT-----PNIRDLYTLSRKLGQGQFGTTYLCT
AtCPK26     -----------------GGNQACYVLGQKT-----PSIRDLYSLGHKLGQGQFGTTYMCK
OsCPK7      -----------------LDPTSINVLGRKT-----ADLREHYIIGRKLGQGQFGTTYLCT
OsCPK23     -----------------LNLKSP-VLGYKT-----PNVRELYTLGRELGQGQFGKTYLCT
HvCPK2      ------KLPPPVAAAPAPSGRPASVLPHKT-----ANVRDHYRIGKKLGQGQFGTTYLCV
HvCPK24     ------KLPPPVAAAPAPSGRPASVLPHKT-----ANVRDHYRIGKKLGQGQFGTTYLCV
OsCPK24     ------KLAPPPVTAAG--GRPVSVLPHKT-----ANVRDHYRIGKKLGQGQFGTTYLCV
HvCPK28     ------VRLPPPVTAPS-VGRPASVLPHKT-----ANVRDHYRIGKKLGQGQFGTTYLCV
OsCPK28     ------PRLPPPVTAPS-VGRPASVLPHKT-----ANVRDHYRIGKKLGQGQFGTTYLCV
AtCPK4      -----------------------SVLPYET-----PRLRDHYLLGKKLGQGQFGTTYLCT
AtCPK11     -----------------------TVLPYQT-----PRLRDHYLLGKKLGQGQFGTTYLCT
AtCPK12     ----------------------RWVLPYKT-----KNVEDNYFLGQVLGQGQFGTTFLCT
HvCPK17     -------NKPKVKRVQSAGLLAESVLKRDS-----ERIKDLYTLGKKLGQGQFGTTYKCV
OsCPK17     ------GNKPKVRRVQSAGLLADSVLKRDS-----ERLKDLYTLGKKLGQGQFGTTYQCV
HvCPK11a    ------KPVPKVKRVQSAGLLADSVLKRDVNT---ARLKDLYTIGKKLGQGQFGTTYLCV
OsCPK11     ------KAAPKVKRVQSAGLLADSVLKRDVNT---ARLKDLYTIGKKLGQGQFGTTYLCV
HvCPK8      KPPHHRPKVPPVKRVSSAGLLVGSVLKRKT-----ESVKEKYSLGRRLGQGQFGTTYLCV
OsCPK10     -----RPRVPPVKRVSSAGLLVGSVLKRKT-----ESLKDKYSLGRKLGQGQFGTTYLCV
OsCPK27     RQQSRAKKPAHIKRISSAGLQVESVLRRKT-----ENLKDKYSLGRKLGQGQFGTTYLCV
AtCPK20     -----QKKEAHMKRMASAGLQIDSVLGRKT-----ENLKDIYSVGRKLGQGQFGTTFLCV
AtCPK1      ------KKPKHMKRVSSAGLRTESVLQRKT-----ENFKEFYSLGRKLGQGQFGTTFLCV
AtCPK2      ------QKPKHMRRVSSAGLRTESVLQRKT-----ENFKEFYSLGRKLGQGQFGTTFLCL
AtCPK25     ------RKAHNVRRLMSAGLQAESVLKTKT-----GHLKEYYNLGSKLGHGQFGTTFVCV
HvCPK15     --------------------DVGRVLGRPM-----EDVRATYTFGRELGRGQFGVTYLAT
OsCPK15     ----------------AAEDGVGRVLGRPM-----EDVRATYTFGRELGRGQFGVTYLAT
HvCPK1      --------------------AMGRVLGRPM-----EDVRATYTFGRELGRGQFGVTYLVT
OsCPK1      --------------------AMGRVLGRPM-----EDVRATYTFGRELGRGQFGVTYLVT
AtCPK3      --------------------QNGRILGRPM-----EEVRRTYEFGRELGRGQFGVTYLVT
HvCPK2a     -------------------AAIGPVLGRPM-----EDVRSIYTVGKELGRGQFGVTSLCT
OsCPK2      -------------------APIGPVLGRPM-----EDVRSIYTIGKELGRGQFGVTSLCT
HvCPK14     -------------------APIGPVLGRPM-----EDVKSIYNVGKELGRGQFGVTSLCT
OsCPK14     -------------------APIGPVLGRPM-----EDVKSIYTVGKELGRGQFGVTSLCT
AtCPK17     -------------------GPIGPVLGRPM-----EDVKASYSLGKELGRGQFGVTHLCT
AtCPK34     -------------------GPIGPVLGRPM-----EDVKSSYTLGKELGRGQFGVTHLCT
OsCPK25     -------------------GPIGEVLERPM-----EEVRTTYSIGKELGRGQFGVTHLCT
OsCPK26     -------------------GPIGEVLERPM-----EEVRTTYSIGKELGRGQFGVTHLCT
HvCPK25     -------------------GPVGEVLGRPI-----EDVRATYTIGEELGRGQFGVTYLCT
HvCPK12     --------LSGSLSRKVPVGEIGPVLQRPM-----ADVRALYNLERKLGSGQFGTTYLCT
OsCPK12     --------LSRRASGGG--GEMGPVLQRAM-----VSVRSLYQLDRKLGSGQFGTTYLCT
AtCPK29     --------PPPSTSSGS---QIGPILNRPM-----IDLSALYDLHKELGRGQFGITYKCT
HvCPK3      -------PVVPPVVAP-SKPTPDTILGKQY-----EDVRSVYSLGKELGRGQFGVTYLCT
OsCPK19     -------PVVPPVVAP-PKPTPDTILGKPY-----DDVRSVYSLGKELGRGQFGVTYLCT
AtCPK9      -------AAAAPGLSPKTTTKSNSILENAF-----EDVKLFYTLGKELGRGQFGVTYLCT
AtCPK33     -------PAKHSG--------AAAILEKPY-----EDVKLFYTLSKELGRGQFGVTYLCT
AtCPK21     --------ISTPSSNPVSVRDPDTILGKPF-----EDIRKFYSLGKELGRGQFGITYMCK
AtCPK23     -------------SIPISVRDPETILGKPF-----EDIRKFYSLGRELGRGGLGITYMCK
AtCPK15     -------HVLTQPLKPIVFRETETILGKPF-----EEIRKLYTLGKELGRGQFGITYTCK
AtCPK19     -------QKLINHQKQSTLQQPEPILGRPF-----EDIKEKYSLGRELGRGQFGITYICT
AtCPK27     ---------------------KRTILEKPL-----VDITKIYILGEELGRGNFGLTRKCV
AtCPK31     ---------------------KRTILEKPF-----VDIGKVYILGDELGQGQFGITRKCV
AtCPK22     --------------------KQETILGKPL-----EDIKKHYSFGDELGKG---------
HvCPK6      -------NRG---AAGPPARAGLVVLRDPTG----RDLDDKYVLGGELGRGEFGVTYLCT
OsCPK8      -------NRAPSSAGAAAGRPGLMVLRDPTG----RDLGARYELGGELGRGEFGITYLCT
HvCPK7      -------NRSAPPGAS-----RLVVLRDPTGT--GRDIAERYELGGELGRGEFGVTYLCT
OsCPK20     -------NRSSAPSGH-----RLVVLREPTG----RDIAARYELGGELGRGEFGVTYLCT
AtCPK7      -------ATTDRSGAG----FKLSVLKDPTG----HDISLQYDLGREVGRGEFGITYLCT
AtCPK8      -------TTNG-SGTG----FKLSVLKDPTG----HDISLMYDLGREVGRGEFGITYLCT
```

```
AtCPK14         -------GNHHDG-------LKLIVLKEPTG----HEIKQKYKLGRELGRGEFGVTYLCT
AtCPK32         -------GLHHGGGDGGGRPLKLIVLNDPTG----REIESKYTLGRELGRGEFGVTYLCT
OsCPK3          -------GGGGGGGGGGAGQKRLPVLGEEGCEL-IGGIDDKYALDRELGRGEFGVTYLCM
OsCPK16         -------GGGGGGGGGGWEKKRLSVLGEEGSEV-NGGIEEKYALDRELGRGEFGVTYLCM
HvCPK10         --------TAGGNGGGGGGQKRLAVLGEEGCDF-IGGIDDKYLLDRELGRGEFGVTYLCV
AtCPK13         -------------AAGGKKSAPIRVLSDVP----KENIEDRYLLDRELGRGEFGVTYLCI
AtCPK10         --------------DFTRSPAPIRVLKDVIPMSNQTQISDKYILGRELGRGEFGITYLCT
AtCPK30         --------------DGLRTHAPLR----VIPMSHQSQISDKYILGRELGRGEFGITYLCT
OsCPK9          -------PPRVLPARGGAAASAVRVLEGVVPHHPRLRVTDKYQLGRELGRGEFGVTHLAT
HvCPK29         -------PRATAQPWSSVTVLGKGLAASDN----IEELLRRYQLGEELGRGEFGVTRRCM
OsCPK29         -------ARAVMS-NEPATVLGNSGSSGNGGVMAAEEMLRRYEIGEELGRGEFGVTRRCR
HvCPK21         -------SRRSNGEGPTGGTEEEELVTKTT----TAEFGRRYVLGKELGRGEFGVTRRCR
OsCPK21         -------VRGGGGGCDDGG--DVVAIATTT----ADEFARRYVLGKELGRGEFGVTRRCS
HvCPK22         -------RSAPILGGGGGGDDDQCVPGAGG-----ECFAKRYRLGAELGRGEFGVTRRCE
OsCPK22         -------YRRCGGGGGGGGGRKNAILGDAADVKTAAGFAERYRLGAELGRGEFGVTRRCS
AtCPK24         -------SRRLIFQPPSRVLPEPIG----------DGIHLKYDLGKELGRGEFGVTHECI
HvCPK5          --------------------APAVEFGYDR------DFDGRYEVGRLLGHGQFGYTFAAV
OsCPK4          ----------------KEKEAARVEFGYER------DFEGRYQVGRLLGHGQFGYTFAAT
HvCPK11         ----------------IVACGKRTDFGYDK------DFEARYALGKLLGHGQFGYTFAAV
OsCPK18         ----------------IVACGKRTDFGYDK------DFEARYALGKLLGHGQFGYTFAAV
AtCPK16         --------NNGRRHGETIPYGKRVDFGYAK------DFDHRYTIGKLLGHGQFGYTYVAT
AtCPK18         ----------------GIPYGKRIDFGYAK------DFDNRYTIGKLLGHGQFGFTYVAT
AtCPK28         ----------------SIPCGKRTDFGYSK------DFHDHYTIGKLLGHGQFGYTYVAI
                                                     .   * .   :* *
```

KINASE DOMAIN

```
HvCPK6a         ETATGTAFACKSIAKRKLL--------------------TPEDVEDVRREIQIMHHLA--
OsCPK6          QVSTGAEYACKSIAKRKLL--------------------SPEDVEDVRREIQIMHHLA--
HvCPK4          ELATGADYACKSISKRKLI--------------------TKEDIDDVRREIQIMHHLS--
OsCPK13         ELSTGVDYACKSISKRKLI--------------------TKEDIEDVRREIQIMHHLS--
HvCPK5a         DRATGADYACKSIGKRKLI--------------------TKEDVEDVRREIQIMHHLA--
OsCPK5          DLATGVDYACKSIAKRKLI--------------------TKEDVEDVRREIQIMHHLA--
AtCPK5          EIASGVDYACKSISKRKLI--------------------SKEDVEDVRREIQIMHHLA--
AtCPK6          DIATGVDYACKSISKRKLI--------------------SKEDVEDVRREIQIMHHLA--
AtCPK26         EISTGREYACKSITKRKLI--------------------SKEDVEDVRREIQIMHHLA--
OsCPK7          EINTGCEYACKTIPKRKLI--------------------TKEDVEDVRREIQIMHHLS--
OsCPK23         EISTGCQYACKTILKSNLR--------------------CVSDIEDVRREIQIMHHLS--
HvCPK2          AKEDGGEFACKSIPKRKLL--------------------CREDYEDVWREIQIMHHPS--
HvCPK24         AKEDGGEFACKSIPKRKLL--------------------CREDYEDVWREIQIMHHLS--
OsCPK24         DKASGGEFACKSIPKRKLL--------------------CREDYEDVWREIQIMHHLS--
HvCPK28         AKEDGGEYACKSIPKRKLL--------------------CREDYEDVWREIQIMHHLS--
OsCPK28         GKPDGGEYACKSIPKRKLL--------------------CREDYEDVWREIQIMHHLS--
AtCPK4          EKSSSANYACKSIPKRKLV--------------------CREDYEDVWREIQIMHHLS--
AtCPK11         EKSTSANYACKSIPKRKLV--------------------CREDYEDVWREIQIMHHLS--
AtCPK12         HKQTGQKLACKSIPKRKLL--------------------CQEDYDDVLREIQIMHHLS--
HvCPK17         EKATGKEFACKSIAKRKLV--------------------TEEDVEDVRREIQIMHHLA--
OsCPK17         EKATGKVLACKSIAKRKLV--------------------SEEDVEDVRREIQIMHHLA--
HvCPK11a        EKATGKEYACKSIAKRKLL--------------------TDEDVEDVRREIQIMHHLA--
OsCPK11         EKATGREFACKSIAKRKLL--------------------TQEDVEDVRREIQIMHHLA--
HvCPK8          ERSSGKEYACKSILKRKLV--------------------TDDDVEDVRREIQIMYHLA--
OsCPK10         ERATGKEFACKSILKRKLV--------------------TDDDVEDVRREIQIMYHLA--
OsCPK27         DKANGGEYACKSIAKRKLL--------------------TDEDVEDVRREIQIMHHLA--
AtCPK20         DKKTGKEFACKTIAKRKLT--------------------TPEDVEDVRREIQIMHHLS--
AtCPK1          EKTTGKEFACKSIAKRKLL--------------------TDEDVEDVRREIQIMHHLA--
AtCPK2          EKGTGNEYACKSISKRKLL--------------------TDEDVEDVRREIQIMHHLA--
AtCPK25         EKGTGEEYACKSIPKRKLE--------------------NEEDVEDVRREIEIMKHLL--
HvCPK15         HKSTGARYACKSIAARKLA--------------------RADDVEDARREVQIMHHLT--
OsCPK15         HKPTGRRYACKSIAARKLA--------------------RPDDLDDVRREVHIMHHLT--
HvCPK1          HKATGQRFACKSIATRKLV--------------------HRDDIEDVQREVQIMHHLT--
OsCPK1          HKATGKRFACKSIATRKLA--------------------HRDDIEDVRREVQIMHHLT--
AtCPK3          HKETKQQVACKSIPTRRLV--------------------HKDDIEDVRREVQIMHHLS--
HvCPK2a         HKATGQKFACKTIAKRKLS--------------------TKEDVEDVRREVQIMYHLA--
OsCPK2          HKATGQKFACKTIAKRKLS--------------------TKEDVEDVRREVQIMYHLA--
HvCPK14         QKATGQKLACKTISKRKLS--------------------TKEDVEDVRREVQIMYHLA--
OsCPK14         HKATGQRFACKTISKRKLS--------------------TKEDVEDVRREVQIMYHLA--
AtCPK17         QKATGHQFACKTIAKRKLV--------------------NKEDIEDVRREVQIMHHLT--
```

```
AtCPK34         QKATGLQFACKTIAKRKLV--------------------NKEDIEDVRREVQIMHHLT--
OsCPK25         HKATGEKLACKTIAKRKLA--------------------NKEDVDDVRREVQIMHHLS--
OsCPK26         HKATGEKLACKTIAKRKLA--------------------NKEDVDDVRREVQIMHHLS--
HvCPK25         HTETGEKLACKTIAKRKLS--------------------GAEDVEDVRREVEIMHHLS--
HvCPK12         ERATGLKYACKSVSKRKLV--------------------RRADVEDMRREVTILQHLS--
OsCPK12         ERATGNRYACKSVSKRKLV--------------------RRTDVDDVRREITILQHLS--
AtCPK29         DKSNGREYACKSISKRKLI--------------------RRKDIEDVRREVMILQHLT--
HvCPK3          EISTGRQYACKSISKRKLV--------------------SKADKEDIRREIQIMQHLS--
OsCPK19         EIASGKQYACKSISKRKLV--------------------SKADKEDIRREIQIMQHLS--
AtCPK9          ENSTGKKYACKSISKKKLV--------------------TKADKDDMRREIQIMQHLS--
AtCPK33         EKSTGKRFACKSISKKKLV--------------------TKGDKEDMRREIQIMQHLS--
AtCPK21         EIGTGNTYACKSILKRKLI--------------------SKQDKEDVKREIQIMQYLS--
AtCPK23         EIGTGNIYACKSILKRKLI--------------------SELGREDVKTEIQIMQHLS--
AtCPK15         ENSTGNTYACKSILKRKLT--------------------RKQDIDDVKREIQIMQYLS--
AtCPK19         EISSGKNFACKSILKRKLI--------------------RTKDREDVRREIQIMHYLS--
AtCPK27         EKSTGKTFACKTILKTKLK--------------------DEECEEDVKREIRIMKQLS--
AtCPK31         EKTSGKTYACKTILKTNLK--------------------SREDEEAVKREIRIMKHLS--
AtCPK22         -----KSYACKSIPKRTLS--------------------SEEEKEAVKTEIQIMDHVS--
HvCPK6          EAATGARMACKSISKRKL--------------------RTPVDVEDVRREVEIMRHMP--
OsCPK8          EAETGDRYACKSISKRKL--------------------RTPVDVEDVRREVEIMRHMP--
HvCPK7          DRDTREALACKSISKKKL--------------------RTAVDIEDVRREVEIMRHLP--
OsCPK20         ERETGDAYACKSISKKKL--------------------RTAVDIEDVRREVDIMRHLP--
AtCPK7          DKETGEKYACKSISKKKL--------------------RTAVDIEDVRREVEIMKHMP--
AtCPK8          DIKTGEKYACKSISKKKL--------------------RTAVDIEDVRREVEIMKHMP--
AtCPK14         EIETGEIFACKSILKKKL--------------------KTSIDIEDVKREVEIMRQMP--
AtCPK32         DKETDDVFACKSILKKKL--------------------RTAVDIEDVRREVEIMRHMP--
OsCPK3          DRDTKELLACKSISKRKL--------------------RTAVDVEDVRREVAIMRHLP--
OsCPK16         DRCSRELLACKSISKRKL--------------------RTPVDVEDVRREVAIMRHLP--
HvCPK10         DRDTREQLACKSISKRKL--------------------RTPVDVEDVRREVAIMRHLP--
AtCPK13         ERSSRDLLACKSISKRKL--------------------RTAVDIEDVKREVAIMKHLP--
AtCPK10         DRETHEALACKSISKRKL--------------------RTAVDIEDVRREVAIMSTLP--
AtCPK30         DRETREALACKSISKRKL--------------------RTAVDVEDVRREVTIMSTLP--
OsCPK9          DRATRERLACKSIPKRRL--------------------RTAVDVADVRREVAIMASLP--
HvCPK29         DAATGEKLACKSISKRKL--------------------RSSVDIEDVRREVAIMRSLP--
OsCPK29         DAVTGERLACKSISKRKL--------------------RSSVDVEDVRREVAIMRSLP--
HvCPK21         DAATGEALACKTIRRHRRRGGRGLNRKPAGGGGAEAAARAAAAAADVRREVAIMRRMS--
OsCPK21         DAATGEALACKTIRKHRR----------LAPPRVTAAKAAAAHGEDVKREVAIMRRMSSA
HvCPK22         DAATGEALACKTIRRKRL-------------------RRA-ADAEDVRREVEILRRMS--
OsCPK22         DAATGEALACKTIRRKRL-------------------RRCRGDAEDVRREVEILRRIS--
AtCPK24         EISTRERFACKRISKEKL--------------------RTEIDVEDVRREVEIMRCLP--
HvCPK5          ERLSGDRVAVKRIDKAKMT--------------------RPVAVEDVKREVKILKALK--
OsCPK4          DRASGDRVAVKRIDKAKMV--------------------RPVAVEDVKREVKILKELK--
HvCPK11         NRHSDESVAVKRIDKNKMV--------------------LPVAVEDVKREVKILKALH--
OsCPK18         DRRSSERVAVKRIDKNKMV--------------------LPVAVEDVKREVKILKALQ--
AtCPK16         DKKTGDRVAVKKIDKAKMT--------------------IPIAVEDVKREVKILQALT--
AtCPK18         DNNNGNRVAVKRIDKAKMT--------------------QPIEVEDVKREVKILQALG--
AtCPK28         HRPNGDRVAVKRLDKSKMV--------------------LPIAVEDVKREVQILIALS--
                        * * :                                    *: *:
```

KINASE DOMAIN

```
HvCPK6a         ---------GHASVVTIKGAYEDA--QYVHIVMELCEGGELFDRIVQRG----YFSERRA
OsCPK6          ---------GHGSVVTIQGAYEDN--LYVHIVMELCEGGELFDRIVERG----YFSERKA
HvCPK4          ---------GHKNVVAIKGAYEDQ--LYVHIVMELCAGGELFDRIIQRG----HYSERKA
OsCPK13         ---------GHKNVVAIKGAYEDQ--LYVHIVMELCAGGELFDRIIQRG----HYSERKA
HvCPK5a         ---------GHRNVVAIKGAYEDP--VYVHIVMELCGGGELFDRIIQRG----HYSERKA
OsCPK5          ---------GHRNVVAIKGAYEDP--QYVHIVMELCAGGELFDRIIERG----QFSERKA
AtCPK5          ---------GHGSIVTIKGAYEDS--LYVHIVMELCAGGELFDRIIQRG----HYSERKA
AtCPK6          ---------GHKNIVTIKGAYEDP--LYVHIVMELCAGGELFDRIIHRG----HYSERKA
AtCPK26         ---------GYKNIVTIKGAYEDP--LYVHIVMELCSGGELFDRIIQRG----HYSERKA
OsCPK7          ---------GHKNVVAIKDVYEDG--QAVHIVMELCAGGELFDRIQEKG----HYSERKA
OsCPK23         ---------GQKNIVTIKDTYEDE--QAVHIVMELCAGGELFSKIQKRG----HYSERKA
HvCPK2          ---------EHPNVVRIRGAYEDA--LFVHIVMELCAGGELFDRIVAKG----HYTERAA
HvCPK24         ---------EHPNVVRIRGAYEDA--LFVHIVMELCAGGELFDRIVAKG----HYTERAA
OsCPK24         ---------EHPNVVRIRGAYEDA--LFVHIVMELCAGGELFDRIVAKG----HYTERAA
HvCPK28         ---------EHPNVVRIRGAYEDA--LFVHLVMELCAGGELFDRIVAKG----HYSERAA
OsCPK28         ---------EHPNVVRIRGAYEDA--LFVHIVMELCAGGELFDRIVAKG----HYTERAA
```

```
AtCPK4          ---------EHPNVVRIKGTYEDS--VFVHIVMEVCEGGELFDRIVSKG----CFSEREA
AtCPK11         ---------EHPNVVRIKGTYEDS--VFVHIVMEVCEGGELFDRIVSKG----HFSEREA
AtCPK12         ---------EYPNVVRIESAYEDT--KNVHLVMELCEGGELFDRIVKRG----HYSEREA
HvCPK17         ---------GHPNVISIVGAYEDA--VAVHLVMELCAGGELFDRIIQRG----HYSEKAA
OsCPK17         ---------GHPSVVSIVGAYEDA--VAVHLVMELCAGGELFDRIVQRG----HYSEKAA
HvCPK11a        ---------GHSSVVSIVGAYEDA--VAVQLVMELCAGGELFDRIIQRG----HYSEKAA
OsCPK11         ---------GHANVVSIVGAYEDA--VAVQLVMELCAGGELFDRIIQRG----HYSEKAA
HvCPK8          ---------GHPNVISIRGAYEDA--VAVHLVMELCAGGELFDRIVQKG----HYTERKA
OsCPK10         ---------GHPNVISIRGAYEDA--VAVHLVMELCAGGELFDRIVQKG----HYTERKA
OsCPK27         ---------GHPNIISIRGAYEDA--VAVHVVMELCAGGELFDRIVRKG----HYTERQA
AtCPK20         ---------GHPNVIQIVGAYEDA--VAVHVVMEICAGGELFDRIIQRG----HYTEKKA
AtCPK1          ---------GHPNVISIKGAYEDV--VAVHLVMECCAGGELFDRIIQRG----HYTERKA
AtCPK2          ---------GHPNVISIKGAYEDV--VAVHLVMELCSGGELFDRIIQRG----HYTERKA
AtCPK25         ---------GQPNVISIKGAYEDS--VAVHMVMELCRGGELFDRIVERG----HYSERKA
HvCPK15         ---------GHRSIVELRGAYEDR--HSVNLVMELCEGGELFDRIIARG----HYSERAA
OsCPK15         ---------GHRNIVELRGAYEDR--HSVNLVMELCEGGELFDRIIARG----HYSERAA
HvCPK1          ---------GHRNIVELRGAYEDR--HSVNLIMELCEGGELFDRIIARG----HYSERAA
OsCPK1          ---------GHRNIVELRGAYEDR--HSVNLIMELCEGGELFDRIIARG----HYSERAA
AtCPK3          ---------GHRNIVDLKGAYEDR--HSVNLIMELCEGGELFDRIISKG----LYSERAA
HvCPK2a         ---------GQPNIVELKGAYEDK--QSVHLVMELCAGGELFDRIITKG----KYTERAA
OsCPK2          ---------GQPNVVELKGAYEDK--QSVHLVMELCAGGELFDRIIAKG----HYTERAA
HvCPK14         ---------GQPGVVELKGAYEDK--HAVHLVMELCAGGELFDRIIAKG----HYTERAA
OsCPK14         ---------GQPGVVELKGAYEDK--HAVHLVMELCAGGELFDRIIAKG----HYTEHAA
AtCPK17         ---------GQPNIVELKGAYEDK--HSVHLVMELCAGGELFDRIIAKG----HYSERAA
AtCPK34         ---------GQPNIVELKGAYEDK--HSVHLVMELCAGGELFDRIIAKG----HYSERAA
OsCPK25         ---------GQPNIVDLRGAYEDK--HNVHLVMELCAGGELFDRIIARG----HYTERAA
OsCPK26         ---------GQPNIVDLRGAYEDK--HNVHLVMELCAGGELFDRIIARG----HYTERAA
HvCPK25         ---------GQPNIVDLRGAYEDK--HNVHLVMELCAGGELFDRIIAKG----HYTERAA
HvCPK12         ---------GQPNIAEFRGAFEDA--ENVHLVMEFCSGGELFDRITAKG----SYSERQA
OsCPK12         ---------GQPNIAEFRGAYEDN--DHVHLVMEFCSGGELFDRITAKG----SYSERQA
AtCPK29         ---------GQPNIVEFRGAYEDK--DNLHLVMELCSGGELFDRIIKKG----SYSEKEA
HvCPK3          ---------GQPNIVEFCGAYEDK--GSVHVVMELCAGGELFDRIIAKG----HYSERAA
OsCPK19         ---------GQQNIVEFRGAYEDK--SNVHVVMELCAGGELFDRIIAKG----HYSERAA
AtCPK9          ---------GQPNIVEFKGAYEDE--KAVNLVMELCAGGELFDRIIAKG----HYTERAA
AtCPK33         ---------GQPNIVEFKGAYEDE--KAVNLVMELCAGGELFDRILAKG----HYSERAA
AtCPK21         ---------GQPNIVEIKGAYEDR--QSIHLVMELCAGGELFDRIIAQG----HYSERAA
AtCPK23         ---------GQPNVVEIKGSYEDR--HSVHLVMELCAGGELFDRIIAQG----HYSERAA
AtCPK15         ---------GQENIVEIKGAYEDR--QSIHLVMELCGGSELFDRIIAQG----HYSEKAA
AtCPK19         ---------GQPNIVEIKGAYEDR--QSVHLVMELCEGGELFDKITKRG----HYSEKAA
AtCPK27         ---------GEPNIVEFKNAYEDK--DSVHIVMEYCGGGELYDKILALYDVGKSYSEKEA
AtCPK31         ---------GEPNIVEFKKAYEDR--DSVHIVMEYCGGGELFKKIEALSKDGKSYSEKEA
AtCPK22         ---------GQPNIVQIKGSYEDN--NSIHIVMELCGGGELFDKIDALVKSHSYYSEKDA
HvCPK6          ---------PHPNIVSLSAAYEDE--DAVHLLMELCEGGELFDRIVARG----HYTERAA
OsCPK8          ---------SHPNIVSLRAAYEDE--DNVHLVMELCEGGELFDRIVARG----HYTERAA
HvCPK7          ---------KHPNIVTLRDTYEDD--NAVHLVMELCEGGELFDRIVARG----HYTERAA
OsCPK20         ---------KHPNIVTLRDTYEDD--NAVHLVMELCEGGELFDRIVARG----HYTERAA
AtCPK7          ---------KHPNVVSLKDSFEDD--DAVHIVMELCEGGELFDRIVARG----HYTERAA
AtCPK8          ---------RHPNIVSLKDAFEDD--DAVHIVMELCEGGELFDRIVARG----HYTERAA
AtCPK14         ---------EHPNIVTLKETYEDD--KAVHLVMELCEGGELFDRIVARG----HYTERAA
AtCPK32         ---------EHPNVVTLKETYEDE--HAVHLVMELCEGGELFDRIVARG----HYTERAA
OsCPK3          ---------KSASIVSLREACEDE--GAVHLVMELCEGGELFDRIVARG----HYTERAA
OsCPK16         ---------RSASIVSLREACEDD--GAVHLVMELCEGGELFDRIVARG----HYTERAA
HvCPK10         ---------RSHSIVALREACEDE--GAVHLVMELCEGGELFDRIVARG----HYTERAA
AtCPK13         ---------KSSSIVTLKEACEDD--NAVHLVMELCEGGELFDRIVARG----HYTERAA
AtCPK10         ---------EHPNVVKLKASYEDN--ENVHLVMELCEGGELFDRIVARG----HYTERAA
AtCPK30         ---------EHPNVVKLKATYEDN--ENVHLVMELCEGGELFDRIVARG----HYTERAA
OsCPK9          ---------DHPALVRLRAAYEDA--DAVHLVMELCDGGELFDRIVARG----RYTERAA
HvCPK29         ---------SHVNVVRLREAFEDD--DSVHLVMEVCEGGELFDRIVVRG----HYTERAA
OsCPK29         ---------AHANVVRLREAFEDA--DAVHLVMEVCEGGELFDRIVARG----HYTERAA
HvCPK21         --ARGG-----AAVVRLREAREDQ-DGSVHLVMELCEGGELFDRIVARG----HYSERAA
OsCPK21         SSSRGGGAASSAAVVRLREACEDAADGSVHLVMELCEGGELFDRIVARG----HYSERAA
HvCPK22         --ALEGA---EGTVVRLRGACEDA--EGVHLVMELCEGGELFDRIFARG----HYTERAA
OsCPK22         --ALGAG---ADSVVRLRDACEDS--DGVHLVMELCEGGELFDRIFARG----HYTERAA
AtCPK24         ---------KHPNIVSFKEAFEDK--DAVYLVMEICEGGELFDRIVSRG----HYTERAA
HvCPK5          ---------GHENIVHFDNAFEDD--SYVYIVMELCEGGELLDRILAKKN--SRYSEKDA
OsCPK4          ---------GHENIVHFYNAFEDD--SYVYIVMELCEGGELLDRILAKKN--SRYSEKDA
HvCPK11         ---------GHENVVHFYNAFEDD--NYVYIVMELCEGGELLDRILAKKD--SRYSEKDA
OsCPK18         ---------GHENVVHFYNAFEDD--NYVYIVMELCEGGELLDRILAKKD--SRYSEKDA
```

```
AtCPK16     ---------GHENVVRFYNAFEDK--NSVYIVMELCEGGELLDRILARKD--SRYSERDA
AtCPK18     ---------GHENVVGFHNAFEDK--TYIYIVMELCDGGELLDRILAKKD--SRYTEKDA
AtCPK28     ---------GHENVVQFHNAFEDD--DYVYIVMELCEGGELLDRILSKKG--NRYSEKDA
                          :  :     **      : ::** * *.** .:*          ::*: *
```

KINASE DOMAIN

ACTIVE SITE

```
HvCPK6a     AEIARVIVGVVEACHSLGVMHRDLKPENFLLKDREGNGDSSNGSAGNNEREPGEKINLKA
OsCPK6      AEITRVIVGVVEACHSLGVMHRDLKPENFLLKE------SSSSSS------------LKA
HvCPK4      AELTRIIVGVVEACHSLGVMHRDLKPENFLLAN------KDDDLS------------LKA
OsCPK13     AELTRIIVGVVEACHSLGVMHRDLKPENFLLAN------KDDDLS------------LKA
HvCPK5a     AELTRIVVGVVEACHSLGVIHRDLKPENFLLAN------KDDDMS------------LKA
OsCPK5      AELTRIIVGVIEACHSLGVIHRDLKPENFLLAN------KDDDLS------------LKA
AtCPK5      AELTKIIVGVVEACHSLGVMHRDLKPENFLLVN------KDDDFS------------LKA
AtCPK6      AELTKIIVGVVEACHSLGVMHRDLKPENFLLVN------KDDDFS------------LKA
AtCPK26     AELIKIIVGVVEACHSLGVMHRDLKPENFLLVN------KDDDFS------------LKA
OsCPK7      AELIRIIVSIVAMCHSLGVMHRDLKPENFLLLD------KDDDLS------------IKA
OsCPK23     AELIKIIVGIIETCHSHGVMHRDLKPENFLLLD------ADDEFS------------VKA
HvCPK2      AQLIRTIVGVVEACHSLGVMHRDLKPENFLFAS------TAEDAP------------LKT
HvCPK24     AQLIRTIVGVVEACHSLGVMHRDLKPENFLFAS------TAEDAP------------LKT
OsCPK24     AQLIRTIVAVVEGCHSLGVMHRDLKPENFLFAS------AAEDAP------------LKA
HvCPK28     AQLIRTIVGVVEGCHSLGVMHRDLKPENFLFAS------TAEDAP------------LKA
OsCPK28     ALLIRTIVGVVEGCHSLGVMHRDLKPENFLFAS------TAEDAP------------LKA
AtCPK4      AKLIKTILGVVEACHSLGVMHRDLKPENFLFDS------PSDDAK------------LKA
AtCPK11     VKLIKTILGVVEACHSLGVMHRDLKPENFLFDS------PKDDAK------------LKA
AtCPK12     AKLIKTIVGVVEACHSLGVVHRDLKPENFLFSS------SDEDAS------------LKS
HvCPK17     AQLARVIIGIVEACHSLGVMHRDLKPENFLFVN------QKEDSP------------LKT
OsCPK17     AQLARVIIGVVEACHSLGVMHRDLKPENFLFVN------HKEDSP------------LKT
HvCPK11a    AQLTRVIVGVIEACHSLGVMHRDLKPENFLFIN------NQEDSP------------LKA
OsCPK11     AQLARVIVGVIEACHSLGVMHRDLKPENFLFIH------QKEDSP------------LKA
HvCPK8      AELARVIVGVVEVCHSMGVMHRDLKPENFLFVD------QTEEAA------------LKT
OsCPK10     AELARVIVGVVEVCHSMGVMHRDLKPENFLFAD------QTEEAA------------LKT
OsCPK27     AGLARVIVAVVESCHSLGVMHRDLKPENFLFVG------NEEDAP------------LKT
AtCPK20     AELARIIVGVIEACHSLGVMHRDLKPENFLFVS------GDEEAA------------LKT
AtCPK1      AELTRTIVGVVEACHSLGVMHRDLKPENFLFVS------KHEDSL------------LKT
AtCPK2      AELARTIVGVLEACHSLGVMHRDLKPENFLFVS------REEDSL------------LKT
AtCPK25     AHLAKVILGVVQTCHSLGVMHRDLKPENFLFVN------DDEDSP------------LKA
HvCPK15     ATLCREVVSVVHSCHSMGVMHRDLKPENFLFLN------KRED------------SPLKA
OsCPK15     AALCREIVSVVHSCHSMGVMHRDLKPENFLFLN------KRED------------SPLKA
HvCPK1      ALLCREMVSVVHSCHSMGVFHRDLKPENFLFLN------NKED------------SPLKA
OsCPK1      AALCREIVAVVHSCHSMGVFHRDLKPENFLFLS------KSED------------SPLKA
AtCPK3      ADLCRQMVMVVHSCHSMGVMHRDLKPENFLFLS------KDEN------------SPLKA
HvCPK2a     ASLLRTIVEIIHTCHSLGVIHRDLKPENFLLLS------KEED------------APLKA
OsCPK2      ASLLRTIVEIIHTCHSLGVIHRDLKPENFLLLS------KDED------------APLKA
HvCPK14     ASLVRTIMGIIHTCHTMGVIHRDLKPENFLLLS------KDED------------APLKA
OsCPK14     SSLLRTIVEIIHTCHSMGVIHRDLKPENFLLLS------KDEH------------APLKA
AtCPK17     ASLLRTIVQIVHTCHSMGVIHRDLKPENFLLLN------KDEN------------SPLKA
AtCPK34     ASLLRTIVQIIHTCHSMGVIHRDLKPENFLLLS------KDEN------------SPLKA
OsCPK25     AALLRAIVGIVHTCHSMGVIHRDLKPENFLLLS------KGDD------------APLKA
OsCPK26     AALLRAIVGIVHTCHSMGVIHRDLKPENFLLLS------KGDD------------APLKA
HvCPK25     ASLLRSVVGTVHTFHSMGVMHRDLKPENFLMLN------RDES------------SPIKA
HvCPK12     AAVCRDILTVVHVCHFMGVLHRDLKPENFLLAS------PADE------------APLKA
OsCPK12     AAVCRDILTVVHVCHFMGVIHRDLKPENFLLAS------ADDD------------APLKA
AtCPK29     ANIFRQIVNVVHVCHFMGVVHRDLKPENFLLVS------NEED------------SPIKA
HvCPK3      ATICRGVVNVVNVCHFMGVMHRDLKPENFLLAT------KDEN------------AVLKA
OsCPK19     ATICRAVVNVVNICHFMGVMHRDLKPENFLLAT------KEEN------------AMLKA
AtCPK9      ASVCRQIVNVVKICHFMGVLHRDLKPENFLLSS------KDEK------------ALIKA
AtCPK33     ASVCRQIVNVVNICHFMGVMHRDLKPENFLLSS------KDEK------------ALIKA
AtCPK21     AGIIRSIVNVVQICHFMGVVHRDLKPENFLLSS------KEEN------------AMLKA
AtCPK23     AGTIKSIVDVVQICHLNGVIHRDLKPENFLFSS------KEEN------------AMLKV
AtCPK15     AGVIRSVLNVVQICHFMGVIHRDLKPENFLLAS------TDEN------------AMLKA
AtCPK19     AEIIRSVVKVVQICHFMGVIHRDLKPENFLLSS------KDEAS-----------SMLKA
AtCPK27     AGIIRSIVNVVKNCHYMGVMHRDLKPENFLLTS------NDDN------------ATVKV
AtCPK31     VEIIRPIVNVVKNCHYMGVMLRDLKPENFLLSS------TDKN------------ATVKA
AtCPK22     AGIFRSIVNAVKICHSLDVVHRDLKPENFLFSS------KDEN------------AMLKA
```

```
HvCPK6          AAVTRTIVEVVQMCHRNGVIHRDLKPENFLYAN------KKESSP------------LKA
OsCPK8          AAVTRTIVEVVQMCHRHGVMHRDLKPENFLYAN------KKDSSP------------LKA
HvCPK7          AVVTKTIVEVVQMCHKHGVMHRDLKPENFLFAN------KKETAA------------LKA
OsCPK20         ALVTRTIVEVVQMCHKHGVMHRDLKPENFLFAN------KKETAA------------LKA
AtCPK7          AAVMKTIVEVVQICHKQGVMHRDLKPENFLFAN------KKETSA------------LKA
AtCPK8          AAVMKTILEVVQICHKHGVMHRDLKPENFLFAN------KKETSA------------LKA
AtCPK14         ASVIKTIIEVVQMCHKHGVMHRDLKPENFLFAN------KKETAS------------LKA
AtCPK32         AAVTKTIMEVVQVCHKHGVMHRDLKPENFLFGN------KKETAP------------LKA
OsCPK3          ANVTRTIVEVVQLCHRHGVIHRDLKPENFLFAN------KKENSP------------LKA
OsCPK16         AAVTRTIVEVVQLCHRHGVIHRDLKPENFLFAN------KKENSP------------LKA
HvCPK10         ANVTRTIVEVVQLCHRHGVIHRDLKPENFLFAN------KKENSP------------LKA
AtCPK13         AGVTKTIVEVVQLCHKHGVIHRDLKPENFLFAN------KKENSP------------LKA
AtCPK10         AAVARTIAEVVMMCHSNGVMHRDLKPENFLFAN------KKENSP------------LKA
AtCPK30         ATVARTIAEVVRMCHVNGVMHRDLKPENFLFAN------KKENSA------------LKA
OsCPK9          AAAARTVAEVVRACHAHGVMHRDLKPENFLYAG------KAEDAQ------------LKA
HvCPK29         AAVMRTIMEVVQHCHQNGVMHRDLKPENFLYAN------ASESSL------------LKV
OsCPK29         AAVMRTIMDVVQHCHKNGVMHRDLKPENFLYAN------ASENSP------------LKV
HvCPK21         AKIFRTIVNVIQICHSNGVIHRDLKPENFLFAN------KSEDAA------------LKV
OsCPK21         ANIFRTIVDVVQLCHSNGVIHRDLKPENFLFAN------KSEDSP------------LKV
HvCPK22         AKIGRTIARVVQLCHDNGVMHRDLKPENFLFAG------KEEDSP------------LKA
OsCPK22         AKLARTIVGVVQLCHENGVMHRDLKPENFLFAN------KSEDSP------------LKA
AtCPK24         ASVAKTILEVVKVCHEHGVIHRDLKPENFLFSN------GTETAQ------------LKA
HvCPK5          AVVVRQMLKVAAECHLRGLVHRDMKPENFLFKS------TKEDSP------------LKA
OsCPK4          AVVVRQMLKVAAECHLHGLVHRDMKPENFLFKS------TKEDSP------------LKA
HvCPK11         AVVVRQMLKVAAECHLHGLVHRDMKPENFLFKS------SKEGSP------------LKA
OsCPK18         AVVVRQMLKVAAECHLHGLVHRDMKPENFLFKS------TKEDSS------------LKA
AtCPK16         AVVVRQMLKVAAECHLRGLVHRDMKPENFLFKS------TEEDSP------------LKA
AtCPK18         AVVVRQMLKVAAECHLRGLVHRDMKPENFLFKS------TEEGSS------------LKA
AtCPK28         AVVVRQMLKVAGECHLHGLVHRDMKPENFLFKS------AQLDSP------------LKA
                    : :       *  .:. **:******                           :*
```

KINASE DOMAIN

```
HvCPK6a         IDFGLSVFFKPGQIFTDVVGSPYYVAPEVLCKHYGPEADVWTAGVIVYILLSGVPPFWAE
OsCPK6          IDFGLSVFFKPGQVFSDVVGSPYYVAPEVLCKHYGPEADVWTAGVIVYILLSGVPPFWAE
HvCPK4          IDFGLSVFFKPGQIFTDVVGSPYYVAPEVLCKKYGPEADVWTAGVILYILLSGVPPFWAE
OsCPK13         IDFGLSVFFKPGQTFTDVVGSPYYVAPEVLLKHYGPEADVWTAGVILYILLSGVPPFWAE
HvCPK5a         IDFGLSVFFKPGQVFTDVVGSPYYVAPEVLRKRYGPEADVWTAGVILYILLSGVPPFWAE
OsCPK5          IDFGLSVFFKPGQVFTDVVGSPYYVAPEVLRKCYGPEADVWTAGVILYILLSGVPPFWAE
AtCPK5          IDFGLSVFFKPGQIFTDVVGSPYYVAPEVLLKRYGPEADVWTAGVILYILLSGVPPFWAE
AtCPK6          IDFGLSVFFKPGQIFKDVVGSPYYVAPEVLLKHYGPEADVWTAGVILYILLSGVPPFWAE
AtCPK26         IDFGLSVFFKPGQIFEDVVGSPYYVAPEVLLKHYGPEADVWTAGVILYILVSGVPPFWAE
OsCPK7          IDFGLSVFFKPGQVFTELVGSPYYVAPEVLHKRYGPESDVWSAGVILYVLLSGVPPFWAE
OsCPK23         IDFGLSVFFRPGQVFREVVGSPYYIAPEVLEKRYGPEADIWTAGVILYVLLTGVPPFWAD
HvCPK2          TDFGLSMFYKPGDKFSDVVGSPYYVAPEVLQKCYGPEADVWSAGVILYILLCGVPPFWAE
HvCPK24         TDFGLSMFYKPGDKFSDVVGSPYYVAPEVLQKCYGPEADVWSAGVILYILLCGVPPFWAE
OsCPK24         TDFGLSMFYKPGDKFSDVVGSPYYVAPEVLQKCYGPESDVWSAGVILYILLCGVPPFWAE
HvCPK28         TDFGLSVFYKPGDKFADVVGSPYYVAPEVLLKCYGPEADVWSAGVILYILLCGVPPFWAE
OsCPK28         TDFGLSVFYKPGDKFSDVVGSPYYVAPEVLQKIYGPEADVWSAGVILYILLCGVPPFWAE
AtCPK4          TDFGLSVFYKPGQYLYDVVGSPYYVAPEVLKKCYGPEIDVWSAGVILYILLSGVPPFWAE
AtCPK11         TDFGLSVFYKPGQYLYDVVGSPYYVAPEVLKKCYGPEIDVWSAGVILYILLSGVPPFWAE
AtCPK12         TDFGLSVFCTPGEAFSELVGSAYYVAPEVLHKHYGPECDVWSAGVILYILLCGFPPFWAE
HvCPK17         IDFGLSIFFKPGGIYSDVVGSPYYVAPEVLLKQYGCEVDVWSAGVIIYILLSGVPPFWDE
OsCPK17         IDFGLSIFFKPGENYSDVVGSPYYVAPEVLMKHYGREVDVWSAGVIIYILLSGVPPFWDE
HvCPK11a        IDFGLSIFFTPGQMFTDVVGSPYYVAPEVLLKNYGREVDVWSAGVIIYILLSGVPPFWDE
OsCPK11         IDFGLSIFFKPGETFTDVVGSPYYVAPEVLMKHYGREVDVWSAGVIIYILLSGVPPFWDE
HvCPK8          IDFGLSVFFRPGQIFTDVVGSPYYVAPEVLKKKYGPEADVWSAGVIIYILLCGVPPFWAE
OsCPK10         IDFGLSIFFRPGQVFTDVVGSPYYVAPEVLKKKYGQEADVWSAGVIIYILLCGVPPFWAE
OsCPK27         IDFGLSMFFRPGEVFTDVVGSPYYVAPEVLKKSYGQEADVWSAGVIIYILLCGVPPFWAE
AtCPK20         IDFGLSVFFKPGETFTDVVGSPYYVAPEVLRKHYSHECDVWSAGVIIYILLSGVPPFWDE
AtCPK1          IDFGLSMFFKPDDVFTDVVGSPYYVAPEVLRKRYGPEADVWSAGVIVYILLSGVPPFWAE
AtCPK2          IDFGLSMFFKPDEVFTDVVGSPYYVAPEVLRKRYGPESDVWSAGVIVYILLSGVPPFWAE
AtCPK25         IDFGLSMFLKPGENFTDVVGSPYYIAPEVLNKNYGPEADIWSAGVMIYVLLSGSAPFWGE
HvCPK15         TDFGLSVFFKPGEQFRDLVGSAYYVAPEVLKRRYGAEADIWSAGVILYILLSGVPPFWAE
OsCPK15         TDFGLSVFFKPGEQFRDLVGSAYYVAPEVLKRLYGAEADIWSAGVILYILLSGVPPFWAE
HvCPK1          TDFGLSVFFKHGEQFKDLVGSAYYVAPEVLKRHYGAEADIWSAGIILYILLSGVPPFWAD
OsCPK1          TDFGLSVFFKPGEHFKDLVGSAYYVAPEVLKRNYGAEADIWSAGVILYILLSGVPPFWAE
```

```
AtCPK3          TDFGLSVFFKPGDKFKDLVGSAYYVAPEVLKRNYGPEADIWSAGVILYILLSGVPPFWGE
HvCPK2a         TDFGLSVFFKQGEVFKDIVGSAYYIAPEVLKRNYGPEADIWSVGVILYILLCGVPPFWAE
OsCPK2          TDFGLSVFFKQGEVFKDIVGSAYYIAPEVLKRSYGPEADIWSVGVILYILLCGVPPFWAE
HvCPK14         TDFGLSVFFKEGEVFRDIVGSAYYIAPEVLKRNYGPQADIWSVGVMLYILLCGVPPFWAQ
OsCPK14         TDFGLSVFFKEGEVFRDIVGSAYYIAPEVLKRSYGPEADIWSIGVMLYILLCGVPPFWAE
AtCPK17         TDFGLSVFYKPGEVFKDIVGSAYYIAPEVLKRKYGPEADIWSIGVMLYILLCGVPPFWAE
AtCPK34         TDFGLSVFYKPGEVFKDIVGSAYYIAPEVLRRKYGPEADIWSIGVMLYILLCGVPPFWAE
OsCPK25         TDFGLSVFFKEGEVFRDIVGSAYYIAPEVLKRKYGPEADIWSIGVMLYIFLAGVPPFWAE
OsCPK26         TDFGLSVFFKEGEVFRDIVGSAYYIAPEVLKRKYGPEADIWSIGVMLYIFLAGVPPFWAE
HvCPK25         TDFGLSVFFKEGEVFKDIVGSAYYIAPEVLKRKYGHEADVWSIGVMLYIFLSGVPPFWAE
HvCPK12         IDFGLSVFIEEGKVYKDIVGSAYYVAPEVLQRNYGREIDVWSAGVILYILLCGSPPFWAE
OsCPK12         IDFGLSVFIEEGKVYKDIVGSAYYVAPEVLQRNYGKEADIWSAGVILYILLCGTPPFWAE
AtCPK29         TDFGLSVFIEEGKVYRDIVGSAYYVAPEVLHRNYGKEIDVWSAGVMLYILLSGVPPFWGE
HvCPK3          TDFGLSVFIEEGKMYRDIVGSAYYVAPEVLRRNYGKEIDVWSAGVILYILLSGVPPFWAE
OsCPK19         TDFGLSVFIEEGKMYRDIVGSAYYVAPEVLRRNYGKEIDVWSAGVILYILLSGVPPFWAE
AtCPK9          TDFGLSVFIEEGKVYRDIVGSAYYVAPEVLRRRYGKEVDIWSAGIILYILLSGVPPFWAE
AtCPK33         TDFGLSVFIEEGRVYKDIVGSAYYVAPEVLKRRYGKEIDIWSAGIILYILLSGVPPFWAE
AtCPK21         TDFGLSVFIEEGKVYRDIVGSAYYVAPEVLRRSYGKEIDIWSAGVILYILLSGVPPFWAE
AtCPK23         TDFGLSAFIEEGKIYKDVVGSPYYVAPEVLRQSYGKEIDIWSAGVILYILLCGVPPFWAD
AtCPK15         TDFGLSVFIEEGKVYRDIVGSAYYVAPEVLRRSYGKEIDIWSAGIILYILLCGVPPFWSE
AtCPK19         TDFGVSVFIEEGKVYEDIVGSAYYVAPEVLKRNYGKAIDIWSAGVILYILLCGNPPFWAE
AtCPK27         IDFGCSVFIEEGKVYQDLAGSDYYIAPEVLQGNYGKEADIWSAGIILYILLCGKSPFVKE
AtCPK31         IDFGCSVFIEEGEVHRKFAGSAYYIAPEVLQGKYGKEADIWSAGIILYILLCGKPPFVTE
AtCPK22         IDFGCSVYIKEGKTFERVVGSKYYIAPEVLEGSYGKEIDIWSAGVILYILLSGVPPFQTG
HvCPK6          IDFGLSVFFRPGERFSEIVGSPYYMAPEVLKRNYGPEIDVWSAGVILYILLCGVPPFWAE
OsCPK8          IDFGLSVFFRPGERFTEIVGSPYYMAPEVLKRHYGPEVDVWSAGVILYILLCGVPPFWAE
HvCPK7          IDFGLSVFFTPGERFTEIVGSPYYMAPEVLKRNYGQEVDVWSAGVILYILLCGVPPFWAE
OsCPK20         IDFGLSVFFTPGERFTEIVGSPYYMAPEVLKRNYGPEVDVWSAGVILYILLCGVPPFWAE
AtCPK7          IDFGLSVFFKPGEQFNEIVGSPYYMAPEVLRRNYGPEIDVWSAGVILYILLCGVPPFWAE
AtCPK8          IDFGLSVFFKPGEGFNEIVGSPYYMAPEVLRRNYGPEVDIWSAGVILYILLCGVPPFWAE
AtCPK14         IDFGLSVFFKPGERFNEIVGSPYYMAPEVLRRSYGQEIDIWSAGVILYILLCGVPPFWAE
AtCPK32         IDFGLSVFFKPGERFNEIVGSPYYMAPEVLKRNYGPEVDIWSAGVILYILLCGVPPFWAE
OsCPK3          IDFGLSIFFKPGEKFSEIVGSPYYMAPEVLKRNYGPEIDIWSAGVILYILLCGVPPFWAE
OsCPK16         IDFGLSIFFKPGEKFSEIVGSPYYMAPEVLKRNYGPEIDIWSAGVILYILLCGVPPFWAE
HvCPK10         IDFGLSIFFKPGEKFSEIVGSPYYMAPEVLKRNYGPEIDIWSAGVILYILLCGVPPFWAE
AtCPK13         IDFGLSIFFKPGEKFSEIVGSPYYMAPEVLKRNYGPEIDIWSAGVILYILLCGVPPFWAE
AtCPK10         IDFGLSVFFKPGDKFTEIVGSPYYMAPEVLKRDYGPGVDVWSAGVIIYILLCGVPPFWAE
AtCPK30         IDFGLSVLFKPGERFTEIVGSPYYMAPEVLKRNYGPEVDVWSAGVILYILLCGVPPFWAE
OsCPK9          IDFGLSVFFRPGERFREIVGSPYYMAPEVLRRDYGPEVDIWSAGVILYILLCGVPPFWAE
HvCPK29         VDFGLSVCFKPGERFNEIVGSPYYMAPEVLKRNYGQEIDIWSAGVILYILLCGVPPFWAE
OsCPK29         IDFGLSVCFKPGARFNEIVGSPYYMAPEVLKRNYGQEIDIWSAGVILYILLCGVPPFWAE
HvCPK21         IDFGLSVFFNPGDRFTEVVGSAYYMAPEVLKRNYGQEVDVWSAGVILYILLCGVPPFWGD
OsCPK21         IDFGLSVFFKPGDRFTEVVGSAYYMAPEVLRRSYGPEVDVWSAGVILYILLCGVPPFWGD
HvCPK22         IDFGLSVYFQPGERFTEVVGSGIYMAPEVLMRSYGPEADVWSAGVILYILLCGVPPFWGD
OsCPK22         IDFGLSVFFKPGERFTQVVGSTYYMAPEVLNRSYGPEADVWSAGVILYILLCGVPPFWGD
AtCPK24         IDFGLSIFFKPAQRFNEIVGSPYYMAPEVLRRNYGPEIDVWSAGVILYILLCGVPPFWAE
HvCPK5          TDFGLSDFIKPGKKFRDIVGSAYYVAPEVLKRRSGPESDVWSIGVITYILLCGRRPFWDK
OsCPK4          TDFGLSDFIKPGKKFHDIVGSAYYVAPEVLKRRSGPESDVWSIGVITYILLCGRRPFWNK
HvCPK11         TDFGLSDFITPGKQFHDIVGSAYYVAPEVLKRKSGPESDVWSIGVITYILLCGRRPFWDK
OsCPK18         TDFGLSDFIRPGKHFRDIVGSAYYVAPEVLKRKSGPESDVWSIGVITYILLCGRRPFWDK
AtCPK16         TDFGLSDFIKPGKKFHDIVGSAYYVAPEVLKRRSGPESDVWSIGVISYILLCGRRPFWDK
AtCPK18         TDFGLSDFIKPGVKFQDIVGSAYYVAPEVLKRRSGPESDVWSIGVITYILLCGRRPFWDK
AtCPK28         TDFGLSDFIKPGKRFHDIVGSAYYVAPEVLKRRSGPESDVWSIGVITYILLCGRRPFWDR
                 *** *           ..**  *:*****    .   *:*: *:: *::: *   **
```

KINASE DOMAIN

```
HvCPK6a         TQQGIFD-------AVLKGAIDFDSDPWPTISDGAKDLIRKMLKSPPAERFTAHQVLCHP
OsCPK6          TQQGIFD-------AVLRGSLDFDSDPWPTISDSAKDLIRRMLRSPPRERLTAHQVLCHP
HvCPK4          TQQGIFD-------AVLKGVIDFDSEPWPVISDSAKDLITRMLNPRPAERLTAHEVLCHP
OsCPK13         TQQGIFD-------AVLKGFIDFDSDPWPVISESAKDLITKMLNPRPKERLTAHEVLCHP
HvCPK5a         TQQGIFD-------AVLKGVVDFDSDPWPVISDSAKDLIMRMLNPRPAERLTAHQVLCHP
OsCPK5          TQQGIFD-------AVLKGVIDFDSDPWPVISDSAKDLIRRMLNPRPKERLTAHEVLCHP
AtCPK5          TQQGIFD-------AVLKGYIDFESDPWPVISDSAKDLIRRMLSSKPAERLTAHEVLRHP
AtCPK6          TQQGIFD-------AVLKGYIDFDTDPWPVISDSAKDLIRKMLCSSPSERLTAHEVLRHP
AtCPK26         TQQGIFD-------AVLKGHIDFDSDPWPLISDSAKNLIRGMLCSRPSERLTAHQVLRHP
OsCPK7          TQQGIFD-------AVLKGHIDFQSDPWPKISDSAKDLIRKMLSHCPSERLKAHEVLRHP
```

```
OsCPK23    TQSGIYE-------KVLDGRIDFKSNRWPRISDSAKDLIKKMLCPYPSERLKAHEVLKHP
HvCPK2     TEAGIFR-------QILRGKLDFESEPWPSISDSAKDLVRTMLCRDPTKRLSAHEVLCHP
HvCPK24    TEAGIFR-------QILRGKLDFESEPWPSISDSAKDLVRTMLCRDPTKRLSAHEVLCHP
OsCPK24    TEAGIFR-------QILRGKLDFESEPWPSISDSAKDLVRNMLCRDPTKRLTAHEVLCHP
HvCPK28    SETGIFK-------QILRGKLDLESEPWPSISDSAKDLVRKMLIRDPTKRQTAHEVLCHP
OsCPK28    TESGIFR-------QILRGKLDLESDPWPSISDSAKDLVRNMLIRDPTKRFTAHEVLCHP
AtCPK4     TESGIFR-------QILQGKIDFKSDPWPTISEGAKDLIYKMLDRSPKKRISAHEALCHP
AtCPK11    TESGIFR-------QILQGKLDFKSDPWPTISEAAKDLIYKMLERSPKKRISAHEALCHP
AtCPK12    SEIGIFR-------KILQGKLEFEINPWPSISESAKDLIKKMLESNPKKRLTAHQVLCHP
HvCPK17    SEQGIFE-------QVLKGDLDFSSEPWPSISKSAKDLVRKMLNRDPGKRLTAHEALCHP
OsCPK17    SEQGIFE-------KVLKGDLDFSSDPWPAISDSAKDLVRKMLNRDPRKRLTAHEALCHP
HvCPK11a   SEQGIFE-------QVLKGELDFTTEPWPSISDSAKDLVRKMLIRDPKKRLTAHEALCHP
OsCPK11    SEQGIFE-------QVLKGDLDFSSEPWPNISESAKDLVRKMLIRDPKKRLTAHEALCHP
HvCPK8     NEQGIFE-------EVLHGKLDFESDPWPSISEGAKDLVRRMLLRDPKKRLTAHEVLRHP
OsCPK10    NEQGIFE-------EVLHGRLDFQSEPWPSISEGAKDLVRRMLVRDPKKRLTAHEVLRHP
OsCPK27    TEQGIFE-------QVLHGTLDFESDPWPNVSDGAKDLLRKVLVRDPKKRLTAHEVLCHP
AtCPK20    TEQGIFE-------QVLKGDLDFISEPWPSVSESAKDLVRRMLIRDPKKRMTTHEVLCHP
AtCPK1     TEQGIFE-------QVLHGDLDFSSDPWPSISESAKDLVRKMLVRDPKKRLTAHQVLCHP
AtCPK2     TEQGIFE-------QVLHGDLDFSSDPWPSISESAKDLVRKMLVRDPKRRLTAHQVLCHP
AtCPK25    TEEEIFN-------EVLEGELDLTSDPWPQVSESAKDLIRKMLERNPIQRLTAQQVLCHP
HvCPK15    NEEGIFD-------AVLKGHIDFSSDPWPSISNGAKDLVKRMLRQDPKERLTAAEILNHP
OsCPK15    NEDGIFD-------AVLQGHIDFSSEPWPSISSGAKDLVKRMLRQDPKERLTAAEILNHP
HvCPK1     NEDGIFE-------AVLLGHIDFSSDPWPSISNGAKDLVKKMLRQDPKERLTAAEILNHP
OsCPK1     SEDGIFD-------AVLRGHIDFSSEPWPSISNGAKDLVKKMLRQDPKERLTSAEILNHP
AtCPK3     NETGIFD-------AILQGQLDFSADPWPALSDGAKDLVRKMLKYDPKDRLTAAEVLNHP
HvCPK2a    SEHGIFN-------SILRGQVDFTSDPWPRISPAAKDLVRKMLNSDPKKRISAYDVLNHA
OsCPK2     SEHGIFN-------SILRGQVDFTSDPWPRISASAKDLVRKMLNSDPKKRISAYEVLNHP
HvCPK14    SEHGIFN-------SILRGQVDFNSDPWPRISGGAKDLVRKMLTSDPKKRISAHDVLNHP
OsCPK14    SEHGIFN-------SILRGHVDFSSEPWSRISHGAKDLVRRMLHSDPKQRISAYDVLNHP
AtCPK17    SENGIFN-------AILRGHVDFSSDPWPSISPQAKDLVKKMLNSDPKQRLTAAQVLNHP
AtCPK34    SENGIFN-------AILSGQVDFSSDPWPVISPQAKDLVRKMLNSDPKQRLTAAQVLNHP
OsCPK25    SENAIFT-------AILRGQIDLASEPWPKISSGAKDLVRKMLNINPKERLTAFQVLNHP
OsCPK26    SENAIFA-------AILRGQIDLASEPWPKISSGAKDLVRKMLNINPKERLTAFQVLNHP
HvCPK25    NENAIFT-------AILRGEVDFASDPWPNISAGAKDLVRKMLHINPKERLTAIQVLNHP
HvCPK12    TEKGIFD-------AILVGQLDFSSSPWPTISESAKDLIRQMLSRDPKRRITAVQALEHP
OsCPK12    TEKGIFD-------AILVNQVDFSTSPWPSISESAKDLIRQMLHRDPQKRITASQALEHR
AtCPK29    TEKTIFE-------AILEGKLDLETSPWPTISESAKDLIRKMLIRDPKKRITAAEALEHP
HvCPK3     TEKGIFD-------AILQGEIDFESQPWPSISESAKDLVRKMLAQDPKKRISSAQVLQHP
OsCPK19    TEKGIFD-------AILQGEIDFESQPWPSISESAKDLVRKMLTQDPKKRITSAQVLQHP
AtCPK9     TEKGIFD-------AILEGHIDFESQPWPSISSSAKDLVRRMLTADPKRRISAADVLQHP
AtCPK33    TEKGIFD-------AILEGEIDFESQPWPSISNSAKDLVRRMLTQDPKRRISAAEVLKHP
AtCPK21    NEKGIFD-------EVIKGEIDFVSEPWPSISESAKDLVRKMLTKDPKRRITAAQVLEHP
AtCPK23    NEEGVFV-------EILKCKIDFVREPWPSISDSAKDLVEKMLTEDPKRRITAAQVLEHP
AtCPK15    TEKGIFN-------EIIKGEIDFDSQPWPSISESAKDLVRKLLTKDPKQRISAAQALEHP
AtCPK19    TDKGIFE-------EILRGEIDFESEPWPSISESAKDLVRNMLKYDPKKRFTAAQVLEHP
AtCPK27    PEGQMFN-------EIKSLEIDYSEEPWPLRDSRAIHLVKRMLDRNPKERISAAEVLGHP
AtCPK31    PEAQMFS-------EIKSAKIDVDSESWKFIDVKAKHLVNRMLNRNPKERISAAEVLGHP
AtCPK22    IESIIVSTLCIVDAEIKECRLDFESQPWPLISFKAKHLIGKMLTKKPKERISAADVLEHP
HvCPK6     TEQGVAQ-------AIIRSVVDFKRDPWPRVSEPAKDLVRRMLDPNPITRLTAAQVLEHP
OsCPK8     TEQGVAQ-------AIIRSVVDFKREPWPRVSEPAKDLVKRMLDPNPMTRLTAEQVLEHP
HvCPK7     TEQGVAQ-------AIIRSVIDFKRDPWPRVSDNAKDLVRGMLNPDPRRRLTAQQVLDHP
OsCPK20    TEQGVAQ-------AIIRSVIDFKRDPWPRVSDNAKDLVKGMLNPDPRRRLNAQQVLDHP
AtCPK7     TEQGVAQ-------AIIRSVIDFKRDPWPRVSDSAKDLVRKMLEPDPKKRLTAAQVLEHT
AtCPK8     TEQGVAQ-------AIIRSVIDFKRDPWPRVSETAKDLVRKMLEPDPKKRLSAAQVLEHS
AtCPK14    TEHGVAK-------AILKSVIDFKRDPWPKVSDNAKDLIKKMLHPDPRRRLTAQQVLDHP
AtCPK32    TEQGVAQ-------AIIRSVLDFRRDPWPKVSENAKDLIRKMLDPDQKRRLTAQQVLDHP
OsCPK3     TEQGVAQ-------AILRGNIDFKREPWPNVSENAKDLVRRMLEPDPKLRLTAKQVLEHP
OsCPK16    TEQGVAQ-------AILRGNIDFKREPWPNVSDNAKDLVRQMLQPDPKLRLTAKQVLEHT
HvCPK10    TEQGVAQ-------AILRGNIDFKREPWPNVSENAKDLVRRMLEPDPKLRLTAKQVLEHH
AtCPK13    SEQGVAQ-------AILRGVIDFKREPWPNISETAKNLVRQMLEPDPKRRLTAKQVLEHP
AtCPK10    TEQGVAL-------AILRGVLDFKRDPWPQISESAKSLVKQMLDPDPTKRLTAQQVLAHP
AtCPK30    TEQGVAL-------AILRGVLDFKRDPWSQISESAKSLVKQMLEPDSTKRLTAQQVLDHP
OsCPK9     TEQGVAR-------AILRGAADFDREPWPRISRAAKSLVRQMLDVDPRRRPTAQQVLDHP
HvCPK29    TDEGIAQ-------AIIRSRIDFEREPWPKVSENAKDLVSMMLENNPYTRLTAQQVLEHP
OsCPK29    TDEGIAQ-------AIIRSHIDFQREPWPKVSDNAKDLVRRMLDPNPYTRLTAQQVLEHP
HvCPK21    NDEKIAQ-------AILRGGLDFNREPWPRVSGNAKDLIRRMLDPDPATRLTAHQVLEHP
OsCPK21    NDEKIAQ-------AILRGAIDFNREPLPRVSANAKDLVRRMLDPNPSTRLTAKQVLEHP
HvCPK22    TDERIAE-------SIIRGEINFEREPWPKVSHTAKDLVKKMLDPDPATRLTANQVSEHP
```

```
OsCPK22           NDEKTVT-------AILQGGINFQREPWPKVSPHAKDLVSKMLDPDPSTRLTAKEVLEHP
AtCPK24           TEEGIAH-------AIVRGNIDFERDPWPKVSHEAKELVKNMLDANPYSRLTVQEVLEHP
HvCPK5            TEDGIFK-------EVLRNKPDFRKRPWPSISSSAKDFVKKLLVKNPRARLTAAQALSHP
OsCPK4            TEDGIFR-------EVLRNKPDFRKKPWPGISSGAKDFVKKLLVKNPRARLTAAQALSHP
HvCPK11           TEDGIFK-------EVLKNKPDFRRKPWANITPSAKDFVQKLLVKDPHARLTAAQALSHE
OsCPK18           TEDGIFK-------EVLKNKPDFRRKPWPNITPCAKDFVQKLLVKDPRARLTAAQALSHE
AtCPK16           TEDGIFK-------EVLKNKPDFRRKPWPTISNSAKDFVKKLLVKDPRARLTAAQALSHP
AtCPK18           TQDGIFN-------EVMRKKPDFREVPWPTISNGAKDFVKKLLVKEPRARLTAAQALSHS
AtCPK28           TEDGIFK-------EVLRNKPDFSRKPWATISDSAKDFVKKLLVKDPRARLTAAQALSHA
                   :             :     :            *  ::  :*      * .  :   *

                                                                      CLD
HvCPK6a           WIIENGEAPDAPLDPAVLSRLKQFSAMNRLKKMALRVIARGLSEEELAGLKEMFKTMDAD
OsCPK6            WVCDDGVAPDRPLAPAVLSRLKQFSAMNRLKKMALRVIARNLSEEELAGLKEMFKAMDTD
HvCPK4            WIRDHGVAPDRPLDTAVLSRIKQFSAMNKLKKMALRVIAESLSEEEIAGLKEMFQTMDAD
OsCPK13           WIRDHGVAPDRPLDPAVLSRIKQFSAMNKLKKMALRVIAESLSEEEIAGLKEMFQTMDAD
HvCPK5a           WISDHGVAPDRPLDPAVLSRIKQFSAMNKLKKMALRVIAESLSEEEIAGLKEMFEAMDTD
OsCPK5            WICDHGVAPDRPLDPAVLSRIKQFSAMNKLKKMALRVIAESLSEEEIAGLKEMFKAMDTD
AtCPK5            WICENGVAPDRALDPAVLSRLKQFSAMNKLKKMALKVIAESLSEEEIAGLREMFQAMDTD
AtCPK6            WICENGVAPDRALDPAVLSRLKQFSAMNKLKKMALKVIAESLSEEEIAGLRAMFEAMDTD
AtCPK26           WICENGVAPDRALDPAVLSRLKQFSAMNKLKQMALRVIAESLSEEEIAGLKEMFKAMDTD
OsCPK7            WICENGVATDQALDPSVISRLKQFSAMNKLKKLALRVIAERLSEEEIAGLREMFKAVDTK
OsCPK23           WICDNGVATNRALDPSVLPRLKQFSAMNRLKKLSLQIIAERLSEEEIVGLREMFKAMDTK
HvCPK2            WIVDDAVAPDKPIDSAVLSRLKHFSAMNKLKKMALRVIAESLSEEEIGGLRELFKMIDAD
HvCPK24           WIVDDAVAPDKPIDSAVLSRLKHFSAMNKLKKMALRVIAESLSEEEIGGLRELFKMIDAD
OsCPK24           WIVDDAVAPDKPIDSAVLSRLKHFSAMNKLKKMALRVIAESLSEEEIGGLKELFKMIDTD
HvCPK28           WIVDDSVAPDKPIDSAVLSRLKHFSAMNKLKKMALRVIAESLSEEEIGGLKELFKMIDTD
OsCPK28           WIVDDAVAPDKPIDSAVLSRLKHFSAMNKLKKMALRVIAESLSEEEIGGLKELFKMIDTD
AtCPK4            WIVDEHAAPDKPLDPAVLSRLKQFSQMNKIKKMALRVIAERLSEEEIGGLKELFKMIDTD
AtCPK11           WIVDEQAAPDKPLDPAVLSRLKQFSQMNKIKKMALRVIAERLSEEEIGGLKELFKMIDTD
AtCPK12           WIVDDKVAPDKPLDCAVVSRLKKFSAMNKLKKMALRVIAERLSEEEIGGLKELFKMIDTD
HvCPK17           WVCVDGVAPDKPLDSAVLTRLKQFSAMNKLKKMALRVIAENLSEDEIAGLREMFKMLDTD
OsCPK17           WVCVDGVAPDKPLDSAVLTRLKQFSAMNKLKKMALRVIAENLSEDEIAGLREMFKMLDTD
HvCPK11a          WVCVDGVAPDKPLDSAVLSRLKQFSAMNKLKKMALRVIAESLSEEEIAGLKEMFKMLDTD
OsCPK11           WVCVDGVAPDKPLDSAVLSRLKQFSAMNKLKKMALRVIAESLSEEEIAGLKEMFKMLDTD
HvCPK8            WVQVGGLAPDKPLDSAVLSRMKQFSAMNKLKKMALRVIAENLSEDEIAGLKEMFKMIDSD
OsCPK10           WVQVGGLAPDKPLDSAVLSRMKQFSAMNKLKKMALRVIAENLSEDEIAGLKEMFKMIDTD
OsCPK27           WLQMSGSAPDKPLDSAVLSRLRQFSAMNKLKKMALRVIAENLSEEEIAGLKEMFKMMDTD
AtCPK20           WARVDGVALDKPLDSAVLSRLQQFSAMNKLKKIAIKVIAESLSEEEIAGLKEMFKMIDTD
AtCPK1            WVQVDGVAPDKPLDSAVLSRMKQFSAMNKFKKMALRVIAESLSEEEIAGLKEMFNMIDAD
AtCPK2            WVQIDGVAPDKPLDSAVLSRMKQFSAMNKFKKMALRVIAESLSEEEIAGLKQMFKMIDAD
AtCPK25           WIRDEGNAPDTPLDTTVLSRLKKFSATDKLKKMALRVIAERLSEEEIHELRETFKTIDSG
HvCPK15           WIREDGEAPDKPLDITVISRMKQFRAMNKLKKVALKIVAESLSEEEIVGLREMFKSLDTD
OsCPK15           WIREDGEAPDKPLDITVISRMKQFRAMNKLKKVALKVVAENLSEEEIVGLKEMFKSLDTD
HvCPK1            WIREDGEAPDKPLDITVISRMKQFRAMNKLKKVALKIVAENLSEEEITGLKEMFRSLDTD
OsCPK1            WIREDGEAPDKPLDITVISRMKQFRAMNKLKKVALKVVAENLSDEEITGLKEMFRSLDTD
AtCPK3            WIREDGEASDKPLDNAVLSRMKQFRAMNKLKKMALKVIAENLSEEEIIGLKEMFKSLDTD
HvCPK2a           WIKEDGEAPDTPLDNAVMNRLKQFKAMNQFKKAALRVIAGCLSEEEIRGLKEMFKSMDSD
OsCPK2            WIKEDGEAPDTPLDNAVMNRLKQFRAMNQFKKAALRVIAGCLSEEEIRGLKEMFKSMDSD
HvCPK14           WIKEDGEAPDTLIDNAVLGRLKQFRAMNQFKKAALRVIAGCLSEEEIRGLKEMFQSMDSD
OsCPK14           WIKEDGEAPDTPLDNAVLGRLKQFRAMNQFKKAALRVIAGCLSEEEIRGLKEMFKSMDSD
AtCPK17           WIKEDGEAPDVPLDNAVMSRLKQFKAMNNFKKVALRVIAGCLSEEEIMGLKEMFKGMDTD
AtCPK34           WIKEDGEAPDVPLDNAVMSRLKQFKAMNNFKKVALRVIAGCLSEEEIMGLKEMFKGMDTD
OsCPK25           WIKEDGDAPDVPLDNVVLNRLKQFRAMNQFKKAALRIIAGCLSEEEIKGLKEMFKNIDKD
OsCPK26           WIKEDGDAPDVPLDNVVLNRLKQFRAMNQFKKAALRIIAGCLSEEEIKGLKEMFKNIDKD
HvCPK25           WIKEDGEAPDTPLDNVVLDRMKQFVAMNQFKKAALRVIAGCLSEEEIKGLKEMFKSIDKD
HvCPK12           WLKEG-GASDRPIDSAVLLRMKQFKAMNKLKQLALKVIAENLSPEEIKGLKQMFNNMDTD
OsCPK12           WLKEG-GASDRPIDSAVLSRMKQFKAMNKLKQLALKVIAENLSPEEIKGLKQMFNNMDTD
AtCPK29           WMTDT-KISDKPINSAVLVRMKQFRAMNKLKKLALKVIAENLSEEEIKGLKQTFKNMDTD
HvCPK3            WLREG-EASDKPIDSAVLSRMKQFRAMNKLKKMALKVIASNLNEEEIKGLKQMFMNMDTD
OsCPK19           WLRDG-EASDKPIDSAVLSRMKQFRAMNKLKKMALKVIASNLNEEEIKGLKQMFTNMDTD
AtCPK9            WLREGGEASDKPIDSAVLSRMKQFRAMNKLKKLALKVIAENIDTEEIQGLKAMFANIDTD
AtCPK33           WLREGGEASDKPIDSAVLSRMKQFRAMNKLKKLALKVIAENIDTEEIQGLKAMFANIDTD
AtCPK21           WIK-GGEAPDKPIDSAVLSRMKQFRAMNKLKKLALKVIAESLSEEEIKGLKTMFANIDTD
AtCPK23           WIK-GGEAPEKPIDSTVLSRMKQFRAMNKLKKLALKVSAVSLSEEEIKGLKTLFANMDTN
AtCPK15           WIR-GGEAPDKPIDSAVLSRMKQFRAMNKLKKLALKVIAESLSEEEIKGLKTMFANMDTD
AtCPK19           WIREGGEASDKPIDSAVLSRMKQLRAMNKLKKLAFKFIAQNLKEEELKGLKTMFANMDTD
```

```
AtCPK27          WMKEG-EASDKPIDGVVLSRLKRFRDANKFKKVVLKFIAANLSEEEIKGLKTLFTNIDTD
AtCPK31          WMKDG-EASDKPIDGVVLSRLKQFRDMNKLKKVALKVIAANLSEEEIKGLKTLFTNIDTD
AtCPK22          WMKS--EAPDKPIDNVVLSRMKQFRAMNKLKKLALKVIAEGLSEEEIKGLKTMFENMDMD
HvCPK6           WLHDSKKNPDIQLGDTVRARLQQFSAMNKLKKKALRVIAEHLSLEEVADIKKMFDNMDIN
OsCPK8           WLHDSKKMPDIPLGDAVRARLQQFAAMNKLKKKALKVIAEHLSAEEAADIKDMFDKMDVS
HvCPK7           WLQNIKKAPNVNLGETVKARLQQFSVMNKFKKHALRVIAEHLSVEEVAGIKDMFEKMDLN
OsCPK20          WLQNIKKAPNVNLGETVKARLQQFSVMNKFKKHALRVIAEHLSVEEVAGIKDMFEKMDLN
AtCPK7           WILNAKKAPNVSLGETVKARLKQFSVMNKLKKRALRVIAEHLSVEEAAGIKEAFEMMDVN
AtCPK8           WIQNAKKAPNVSLGETVKARLKQFSVMNKLKKRALRVIAEHLSVEEVAGIKEAFEMMDSK
AtCPK14          WIQNGKNASNVSLGETVRARLKQFSVMNKLKKRALRVIAEHLSVEETSCIKERFQVMDTS
AtCPK32          WLQNAKTAPNVSLGETVRARLKQFTVMNKLKKRALRVIAEHLSDEEASGIREGFQIMDTS
OsCPK3           WLQNAKKAPNVPLGDIVKSRLKQFSRMNRFKRRALRVIADHLSAEEVEDIKEMFKAMDTD
OsCPK16          WLQNAKKAPNVPLGDIVKSRLKQFSRMNRFKRRALRVIADHLSAEEVEDIKDMFKVMDTD
HvCPK10          WLQNAKKAPNVPLGDIVKSRLKQFSRMNRFKRRALRVIADHLSAEEVEDIKEMFKAMDTD
AtCPK13          WIQNAKKAPNVPLGDVVKSRLKQFSVMNRFKRKALRVIAEFLSTEEVEDIKVMFNKMDTD
AtCPK10          WIQNAKKAPNVPLGDIVRSRLKQFSMMNRFKKKVLRVIAEHLSIQEVEVIKNMFSLMDDD
AtCPK30          WIQNAKKAPNVPLGDIVRSRLKQFSMMNRLKKKALRVIAEHLSIQEVEVIRNMFTLMDDD
OsCPK9           WLHHAARAPNVPLGDVVRARLKQFSLMNRLKKKAMRVIAEHLSVEEVEVIKDMFALMDTD
HvCPK29          WIQNATAAPNIPLGEAVRSRLKQFTVMNKFKKKALLVVAEYLPAEELEAITELFHMLDTN
OsCPK29          WIQNASAAPNIPLGEAVRSRLKQFTVMNKFKKKALLVVAEYLPTEELDAIRELFNMLDTK
HvCPK21          WLKNADTTPNVSLGEAVRSRLQQFSAMNKLKKKALGVVARNMPVEELDKYVQMFHLMDKD
OsCPK21          WLKNADTAPNVSLGDAVRARLQQFSAMNKFKKKALGVVARNLPGEEVDKYVQMFHHMDKD
HvCPK22          WLKNADKAPNVSLGELVRSRLKQFSSMNKFKKKALGVVAKSLPAEEIENYTQMFQTMDKD
OsCPK22          WLKNADRAPNVSLGEIVRSRLMQFSAMNKFKKKALGVVAKNLPVEEMDKYTQMFHKMDKD
AtCPK24          WIRNAERAPNVNLGDNVRTKIQQFLLMNRFKKKVLRIVADNLPNEEIAAIVQMFQTMDTD
HvCPK5           WVREGGDASEIPVDISVLYNMRQFVKYSRFKQFALRALASTVNEEELADLKDQFDAIDVD
OsCPK4           WVREGGEASEIPVDISVLSNMRQFVKYSRFKQFALRALASTLKEEELADLKDQFDAIDVD
HvCPK11          WVREGGQASEIPLDISVLHNMRQFVKYSRFKQFALRALASTLNPEELSDLRDQFNAIDID
OsCPK18          WVREGGQASDIPLDISVLHNMRQFVKYSRFKQFALRALASTLNAEELSDLRDQFNAIDVD
AtCPK16          WVREGGDASEIPIDISVLNNMRQFVKFSRLKQFALRALATTLDEEELADLRDQFDAIDVD
AtCPK18          WVKEGGEASEVPIDISVLNNMRQFVKFSRLKQIALRALAKTINEDELDDLRDQFDAIDID
AtCPK28          WVREGGNATDIPVDISVLNNLRQFVRYSRLKQFALRALASTLDEAEISDLRDQFDAIDVD
                 *        :  :   *  .: ::   ..:*:  :   *  :   *       *  :*
```

Calmodulin -like domain

```
HvCPK6a          GSGAITFEELREGLRRYGSTELGDSEVRDLMEA--ADVDRSGTIDYDEFIAATVHMNKLD
OsCPK6           ASGAITFDELKEGLRRYGS-NLREAEIRDLMDA--ADVDKSGTIDYDEFIAATVHLNKLE
HvCPK4           NSGAITYDELKEGLRKYGS-TLKDTEIRDLMDA--ADVDNSGTIDYIEFIAATLHLNKLE
OsCPK13          NSGAITYDELKEGLRKYGS-TLKDTEIRDLMDA--ADIDNSGTIDYIEFIAATLHLNKLE
HvCPK5a          NSGAITYDELKEGMRKYGS-TLKDTEIRDLMEA--ADVDNSGTIDYIEFIAATLHLNKLE
OsCPK5           NSGAITYDELKEGMRKYGS-TLKDTEIRDLMEA--ADVDNSGTIDYIEFIAATLHLNKLE
AtCPK5           NSGAITFDELKAGLRKYGS-TLKDTEIHDLMDA--ADVDNSGTIDYSEFIAATIHLNKLE
AtCPK6           NSGAITFDELKAGLRRYGS-TLKDTEIRDLMEA--ADVDNSGTIDYSEFIAATIHLNKLE
AtCPK26          NSGAITFDELKAGLRRYGS-TLKDTEIRDLMEA--ADIDKSGTIDYGEFIAATIHLNKLE
OsCPK7           NRGVITFGELREGLRRFGA-EFKDTEIGDIMEA--AHNDNNVTIHYEEFIAATLPLNKIE
OsCPK23          NRSVVTFGELKG-LKRYSS-VFKDTEINDLMEA--AD-DTTSTINWEEFIAAAVSLNKIE
HvCPK2           NSGTITFDELKDGLKRVGS-ELTEHEIQALMDA--ADIDNSGTIDYGEFIAATLHMNKLE
HvCPK24          NSGTITFDELKDGLKRVGS-ELTEHEIQALMDA--ADIDNSGTIDYGEFIAATLHMNKLE
OsCPK24          DSGTITFDELKEGLKRVGS-ELTEHEIQALMEA--ADIDNSGTIDYGEFIAATLHMNKLE
HvCPK28          NSGTITYEELRDGLKRVGS-DLMEPEIQSLMDA--ADIDNSGSIDYGEFLAATLHVNKLE
OsCPK28          NSGTITYDELKNGLKRVGS-DLMEPEIQALMDA--ADIDNSGTIDYGEFLAATLHMNKLE
AtCPK4           NSGTITFEELKAGLKRVGS-ELMESEIKSLMDA--ADIDNSGTIDYGEFLAATLHINKME
AtCPK11          NSGTITFEELKAGLKRVGS-ELMESEIKSLMDA--ADIDNSGTIDYGEFLAATLHMNKME
AtCPK12          KSGTITFEELKDSMRRVGS-ELMESEIQELLRA--ADVDESGTIDYGEFLAATIHLNKLE
HvCPK17          NSGQITLEELKTGLQRVGA-NLKESEIATLMEA--ADIDNSGSIDYGEFLAATLHLNKVE
OsCPK17          NSGQITLEELKTGLRRVGA-NLKDSEITTLMEA--ADIDNSGSIDYGEFIAATMHLNKVE
HvCPK11a         NSGHITLEELKSGLRRVGA-TLMDSEIDALMEA--ADIDNSGTIDYGEFIAATMHMNKVD
OsCPK11          NSGHITLEELKTGLQRVGA-NLMDSEIDALMEA--ADIDNSGTIDYGEFIAATLHINKVE
HvCPK8           NSGQITYEELKVGLKKVGA-NLQESEIYALMQA--ADVDNSGTIDYGEFIAATLHLNKVE
OsCPK10          NSGQITFEELKVGLKKVGA-NLQESEIYALMQA--ADVDNSGTIDYGEFIAATLHMNKIE
OsCPK27          NSGQINYEELKAGLERVGA-NMKESEIYQLMQA--ADIDNSGTIDYGEFIAATLHLNKVE
AtCPK20          NSGHITLEELKKGLDRVGA-DLKDSEILGLMQA--ADIDNSGTIDYGEFIAAMVHLNKIE
AtCPK1           KSGQITFEELKAGLKRVGA-NLKESEILDLMQA--ADVDNSGTIDYKEFIAATLHLNKIE
AtCPK2           NSGQITFEELKAGLKRVGA-NLKESEILDLMQA--ADVDNSGTIDYKEFIAATLHLNKIE
AtCPK25          KSGRVTYKELKNGLERFNT-NLDNSDINSLMQIP-TDVHLEDTVDYNEFIEAIVRLRQIQ
HvCPK15          NSGTITLDELRAGLPKLGT-KITESEIRQLMEAA--DVDGNGTIDYVEFISATMHMNRLE
OsCPK15          NSGTITLEELRAGLPKLGT-KISESELRQLMEAA--DVDGNGSIDYVEFISATMHMNRLE
```

```
HvCPK1            NSGTITLEELRSGLPKLGT-KISESEITQLMEAA--DVDGNGTIDYSEFVSATMHMNRLE
OsCPK1            NSGTITLEELRSGLPKLGT-KISESEIRQLMEAA--DVDGNGTIDYAEFISATMHMNRLE
AtCPK3            NNGIVTLEELRTGLPKLGS-KISEAEIRQLMEAA--DMDGDGSIDYLEFISATMHMNRIE
HvCPK2a           NSGTITVDELRKGLGKQGT-KLTEAEVEQLMEAA--DADGSGTIDYEEFITAAMHMNRMD
OsCPK2            NSGTITVDELRKGLSKQGT-KLTEAEVQQLMEAA--DADGNGTIDYDEFITATMHMNRMD
HvCPK14           NSGTITVDELRKGLAKKGT-KLTEAEVQQLMEAA--DADGNGTIDYDEFITATMHMNRMD
OsCPK14           NSGTITVDELRKGLAKKGT-KLTEAEVQQLMEAA--DADGNGTIDYEEFITATMHMNRMD
AtCPK17           SSGTITLEELRQGLAKQGT-RLSEYEVQQLMEAA--DADGNGTIDYGEFIAATMHINRLD
AtCPK34           NSGTITLEELRQGLAKQGT-RLSEYEVQQLMEAA--DADGNGTIDYGEFIAATMHINRLD
OsCPK25           NSGTITLEELKNGLAKQGT-KFSDNEIEQLMEAA--DADGNGIIDYEEFVTATVHMNKMD
OsCPK26           NSGTITLEELKNGLAKQGT-KFSDNEIEQLMEAA--DADGNGIIDYEEFVTATVHMNKMD
HvCPK25           NSGTITLEELKTGLAKQGT-KLSDHEIQQLMEAA--DADGNGLIDYEEFVTATMHMNRMD
HvCPK12           KSGTITVEELKIGLTKLGS-KISEAEVQKLMEAV--DVDKSGSIDYTEFLTAMMNKHKLE
OsCPK12           RSGTITVEELKVGLTKLGS-RISEAEVQKLMEAV--DVDKSGSIDYSEFLTAMINKHKLE
AtCPK29           ESGTITFDELRNGLHRLGS-KLTESEIKQLMEAA--DVDKSGTIDYIEFVTATMHRHRLE
HvCPK3            NSGTITYEELKAGLAKLGS-KLSEAEVKQLMDAA--DVDGNGSIDYVEFITATMHRHKLE
OsCPK19           NSGTITYEELKAGLAKLGS-KLSEAEVKQLMEAA--DVDGNGSIDYVEFITATMHRHKLE
AtCPK9            NSGTITYEELKEGLAKLGS-KLTEAEVKQLMDAA--DVDGNGSIDYIEFITATMHRHRLE
AtCPK33           NSGTITYEELKEGLAKLGS-RLTEAEVKQLMDAA--DVDGNGSIDYIEFITATMHRHRLE
AtCPK21           KSGTITYEELKTGLTRLGS-RLSETEVKQLMEAA--DVDGNGTIDYYEFISATMHRYKLD
AtCPK23           RSGTITYEQLQTGLSRLRS-RLSETEVQQLVEAS--DVDGNGTIDYYEFISATMHRYKLH
AtCPK15           KSGTITYEELKNGLAKLGS-KLTEAEVKQLMEAA--DVDGNGTIDYIEFISATMHRYRFD
AtCPK19           KSGTITYDELKSGLEKLGS-RLTETEVKQLLEDA--DVDGNGTIDYIEFISATMNRFRVE
AtCPK27           KSGNITLEELKTGLTRLGS-NLSKTEVEQLMEAA--DMDGNGTIDIDEFISATMHRYKLD
AtCPK31           KSGTITLEELKTGLTRLGS-NLSKTEVEQLMEAA--DVDGNGTIDIDEFISATMHRYRLD
AtCPK22           KSGSITYEELKMGLNRHGS-KLSETEVKQLMEAVSADVDGNGTIDYIEFISATMHRHRLE
HvCPK6            NKGQLTFDEFKAGLRKLG-NKMHDSDLQMLMDAA--DVDKNGTLDYGEFVAVSIHVRKIG
OsCPK8            KNGQLTFEDFKAGIRKLG-NQMPDSDLKILMDAA--DIDKNGILDYQEFVAVSIHVRKIG
HvCPK7            KDSMINFDELKLGLNKLG-HQMPDADVQILMDAA--DADGNGCLDYGEFVTLSVHLKKIG
OsCPK20           KDNMINFDELKLGLHKLG-HQMADADVQILMDAA--DVDGNGSLDYGEFVALSVHLRKIG
AtCPK7            KRGKINLEELKYGLQKAG-QQIADTDLQILMEAT--DVDGDGTLNYSEFVAVSVHLKKMA
AtCPK8            KTGKINLEELKFGLHKLGQQQIPDTDLQILMEAA--DVDGDGTLNYGEFVAVSVHLKKMA
AtCPK14           NRGKITITELGIGLQKLG-IVVPQDDIQILMDAG--DVDKDGYLDVNEFVAISVHIRKLG
AtCPK32           QRGKINIDELKIGLQKLG-HAIPQDDLQILMDAG--DIDRDGYLDCDEFIAISVHLRKMG
OsCPK3            NDGIVSYEELKSGIAKFG-SHLAESEVQMLIEAV--DTNGKDALDYGEFLAVSLHLQRMA
OsCPK16           NDGIVSYEELKSGIAKFG-SHLAESEVQMLIEAV--DTNGRGALDYGEFLAVSLHLQRMA
HvCPK10           NDGIVSCEELKSGIAKFG-SHLAESEVQMLIEAV--DTNGKGVLDYAEFLAVSLHLQRMA
AtCPK13           NDGIVSIEELKAGLRDFS-TQLAESEVQMLIEAV--DTKGKGTLDYGEFVAVSLHLQKVA
AtCPK10           KDGKITYPELKAGLQKVG-SQLGEPEIKMLMEVA--DVDGNGFLDYGEFVAVIIHLQKIE
AtCPK30           NDGKISYLELRAGLRKVG-SQLGEPEIKLLMEVA--DVNGNGCLDYGEFVAVIIHLQKME
OsCPK9            NNGRVTLQELKDGLTKVG-SKLAEPEMELLMEAA--DVDGNGYLDYGEFVAVTIHLQRLS
HvCPK29           KDGHLTIEELRKGLQMIG-NNVHDTDVDMLMEAA--DLDGNGTLDCKEFVTVSVHLKKIC
OsCPK29           KKGHLTLEELRKGLQVIG-HNIHDTDVDMLMEAA--DIDGNGILDCKEFVTVSIHLKKIR
HvCPK21           KNGNLSLEELMEGLHING-QRVPESEIRMLLEAA--DTDGNGTLDCDEFVTVSLHLKKMT
OsCPK21           KNGHLSLDELLEGLHING-QPVPEPEIRMLLEAA--DTDGNGTLDCDEFVTVSVHLKKMS
HvCPK22           KDGTLTLEELKEGLRING-HPVPESEIQMLLEAG--DIDGNGTLDTEEFVTVLLHIKKKS
OsCPK22           NSGNLTLEDLKLGLQING-HPVPETEIEMLLEAG--DIDGNGTLDCEEFVTVLLHIKKMS
AtCPK24           KNGHLTFEELRDGLKKIG-QVVPDGDVKMLMDAA--DTDGNGMLSCDEFVTLSIHLKRMG
HvCPK5            KSGSISIEEMRHALAKDLPWRLKGPRVLEIIQAI--DSNTDGLVDFKEFVAATLHIHQMA
OsCPK4            KSGSISIEEMRHALAKDLPWRLKGPRVLEIIQAI--DSNTDGLVDFEEFVAATLHIHQMA
HvCPK11           KSGMISLEELKQALAKDVPWRLKGPRVLEIVEAI--DSNTDGFVDFEEFVAATLHMHQLV
OsCPK18           KNGTISLEELKQALAKDVPWRLKGPRVLEIVEAI--DSNTDGLVDFEEFVAATLHVHQLV
AtCPK16           KNGVISLEEMRQALAKDHPWKLKDARVAEILQAI--DSNTDGFVDFGEFVAAALHVNQLE
AtCPK18           KNGSISLEEMRQALAKDVPWKLKDARVAEILQAN--DSNTDGLVDFTEFVVAALHVNQLE
AtCPK28           KNGVISLEEMRQALAKDLPWKLKDSRVAEILEAI--DSNTDGLVDFTEFVAAALHVHQLE
                    . :.  ::   :       .    :  ::     . .    :   **:   :   :
```

Calmodulin -like domain

```
HvCPK6a           REEH------LMAAFSYFDKDGSGYITVDELEVACRDHNMA-----DVGIDDIIREVDQD
OsCPK6            REEH------LLAAFAYFDRDGSGYITVDELEHACRDHNMA-----DVGIDDIIREVDQD
HvCPK4            REEH------LVAAFSYFDKDGSGYITVDELQQACLEHNMP-----DAFLDDVIKEADQD
OsCPK13           REEH------LVAAFSYFDKDGSGYITVDELQQACKEHNMP-----DAFLDDVINEADQD
HvCPK5a           REEH------LVAAFSYFDKDGSGYITVDELQQACKEHNMP-----DAFLDDVIIEADQD
OsCPK5            REEH------LVAAFSYFDKDGSGYITVDELQQACKEHNMP-----DAFLDDVIKEADQD
AtCPK5            REEH------LVAAFQYFDKDGSGFITIDELQQACVEHGMA-----DVFLEDIIKEVDQN
AtCPK6            REEH------LVSAFQYFDKDGSGYITIDELQQSCIEHGMT-----DVFLEDIIKEVDQD
AtCPK26           REEH------LLSAFRYFDKDGSGYITIDELQHACAEQGMS-----DVFLEDVIKEVDQD
OsCPK7            REEH------LLAAFTYFDKDGSGYITVDKLQRACGEHNME-----DSLLEEIISEVDQN
```

```
OsCPK23         REKH------LMAAFTYFDKDGSGFITVDKLQKACMERNME-----DTFLEEMILEVDQN
HvCPK2          REEN------LVSAFSFFDKDGSGFITIDELSHACREFGLD-----DVHLEDMIKDVDQN
HvCPK24         REEN------LVSAFSFFDKDGSGFITIDELSHACREFGLD-----DVHLEDMIKDVDQN
OsCPK24         REEN------LVSAFSFFDKDGSGFITIDELSQACREFGLD-----DLHLEDMIKDVDQN
HvCPK28         REEN------LVSAFAFFDKDGSGFITIDELSQACEKFGLS-----DVHLEDMIKDVDQN
OsCPK28         REEN------LVSAFTFFDKDGSGFITIDELSQACEQFGLS-----DVHLEDMIKDVDQN
AtCPK4          REEN------LVVAFSYFDKDGSGYITIDELQQACTEFGLC-----DTPLDDMIKEIDLD
AtCPK11         REEN------LVAAFSYFDKDGSGYITIDELQSACTEFGLC-----DTPLDDMIKEIDLD
AtCPK12         REEN------LVAAFSFFDKDASGYITIEELQQAWKEFGIN-----DSNLDEMIKDIDQD
HvCPK17         REDN------LFAAFSYFDKDGSGYITQDELQKACEEFGIG-----DAHLDDIIRDIDQD
OsCPK17         REDN------LFAAFSYFDKDSSGYITQDELQKACEEFGIG-----DAHLEDIIKDIDQD
HvCPK11a        KEDK------LFAAFQYFDKDGSGYITQDELQKACEEFGIG-----DTRIEDIIGDVDKD
OsCPK11         KEDK------LFAAFSYFDKDGSGYITQDELQKACEEFGIG-----DTRIEDIIGDIDQD
HvCPK8          REDH------LFAAFQYFDKDGSGYITPDELQLACEEFGLGG---DDLSLDNMIREVDQD
OsCPK10         REDH------LFAAFQYFDKDGSGYITADELQLACEEFGLG-----DVQLEEMIREVDED
OsCPK27         REDH------LYAAFQYFDKDGSGYITSDELQQACDEFGIE-----DVRLEDMIGEVDQD
AtCPK20         KEDH------LFTAFSYFDQDGSGYITRDELQQACKQFGLA-----DVHLDDILREVDKD
AtCPK1          REDH------LFAAFTYFDKDGSGYITPDELQQACEEFGVE-----DVRIEELMRDVDQD
AtCPK2          REDH------LFAAFSYFDKDESGFITPDELQQACEEFGVE-----DARIEEMMRDVDQD
AtCPK25         EEE-----------------------ANDRLESSTKV-----------------------
HvCPK15         KEDH------IFKAFEYFDKDHSGYITVDELEEALKKYDMGD----EATIKDIIAEVDTD
OsCPK15         KEDH------IYKAFEYFDKDHSGFITVDELEEALTKYDMGD----EATIKEIIAEVDTD
HvCPK1          KEDH------ILKAFEYFDKDHSGYITVDELEEALKKYDMGD----DKTIKDIIAEVDTD
OsCPK1          KEDH------ILKAFEYFDKDHSGYITVDELEEALKKYDMGD----DKTIKEIIAEVDTD
AtCPK3          REDH------LYTAFQFFDNDNSGYITMEELELAMKKYNMGD----DKSIKEIIAEVDTD
HvCPK2a         REEH------LYTAFQYFDKDNSGYISKEELEQALREKGLLE---DGRDIKEIVSEVDAD
OsCPK2          REEH------LYTAFQYFDKDNSGCISKEELEQALREKGLLD----GRDIKDIISEVDAD
HvCPK14         REEH------LYTAFQYFDKDNSGYITIEELEQALREKGLMD----GRDIKDIISEVDAD
OsCPK14         REEH------LYTAFQYFDKDNSGYITIEELEQALREKGLMD----GREIKDIISEVDAD
AtCPK17         REEH------LYSAFQHFDKDNSGYITMEELEQALREFGMND----GRDIKEIISEVDGD
AtCPK34         REEH------LYSAFQHFDKDNSGYITTEELEQALREFGMND----GRDIKEIISEVDGD
OsCPK25         REEH------LYTAFQYFDKDNSGYITKEELEQALKEQGLYD----ANEIKDVITDADSN
OsCPK26         REEH------LYTAFQYFDKDNSGYITKEELEQALKEQGLYD----ANEIKDVITDADSN
HvCPK25         REEH------LYTAFQYFDKDNSGYITKEELEQALQEQKLYD----PEEFKDVIADADSD
HvCPK12         KEED------LLRAFQHFDKDSSGYISRDELEQAMTEYGMGD----EANIKAVLDEVDKD
OsCPK12         KEED------LLRAFQHFDKDNSGYITRDELEQAMAEYGMGD----EANIKQVLDEVDKD
AtCPK29         KEEN------LIEAFKYFDKDRSGFITRDELKHSMTEYGMGD----DATIDEVINDVDTD
HvCPK3          RDEH------LFKAFQYFDKDNSGFITRDELETALIEHEMGD----ADTIKDIISEVDTD
OsCPK19         RDEH------LFKAFQYFDKDNSGFITRDELESALIEHEMGD----TSTIKDIISEVDTD
AtCPK9          SNEN------LYKAFQHFDKDSSGYITIDELESALKEYGMGD----DATIKEVLSDVDSD
AtCPK33         SNEN------VYKAFQHFDKDGSGYITTDELEAALKEYGMGD----DATIKEILSDVDAD
AtCPK21         RDEH------VYKAFQHFDKDNSGHITRDELESAMKEYGMGD----EASIKEVISEVDTD
AtCPK23         HDEH------VHKAFQHLDKDKNGHITRDELESAMKEYGMGD----EASIKEVISEVDTD
AtCPK15         RDEH------VFKAFQYFDKDNSGFITMDELESAMKEYGMGD----EASIKEVIAEVDTD
AtCPK19         REDN------LFKAFQHFDKDNSGFISRQELETAMKEYNMGD----DIMIKEIISEVDAD
AtCPK27         RDEH------VYKAFQHFDKDNDGHITKEELEMAMKEDGAGD----EGSIKQIIADADTD
AtCPK31         RDDH------VYQAFQHFDKDNDGHITKEELEMAMKEHGVGD----EVSIKQIITEVDTD
AtCPK22         RDEH------LYKAFQYFDKDGSGHITKEEVEIAMKEHGMGD----EANAKDLISEFDKN
HvCPK6          NDEH------IQKAFSYFDQDKSGYIEIEELRVALT-DEVDG-PCDEDIINGIIHDVDTD
OsCPK8          NDEH------IQKAFSYFDQNKSGYIEIEELREALV-DEIDG--NDEDIINSIIRDVDTD
HvCPK7          NDEH------LHKAFGYFDRNKSGYIEIDELRESLA-DDLG--PNHEEVINAIIRDVDTD
OsCPK20         NDEH------LHKAFAYFDRNQSGYIEIDELRESLA-DDLG--ANHEEVINAIIRDVDTD
AtCPK7          NDEH------LHKAFNFFDQNQSGYIEIDELREALN-DELDN-TSSEEVIAAIMQDVDTD
AtCPK8          NDEH------LHKAFSFFDQNQSDYIEIEELREALN-DEVD--TNSEEVVAAIMQDVDTD
AtCPK14         NDEH------LKKAFTFFDKNKSGYIEIEELRDALA-DDVD--TTSEEVVEAIILDVDTN
AtCPK32         NDEH------LKKAFAFFDQNNNGYIEIEELREALS-DELG---TSEEVVDAIIRDVDTD
OsCPK3          NDEH------LRRAFLFFDKDGNGYIEPEELREALV-DDGA--GDSMEVVNDILQEVDTD
OsCPK16         NGEH------LRRAFLFFDKDGNGYIEPEELQEALV-EDGA--TDIMEVVKDILQEVDTD
HvCPK10         NDEH------LRRAFLFFDKDGNGYIEPDELREALK-DDGA--ADSMEVVNDILQEVDTD
AtCPK13         NDEH------LRKAFSYFDKDGNGYILPQELCDALK-EDGG--DDCVDVANDIFQEVDTD
AtCPK10         NDEL------FKLAFMFFDKDGSTYIELDELREALA-DELG--EPDASVLSDIMREVDTD
AtCPK30         NDEH------FRQAFMFFDKDGSGYIESEELREALT-DELG--EPDNSVIIDIMREVDTD
OsCPK9          NDNH------LRTAFLFFDKDGSGYIDRAELADALA-DDSG--HADDAVLDHILREVDTD
HvCPK29         SEEH------LPKVFNYFDKNMSGFIEMEELK-----EALSPR-GDQKAVEDIIFDVDID
OsCPK29         SDEH------LPKVFSFFDKNGSGYIEIEELK-----EALSPR-GDQKSIDDIFLDVDID
HvCPK21         NDKY------LAAAFRYFDKDGSGFIEIDELR-----QELGP---NEQAILEIIRDVDTD
OsCPK21         NDEY------LAAAFNYFDKDGSGFIELDELR-----EEVGP---NEQAILEILRDVDTD
HvCPK22         NEEY------LPEAFKYFDKDGNGYIEMEELMEALGDDELGP---DEQVIKDIIRDVDTD
```

```
OsCPK22         NEEY------LPKAFKFFDKDGNGFIEMEELMDALG-DELGP---TEQVVKDIIRDIDTD
AtCPK24         CDEH------LQEAFKYFDKNGNGFIELDELKVALCDDKLGHANGNDQWIKDIFFDVDLN
HvCPK5          ELDSERWGLRCQAAFSKFDLDGDGYITPDELRM---HTGLKG------SIEPLLEEADID
OsCPK4          ELDSERWGLRCQAAFSKFDLDGDGYITPDELRMV-QHTGLKG------SIEPLLEEADID
HvCPK11         EHDAEKWKSLSQAAFDKFDVDGDGYITSNELRM---NTGLKG------SIDPLLEEADID
OsCPK18         EHDTEKWKSLSQAAFDKFDVDGDGYITSDELRM---QTGLKG------SIDPLLEEADID
AtCPK16         EHDSEKWQQRSRAAFEKFDIDGDGFITAEELRM---HTGLKG------SIEPLLEEADID
AtCPK18         EHDSEKWQQRSRAAFDKFDIDGDGFITPEELRL---QTGLKG------SIEPLLEEADVD
AtCPK28         EHDSEKWQLRSRAAFEKFDLDKDGYITPEELRM---HTGLRG------SIDPLLDEADID
                  .                          .:
```

CLD

```
HvCPK6a         NDGRIDYGEFVAMMKKG---IIGNGK------------LTMRHTSDGS-----------V
OsCPK6          NDGRIDYGEFVAMMKKGAIDIIGNGR------------LTIGRPTTAT-----------S
HvCPK4          NDGRIDYGEFVAMMTKG---NMGVGR------------RTMRNSLNIS-----------M
OsCPK13         NDGRIDYGEFVAMMTKG---NMGVGR------------RTMRNSLNIS-----------M
HvCPK5a         NDGRIDYGEFVAMMTKG---NMGVGR------------RTMRNSLNIS-----------M
OsCPK5          NDGRIDYGEFVAMMTKG---NMGVGR------------RTMRNSLNIS-----------M
AtCPK5          NDGKIDYGEFVEMMQKG---NAGVGR------------RTMRNSLNIS-----------M
AtCPK6          NDGRIDYEEFVAMMQKG---NAGVGR------------RTMKNSLNIS-----------M
AtCPK26         NDGRIDYGEFVAMMQKG---IVG---------------RTMRKSINMS-----------I
OsCPK7          NDGQIDYAEFVAMMQGS---NVGLGW------------QTMESSLNVHSLCWLFFQRPVL
OsCPK23         NDGQIDYAEFVTMMQSN---NFGLGW------------QTVESSLNVA-----------L
HvCPK2          NDGQIDYSEFTAMMRKG---NAGAT-----------GRRTMRNSLNLNLGDI-------L
HvCPK24         NDGQIDYSEFTAMMRKG---NAGAT-----------GRRTMRNSLNLNLGDI-------L
OsCPK24         NDGQIDYSEFTAMMRKG---NAGGA-----------GRRTMRNSLQLNLGEI-------L
HvCPK28         NDGQIDYSEFAAMMRKG---NAGGS--SGTSGTVGAGRRTMRNSLHVNLGEI-------L
OsCPK28         NDGQIDYSEFAAMMRKG---NAGGANAGGVTSTGGTGRRTMRNSLRVNLGDI-------L
AtCPK4          NDGKIDFSEFTAMMKKG---DGVGR------------SRTMRNNLNFNIAEA-------F
AtCPK11         NDGKIDFSEFTAMMRKG---DGVGR------------SRTMMKNLNFNIADA-------F
AtCPK12         NDGQIDYGEFVAMMRKG---NGTGG---------GIGRRTMRNSLNFGTTLP-------D
HvCPK17         NDGRIDYNEFVTMMQKG---NNPLGK------------KGQ-GQTSFGRPVS-------R
OsCPK17         NDGRIDYNEFVTMMQKG---NNPLGK------------KGQ-GQLSFGLREA-------L
HvCPK11a        NDGKIDYNEFVEMMQKG---NNPLGR------------KGQQSNVNFGLGDA-------L
OsCPK11         NDGRIDYNEFVEMMQKG---NNAMGK------------MGQHSTGNFGLGEA-------L
HvCPK8          NDGRIDYNEFVAMMQKP---ALGLAKK---------AGAGLESSFSIGFREA-------L
OsCPK10         NDGRIDYNEFVAMMQKP---TMGLPAK---------KSGGLQNSFSIGFREA-------L
OsCPK27         NDGRIDYNEFVAMMQKT---TTGFGK--------------KGGHNFSGFRDA-------L
AtCPK20         NDGRIDYSEFVDMMQ-----DTGFGK--------------------MGLKVS--------
AtCPK1          NDGRIDYNEFVAMMQKG---SITGGP--------------VKMGLEKSFSIA-------L
AtCPK2          KDGRIDYNEFVAMMQKG---SIMGGP--------------VKMGLENSISIS-------L
AtCPK25         ------------------------------------------------------------
HvCPK15         HDGKINYQEFVAMMKNN----------------------SPE-IVPNR-RRLF-------
OsCPK15         HDGRINYQEFVAMMKNN----------------------SPE-IVPNR-RRMF-------
HvCPK1          HDGRINYQEFVAMMRNN----------------------SPE-IVPNR-RRMF-------
OsCPK1          HDGRINYQEFVAMMRNN----------------------NPE-IAPNR-RRMF-------
AtCPK3          RDGKINYEEFVAMMKKG----------------------NPE-LVPNR-RRM--------
HvCPK2a         NDGRIDYSEFVAMMRKG----------------------APEGANPKK-RRDVVL-----
OsCPK2          NDGRIDYSEFAAMMRKG----------------------NPE-ANPKK-RRDVVI-----
HvCPK14         HDGRINYTEFVAMMRKG----------------------APEAANPKK-RRDVVL-----
OsCPK14         NDGRINYTEFVAMMRKG----------------------DPE-ANPKK-RRDVVL-----
AtCPK17         NDGRINYDEFVAMMRKG----------------------NPD-PIPKK-RRELSFK----
AtCPK34         NDGRINYEEFVAMMRKG----------------------NPD-PNPKK-RRELSFK----
OsCPK25         NDGRIDYSEFVAMMRKGS--------------------GCAEATNPKKKRRDLVL-----
OsCPK26         NDGRIDYSEFVAMMRKGS--------------------GCAEATNPKKKRRDLVL-----
HvCPK25         NDGRIDYSEFVAMMRKGT--------------------GGAEPSNPKK-RRDLVLE----
HvCPK12         KDGNIDYEEFVEMMRKGK------------------------------------------
OsCPK12         KDGRIDYEEFVEMMRKGI--------------------QT--------------------
AtCPK29         NDGRINYEEFVAMMRKGT--------------------TDSDPKLIR-------------
HvCPK3          NDGRINYEEFCAMMRGG---------------------MQQPIRLK--------------
OsCPK19         NDGRINYEEFCAMMRGGG--------------------MQQPMRLK--------------
AtCPK9          NDGRINYEEFCAMMRSGN--------------------PQQQQPRLF-------------
AtCPK33         NDGRINYDEFCAMMRSGN--------------------PQ--QPRLF-------------
AtCPK21         NDGRINFEEFCAMMRSGS--------------------TQPQ-GKLLPFH----------
AtCPK23         NDGKINFEEFRAMMRCGT--------------------TQPK-GKQYPFH----------
AtCPK15         NDGRINYEEFCAMMRSGI--------------------TLPQQGKILPPCKRVADLN---
```

```
AtCPK19         NDGSINYQEFCNMMKS-C--------------------SQSHQSKLVQPN----------
AtCPK27         NDGKINFEEFRTMMRTES--------------------SLQPEGELLPIIN---------
AtCPK31         NDGKINFEEFRTMMRSGS--------------------SLQPQRELLPIK----------
AtCPK22         NDGKIDYEEFCTMMRNG---------------------ILQPQGKLLKRLYMNLEELKTG
HvCPK6          KDGKISYDEFAAMMKAGT--------------------DWRKASRQYSRQRFSNLSLKLH
OsCPK8          KDGKISYDEFAVMMKAGT--------------------DWRKASRQYSRQRFSNLSLKLQ
HvCPK7          KDGKISFEEFVAMMKAGT--------------------DWRKASRQYSRERFTSLSLKLQ
OsCPK20         KDGKISYDEFAAMMKAGT--------------------DWRKASRQYSRERFTSLSLKLQ
AtCPK7          KDGRISYEEFVAMMKAGT--------------------DWRKASRQYSRERFNSLSLKLM
AtCPK8          KDGRISYEEFAAMMKAGT--------------------DWRKASRQYSRERFNSLSLKLM
AtCPK14         KDGKISYDEFATMMKTGT--------------------DWRKASRQYSRDLFKCLSLKLM
AtCPK32         KDGRISYEEFVTMMKTGT--------------------DWRKASRQYSRERFNSISLKLM
OsCPK3          KDGKISYDEFVAMMKTGT--------------------DWRKASRHYSRGRFNSLSMKLI
OsCPK16         KDGKISYEEFVAMMKTGT--------------------DWRKASRHYSRGRFNSLSIRLI
HvCPK10         KDGKISYDEFVAMMKTGT--------------------DWRKASRHYSRGRFNSLSMKLV
AtCPK13         KDGRISYEEFAAMMKTGT--------------------DWRKASRHYSRGRFNSLSIKLM
AtCPK10         KDGRINYDEFVTMMKAGT--------------------DWRKASRQYSRERFKSLSINLM
AtCPK30         KDGKINYDEFVVMMKAGT--------------------DWRKASRQYSRERFKSLSLNLM
OsCPK9          KDGRISYEEFVAMMKSGT--------------------DWRKASRQYSRERFKTLSNSLI
HvCPK29         KDGKISYEEFELMMKAGV--------------------DWRNASRQYSRAVFNTLSRKMF
OsCPK29         KDGKISYEEFELMMSAGM--------------------DWRNASRQYSRAVYNTLSRKIF
HvCPK21         RDGRISYQEFELMMKSGA--------------------DWRNASRQFSRANFNTLSRKLC
OsCPK21         KDGRISYQEFELMMKSGA--------------------DWRNASRHFSRANFSTLSRRLC
HvCPK22         EDGRISYQEFEVMMRSGS--------------------DWRNASRRYSRANFSNLSHRLC
OsCPK22         KDGRISYQEFESMMISGS--------------------DWRNASRRYSKANFSSLSRKLC
AtCPK24         KDGRISFDEFKAMMKSGT--------------------DWKMASRQYSRALLNALSIKMF
HvCPK5          KDGRISLSEFRKLLRTAS-------------------MSNLPSPSGVPNPQAL-------
OsCPK4          KDGRISLSEFRKLLRTAS-------------------MSNLPSPRGPPNPQPL-------
HvCPK11         KDGKISLDEFRKLLKTAS-------------------MRSCN-PTPRSVSK---------
OsCPK18         RDGKISLDEFRRLLKTAS-------------------MSSRNVQTPRSVHRS--------
AtCPK16         NDGKISLQEFRRLLRTAS-------------------IKSRNVRSPPGYLISRKV-----
AtCPK18         EDGRISINEFRRLLRSAS-------------------LKSKNVKSPPGYQLSQKM-----
AtCPK28         RDGKISLHEFRRLLRTAS-------------------ISSQRAPSPAGHRNLR-------


HvCPK6a         LHGAGSSVSS--------------------------------------------------
OsCPK6          DDPSPTISSSSR------------------------------------------------
HvCPK4          RDAPGAI-----------------------------------------------------
OsCPK13         RDAPGAL-----------------------------------------------------
HvCPK5a         TE----------------------------------------------------------
OsCPK5          R-----------------------------------------------------------
AtCPK5          RDA---------------------------------------------------------
AtCPK6          RDV---------------------------------------------------------
AtCPK26         RNNAVSQ-----------------------------------------------------
OsCPK7          RNESRHVCVTLISMLAFL------------------------------------------
OsCPK23         R-EAPQVY----------------------------------------------------
HvCPK2          NPSNS-------------------------------------------------------
HvCPK24         NPSNS-------------------------------------------------------
OsCPK24         NPSNS-------------------------------------------------------
HvCPK28         RPGGTT------------------------------------------------------
OsCPK28         KPNEN-------------------------------------------------------
AtCPK4          GVEDTSSTAKSDDSPK--------------------------------------------
AtCPK11         GVDGE----KSDD-----------------------------------------------
AtCPK12         ESMNV-------------------------------------------------------
HvCPK17         Q-----------------------------------------------------------
OsCPK17         KLG---------------------------------------------------------
HvCPK11a        KLR---------------------------------------------------------
OsCPK11         KLR---------------------------------------------------------
HvCPK8          RMA---------------------------------------------------------
OsCPK10         RMS---------------------------------------------------------
OsCPK27         KSHS--------------------------------------------------------
AtCPK20         ------------------------------------------------------------
AtCPK1          KL----------------------------------------------------------
AtCPK2          KH----------------------------------------------------------
AtCPK25         ------------------------------------------------------------
HvCPK15         ------------------------------------------------------------
OsCPK15         ------------------------------------------------------------
HvCPK1          ------------------------------------------------------------
```

```
OsCPK1          ------------------------------------------------------------
AtCPK3          ------------------------------------------------------------
HvCPK2a         ------------------------------------------------------------
OsCPK2          ------------------------------------------------------------
HvCPK14         ------------------------------------------------------------
OsCPK14         ------------------------------------------------------------
AtCPK17         ------------------------------------------------------------
AtCPK34         ------------------------------------------------------------
OsCPK25         ------------------------------------------------------------
OsCPK26         ------------------------------------------------------------
HvCPK25         ------------------------------------------------------------
HvCPK12         ------------------------------------------------------------
OsCPK12         ------------------------------------------------------------
AtCPK29         ------------------------------------------------------------
HvCPK3          ------------------------------------------------------------
OsCPK19         ------------------------------------------------------------
AtCPK9          ------------------------------------------------------------
AtCPK33         ------------------------------------------------------------
AtCPK21         ------------------------------------------------------------
AtCPK23         ------------------------------------------------------------
AtCPK15         ------------------------------------------------------------
AtCPK19         ------------------------------------------------------------
AtCPK27         ------------------------------------------------------------
AtCPK31         ------------------------------------------------------------
AtCPK22         LTRLGSRLSETEIDKAFQHFDKDNSGHITRDELESAMKEYGMGDEASIKEVISEVDTDNV
HvCPK6          KDGSISDDQK--------------------------------------------------
OsCPK8          KDGSISDDTQ--------------------------------------------------
HvCPK7          KDGSLQITSTQ-------------------------------------------------
OsCPK20         KDGSLQLTTTQ-------------------------------------------------
AtCPK7          RDGSLQLEGET-------------------------------------------------
AtCPK8          REGSLQLEGEN-------------------------------------------------
AtCPK14         QDGSLQSNGDTK------------------------------------------------
AtCPK32         QDASLQVNGDTR------------------------------------------------
OsCPK3          KDGSVKLVNE--------------------------------------------------
OsCPK16         KDGSVKLGNE--------------------------------------------------
HvCPK10         KDGSVKLGVE--------------------------------------------------
AtCPK13         KDGSLNLGNE--------------------------------------------------
AtCPK10         KDGSLHLHDALTGQTVPV------------------------------------------
AtCPK30         KDGSMHLHDALTGQSIAV------------------------------------------
OsCPK9          KDGSITMAR---------------------------------------------------
HvCPK29         KDVSLKLDPSSPLGGVGKGQRDVI------------------------------------
OsCPK29         KEVSLKLDHSGPLVAAGK------------------------------------------
HvCPK21         KQENSSS-----------------------------------------------------
OsCPK21         KDTLTP------------------------------------------------------
HvCPK22         Q-----------------------------------------------------------
OsCPK22         KGNS--------------------------------------------------------
AtCPK24         KEDFGDNGPKSHSMEFPIARKRAKLLDAPKNKSMELQISKTYKPSGLRN-----------
HvCPK5          ------------------------------------------------------------
OsCPK4          ------------------------------------------------------------
HvCPK11         ------------------------------------------------------------
OsCPK18         ------------------------------------------------------------
AtCPK16         ------------------------------------------------------------
AtCPK18         ------------------------------------------------------------
AtCPK28         ------------------------------------------------------------


HvCPK6a         --------------------
OsCPK6          --------------------
HvCPK4          --------------------
OsCPK13         --------------------
HvCPK5a         --------------------
OsCPK5          --------------------
AtCPK5          --------------------
AtCPK6          --------------------
AtCPK26         --------------------
OsCPK7          --------------------
OsCPK23         --------------------
HvCPK2          --------------------
```

```
HvCPK24     --------------------
OsCPK24     --------------------
HvCPK28     --------------------
OsCPK28     --------------------
AtCPK4      --------------------
AtCPK11     --------------------
AtCPK12     --------------------
HvCPK17     --------------------
OsCPK17     --------------------
HvCPK11a    --------------------
OsCPK11     --------------------
HvCPK8      --------------------
OsCPK10     --------------------
OsCPK27     --------------------
AtCPK20     --------------------
AtCPK1      --------------------
AtCPK2      --------------------
AtCPK25     --------------------
HvCPK15     --------------------
OsCPK15     --------------------
HvCPK1      --------------------
OsCPK1      --------------------
AtCPK3      --------------------
HvCPK2a     --------------------
OsCPK2      --------------------
HvCPK14     --------------------
OsCPK14     --------------------
AtCPK17     --------------------
AtCPK34     --------------------
OsCPK25     --------------------
OsCPK26     --------------------
HvCPK25     --------------------
HvCPK12     --------------------
OsCPK12     --------------------
AtCPK29     --------------------
HvCPK3      --------------------
OsCPK19     --------------------
AtCPK9      --------------------
AtCPK33     --------------------
AtCPK21     --------------------
AtCPK23     --------------------
AtCPK15     --------------------
AtCPK19     --------------------
AtCPK27     --------------------
AtCPK31     --------------------
AtCPK22     SCTLQHIANISNIKQVLETL
HvCPK6      --------------------
OsCPK8      --------------------
HvCPK7      --------------------
OsCPK20     --------------------
AtCPK7      --------------------
AtCPK8      --------------------
AtCPK14     --------------------
AtCPK32     --------------------
OsCPK3      --------------------
OsCPK16     --------------------
HvCPK10     --------------------
AtCPK13     --------------------
AtCPK10     --------------------
AtCPK30     --------------------
OsCPK9      --------------------
HvCPK29     --------------------
OsCPK29     --------------------
HvCPK21     --------------------
OsCPK21     --------------------
HvCPK22     --------------------
OsCPK22     --------------------
AtCPK24     --------------------
```

```
HvCPK5          --------------------
OsCPK4          --------------------
HvCPK11         --------------------
OsCPK18         --------------------
AtCPK16         --------------------
AtCPK18         --------------------
AtCPK28         --------------------
```
