# Supplementary material for: A Role for Barley Calcium-Dependent Protein Kinase CPK2a in the Response to Drought
Source: Front Plant Sci. 2016 Oct 25;7:1550. doi: 10.3389/fpls.2016.01550 (PMC5078816; doi:10.3389/fpls.2016.01550)
Supplement: Data Sheet 2 — MS/MS spectra of phosphopeptides in Table 1. [file DataSheet2.PDF]

MS/MS spectrum for the peptide including Tyr-79 from recombinant His-HvCPK2a obtained after trypsin digestion.

*Hv*CPK2a T79: R.GQFGVTHLC**pT**QK.A
The predominant b and y fragment ions are identified end the neutral mass losses of phosphate (-98 Da) are noted. MS/MS spectrum of GQFGVTHLC**T**QK peptide shows phosphorylation of Tyr-79
Matches : 14/288 fragment ions using 13 most intense peaks

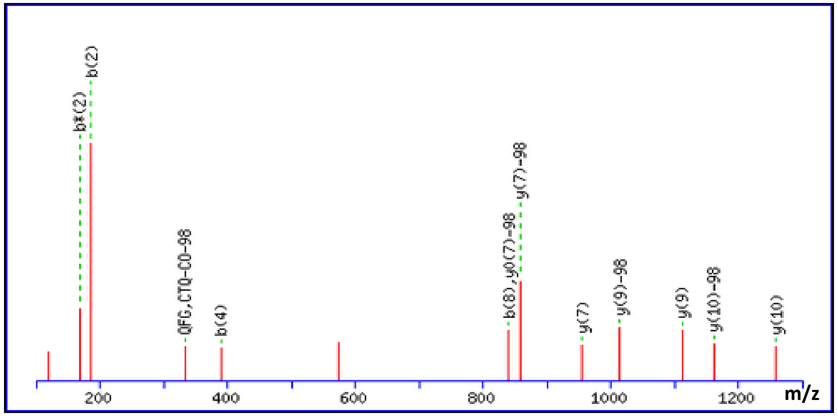

Observed	Mr(expt)	Mr(calc)	ppm	Score	Expect	Rank	Unique
722.8186	1443.6226	1443.6040	12.9	37	0.024	1	-

#	a	a^{++}	b	b^{++}	b*	b^{*++}	b^0	b^{0++}	Seq.	y	y^{++}	y*	y^{*++}	y^0	y^{0++}	#
1	30.0338	15.5206	58.0287	29.5180					G							12
2	158.0924	79.5498	*186.0873*	93.5473	169.0608	85.0340			Q	1387.5898	694.2985	1370.5633	685.7853	1369.5793	685.2933	11
3	305.1608	153.0840	*333.1557*	167.0815	316.1292	158.5682			F	*1259.5312*	630.2693	1242.5047	621.7560	1241.5207	621.2640	10
4	362.1823	181.5948	*390.1772*	195.5922	373.1506	187.0790			G	*1112.4628*	556.7351	1095.4363	548.2218	1094.4523	547.7298	9
5	461.2507	231.1290	489.2456	245.1264	472.2191	236.6132			V	1055.4414	528.2243	1038.4148	519.7110	1037.4308	519.2190	8
6	562.2984	281.6528	590.2933	295.6503	573.2667	287.1370	572.2827	286.6450	T	*956.3730*	478.6901	939.3464	470.1768	938.3624	469.6848	7
7	699.3573	350.1823	727.3522	364.1797	710.3257	355.6665	709.3416	355.1745	H	855.3253	428.1663	838.2987	419.6530	837.3147	419.1610	6
8	812.4414	406.7243	*840.4363*	420.7218	823.4097	412.2085	822.4257	411.7165	L	718.2664	359.6368	701.2398	351.1235	700.2558	350.6315	5
9	961.4383	481.2228	989.4332	495.2202	972.4066	486.7069	971.4226	486.2149	C	605.1823	303.0948	588.1557	294.5815	587.1717	294.0895	4
10	1142.4523	571.7298	1170.4472	585.7272	1153.4206	577.2140	1152.4366	576.7219	T	456.1854	228.5963	439.1588	220.0831	438.1748	219.5911	3
11	1270.5108	635.7591	1298.5058	649.7565	1281.4792	641.2432	1280.4952	640.7512	Q	275.1714	138.0893	258.1448	129.5761			2
12									K	147.1128	74.0600	130.0863	65.5468			1

MS/MS spectrum for the peptide including Tyr-83 and Lys-85 from recombinant His-HvCPK2a obtained after trypsin digestion.

*Hv*CPK2a T83, K85: : R.GQFGVTYLC**pT**E**ubqK**.S

The predominant b and y fragment ions are identified end the neutral mass losses of phosphate (-98 Da) are noted. For ubiquitination site characteristic grup GlyGly was observed.

MS/MS spectrum of GQFGVTYLC**T**E**K** peptide shows phosphorylation of Tyr-83 and ubiquitination Lys-85

Matches : 21/294 fragment ions using 26 most intense peaks

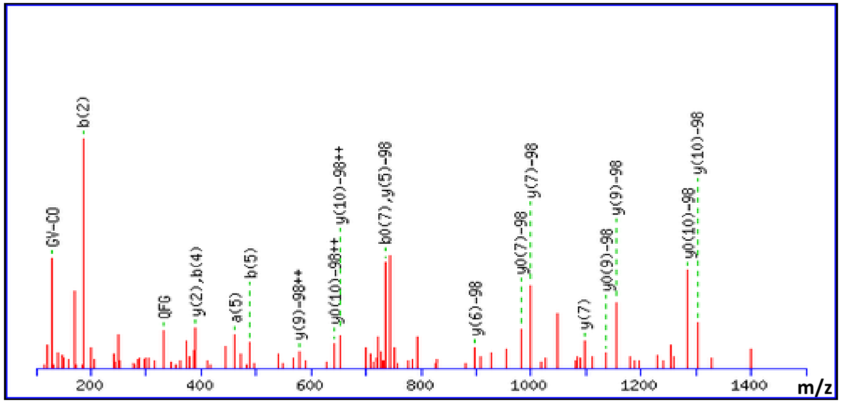

Observed	Mr(expt)	Mr(calc)	ppm	Score	Expect	Rank	Unique
793.3122	1584.6098	1584.6354	-16.1	37	0.017	1	U

#	a	a++	b	b++	b*	b*++	b0	b0++	Seq.	y	y++	y*	y*++	y0	y0++	#
1	30.0338	15.5206	58.0287	29.5180					G							12
2	158.0924	79.5498	**186.0873**	93.5473	169.0608	85.0340			Q	1430.6443	715.8258	1413.6177	707.3125	1412.6337	706.8205	11
3	305.1608	153.0840	**333.1557**	167.0815	316.1292	158.5682			F	***1302.5857***	651.7965	1285.5592	643.2832	1284.5751	642.7912	10
4	362.1823	181.5948	**390.1772**	195.5922	373.1506	187.0790			G	***1155.5173***	578.2623	1138.4907	569.7490	1137.5067	569.2570	9
5	**461.2507**	231.1290	**489.2456**	245.1264	472.2191	236.6132			V	1098.4958	549.7516	1081.4693	541.2383	1080.4853	540.7463	8
6	562.2984	281.6528	590.2933	295.6503	573.2667	287.1370	572.2827	286.6450	T	***999.4274***	500.2173	982.4009	491.7041	981.4169	491.2121	7
7	725.3617	363.1845	753.3566	377.1819	736.3301	368.6687	735.3461	368.1767	Y	***898.3797***	449.6935	881.3532	441.1802	880.3692	440.6882	6
8	838.4458	419.7265	866.4407	433.7240	849.4141	425.2107	848.4301	424.7187	L	***735.3164***	368.1618	718.2899	359.6486	717.3058	359.1566	5
9	987.4427	494.2250	1015.4376	508.2224	998.4110	499.7092	997.4270	499.2171	C	622.2323	311.6198	605.2058	303.1065	604.2218	302.6145	4
10	1070.4798	535.7435	1098.4747	549.7410	1081.4482	541.2277	1080.4641	540.7357	T	473.2354	237.1214	456.2089	228.6081	455.2249	228.1161	3
11	1199.5224	600.2648	1227.5173	614.2623	1210.4907	605.7490	1209.5067	605.2570	E	***390.1983***	195.6028	373.1718	187.0895	372.1878	186.5975	2
12									K	261.1557	131.0815	244.1292	122.5682			1

MS/MS spectrum for the peptide including Tyr-95 from recombinant His-HvCPK2a obtained after trypsin digestion and TiO_2 enrichment.

*Hv*CPK2a T95: Q.FACK**pT**IAK.R

The predominant b and y fragment ions are identified end the neutral mass losses of phosphate (-98 Da) are noted. MS/MS spectrum of FACK**T**IAK peptide shows phosphorylation of Tyr-95

Matches : 33/178 fragment ions using 30 most intense peaks

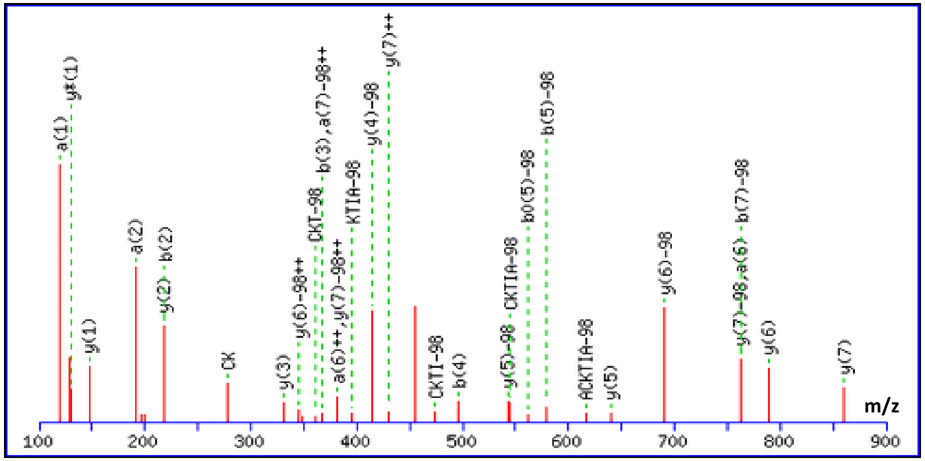

Observed	Mr(expt)	Mr(calc)	ppm	Score	Expect	Rank	Unique
504.2228	1006.4310	1006.4381	-7.03	57	0.0041	1	-

#	a	a^{++}	b	b^{++}	b^{*}	b^{*++}	b^{0}	b^{0++}	Seq.	y	y^{++}	y^{*}	y^{*++}	y^{0}	y^{0++}	#
1	120.0808	60.5440	148.0757	74.5415					F							8
2	191.1179	96.0626	219.1128	110.0600					A	762.4001	381.7037	745.3735	373.1904	744.3895	372.6984	7
3	340.1148	170.5610	368.1097	184.5585					C	691.3630	346.1851	674.3364	337.6718	673.3524	337.1798	6
4	468.2098	234.6085	496.2047	248.6060	479.1781	240.0927			K	542.3661	271.6867	525.3395	263.1734	524.3555	262.6814	5
5	551.2469	276.1271	579.2418	290.1245	562.2152	281.6113	561.2312	281.1192	T	414.2711	207.6392	397.2445	199.1259	396.2605	198.6339	4
6	664.3309	332.6691	692.3259	346.6666	675.2993	338.1533	674.3153	337.6613	I	331.2340	166.1206	314.2074	157.6074			3
7	735.3681	368.1877	763.3630	382.1851	746.3364	373.6718	745.3524	373.1798	A	218.1499	109.5786	201.1234	101.0653			2
8									K	147.1128	74.0600	130.0863	65.5468			1

MS/MS spectrum for the peptide including Ser-102 from recombinant His-HvCPK2a obtained after trypsin digestion and TiO_2 enrichment.

*Hv*CPK2a Ser-102: R.KLI**pS**KEDVEDVR.R

The predominant b and y fragment ions are identified end the neutral mass losses of phosphate (-98 Da) are noted. MS/MS spectrum of KLI**S**KEDVEDVR peptide shows phosphorylation of Ser-102

Matches : 20/322 fragment ions using 36 most intense peaks

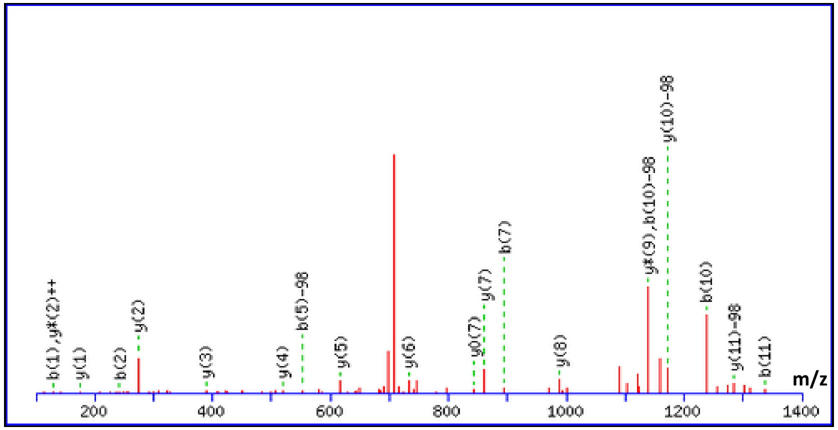

Observed	Mr(expt)	Mr(calc)	ppm	Score	Expect	Rank	Unique
755.8621	1509.7096	1509.7440	-22.8	66	0.0011	1	-

#	a	a^{++}	b	b^{++}	b^{*}	b^{*++}	b^{0}	b^{0++}	Seq.	y	y^{++}	y^{*}	y^{*++}	y^{0}	y^{0++}	#
1	101.1073	51.0573	**129.1022**	65.0548	112.0757	56.5415			K							12
2	214.1914	107.5993	**242.1863**	121.5968	225.1598	113.0835			L	*1284.6794*	642.8433	1267.6529	634.3301	1266.6688	633.8381	11
3	327.2755	164.1414	355.2704	178.1388	338.2438	169.6255			I	*1171.5953*	586.3013	1154.5688	577.7880	1153.5848	577.2960	10
4	396.2969	198.6521	424.2918	212.6496	407.2653	204.1363	406.2813	203.6443	S	1058.5113	529.7593	1041.4847	521.2460	1040.5007	520.7540	9
5	524.3919	262.6996	**552.3868**	276.6970	535.3602	268.1838	534.3762	267.6918	K	*989.4898*	495.2485	972.4633	486.7353	971.4793	486.2433	8
6	653.4345	327.2209	681.4294	341.2183	664.4028	332.7051	663.4188	332.2130	E	*861.3949*	431.2011	844.3683	422.6878	843.3843	422.1958	7
7	768.4614	384.7343	796.4563	398.7318	779.4298	390.2185	778.4458	389.7265	D	*732.3523*	366.6798	715.3257	358.1665	714.3417	357.6745	6
8	867.5298	434.2686	895.5247	448.2660	878.4982	439.7527	877.5142	439.2607	V	*617.3253*	309.1663	600.2988	300.6530	599.3148	300.1610	5
9	996.5724	498.7898	1024.5673	512.7873	1007.5408	504.2740	1006.5568	503.7820	E	*518.2569*	259.6321	501.2304	251.1188	500.2463	250.6268	4
10	1111.5994	556.3033	**1139.5943**	570.3008	1122.5677	561.7875	1121.5837	561.2955	D	*389.2143*	195.1108	372.1878	186.5975	371.2037	186.1055	3
11	1210.6678	605.8375	1238.6627	619.8350	1221.6361	611.3217	1220.6521	610.8297	V	*274.1874*	137.5973	257.1608	129.0840			2
12									R	*175.1190*	88.0631	158.0924	79.5498			1

MS/MS spectrum for the peptide including Ser-199 from recombinant His-HvCPK2a obtained after trypsin digestion and TiO_2 enrichment.

*Hv*CPK2a Ser-199: R.DLKPENFLLLpSK.D
The predominant b and y fragment ions are identified end the neutral mass losses of phosphate (-98 Da) are noted. MS/MS spectrum of DLKPENFLLLSK peptide shows phosphorylation of Ser-199
Matches : 63/289 fragment ions using 63 most intense peaks

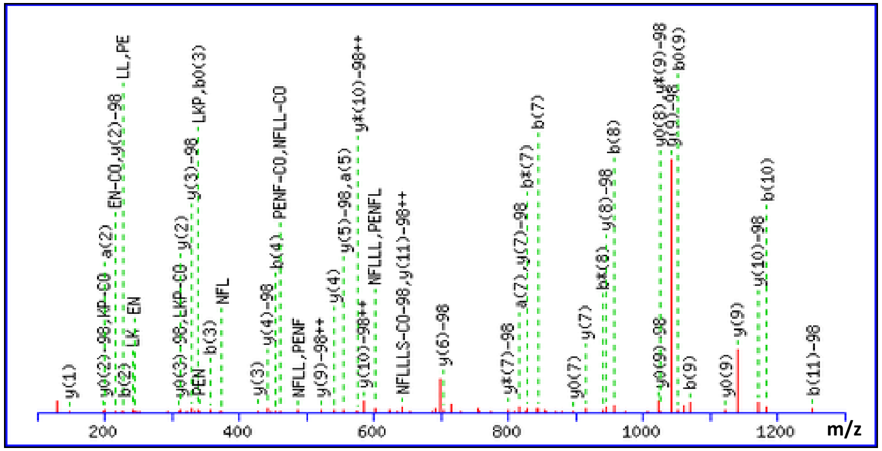

Observed	Mr(expt)	Mr(calc)	ppm	Score	Expect	Rank	Unique
748.8919	1495.7693	1495.7687	0.38	71	0.00027	1	U

#	a	a++	b	b++	b*	b*++	b0	b0++	Seq.	y	y++	y*	y*++	y0	y0++	#
1	88.0393	44.5233	116.0342	58.5207			98.0237	49.5155	D							12
2	**201.1234**	101.0653	**229.1183**	115.0628			211.1077	106.0575	L	1283.7722	642.3897	1266.7456	633.8765	1265.7616	633.3844	11
3	**329.2183**	165.1128	**357.2132**	179.1103	340.1867	170.5970	339.2027	170.1050	K	***1170.6881***	585.8477	1153.6616	577.3344	1152.6776	576.8424	10
4	426.2711	213.6392	**454.2660**	227.6366	437.2395	219.1234	436.2554	218.6314	P	***1042.5932***	521.8002	1025.5666	513.2869	1024.5826	512.7949	9
5	**555.3137**	278.1605	583.3086	292.1579	566.2821	283.6447	565.2980	283.1527	E	***945.5404***	473.2738	928.5138	464.7606	927.5298	464.2686	8
6	669.3566	335.1819	697.3515	349.1794	680.3250	340.6661	679.3410	340.1741	N	***816.4978***	408.7525	799.4713	400.2393	798.4872	399.7473	7
7	**816.4250**	408.7162	**844.4199**	422.7136	827.3934	414.2003	826.4094	413.7083	F	***702.4549***	351.7311	685.4283	343.2178	684.4443	342.7258	6
8	929.5091	465.2582	**957.5040**	479.2556	940.4775	470.7424	939.4934	470.2504	L	***555.3865***	278.1969	538.3599	269.6836	537.3759	269.1916	5
9	**1042.5932**	521.8002	**1070.5881**	535.7977	1053.5615	527.2844	1052.5775	526.7924	L	***442.3024***	221.6548	425.2758	213.1416	424.2918	212.6496	4
10	1155.6772	578.3422	**1183.6721**	592.3397	1166.6456	583.8264	1165.6616	583.3344	L	***329.2183***	165.1128	312.1918	156.5995	311.2078	156.1075	3
11	1224.6987	612.8530	**1252.6936**	626.8504	1235.6671	618.3372	1234.6830	617.8452	S	***216.1343***	108.5708	199.1077	100.0575	198.1237	99.5655	2
12									K	***147.1128***	74.0600	130.0863	65.5468			1

MS/MS spectrum for the peptide including Ser-214 from recombinant His-HvCPK2a obtained after trypsin digestion and TiO_2 enrichment.

*Hv*CPK2a Ser-214: K.ATDFGL**pS**VFFK.P
The predominant b and y fragment ions are identified end the neutral mass losses of phosphate (-98 Da) are noted. MS/MS spectrum of ATDFGL**S**VFFK peptide shows phosphorylation of Ser-214
Matches : 20/266fragment ions using 20 most intense peaks

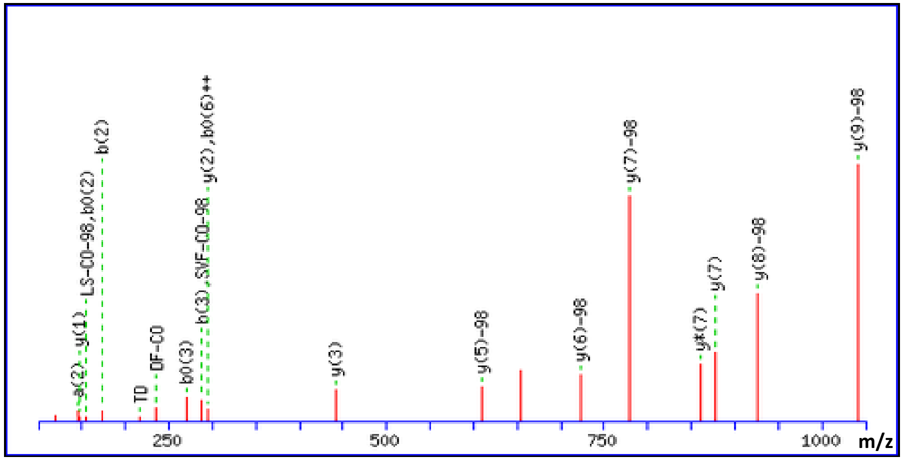

Observed	Mr(expt)	Mr(calc)	ppm	Score	Expect	Rank	Unique
656.3063	1310.5981	1310.5948	2.51	77	9.1e-05	1	U

#	a	a^{++}	b	b^{++}	b^{0}	b^{0++}	Seq.	y	y^{++}	y^{*}	y^{*++}	y^{0}	y^{0++}	#
1	44.0495	22.5284	72.0444	36.5258			A							11
2	145.0972	73.0522	173.0921	87.0497	155.0815	78.0444	T	1142.5881	571.7977	1125.5615	563.2844	1124.5775	562.7924	10
3	260.1241	130.5657	288.1190	144.5631	270.1084	135.5579	D	1041.5404	521.2738	1024.5138	512.7606	1023.5298	512.2686	9
4	407.1925	204.0999	435.1874	218.0974	417.1769	209.0921	F	926.5135	463.7604	909.4869	455.2471	908.5029	454.7551	8
5	464.2140	232.6106	492.2089	246.6081	474.1983	237.6028	G	779.4450	390.2262	762.4185	381.7129	761.4345	381.2209	7
6	577.2980	289.1527	605.2930	303.1501	587.2824	294.1448	L	722.4236	361.7154	705.3970	353.2021	704.4130	352.7101	6
7	646.3195	323.6634	674.3144	337.6608	656.3039	328.6556	S	609.3395	305.1734	592.3130	296.6601	591.3289	296.1681	5
8	745.3879	373.1976	773.3828	387.1951	755.3723	378.1898	V	540.3180	270.6627	523.2915	262.1494			4
9	892.4563	446.7318	920.4512	460.7293	902.4407	451.7240	F	441.2496	221.1285	424.2231	212.6152			3
10	1039.5247	520.2660	1067.5197	534.2635	1049.5091	525.2582	F	294.1812	147.5942	277.1547	139.0810			2
11							K	147.1128	74.0600	130.0863	65.5468			1

MS/MS spectrum for the peptide including Ser-229 from recombinant His-HvCPK2a obtained after trypsin digestion and TiO_2 enrichment.

*Hv*CPK2a Ser-229: K.DIVG**pS**AYYIAPEVLK.R
The predominant b and y fragment ions are identified end the neutral mass losses of phosphate (-98 Da) are noted. MS/MS spectrum of DIVG**S**AYYIAPEVLK peptide shows phosphorylation of Ser-229
Matches : 82/380 fragment ions using 71 most intense peaks

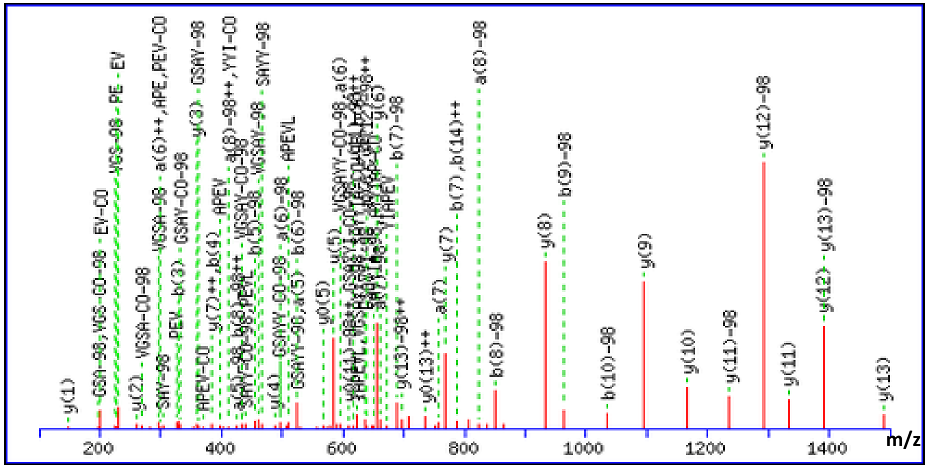

Observed	Mr(expt)	Mr(calc)	ppm	Score	Expect	Rank	Unique
859.4178	1716.8211	1716.8375	-9.57	114	2.3e-08	1	U

#	a	a^{++}	b	b^{++}	b^{0}	b^{0++}	Seq.	y	y^{++}	y^{*}	y^{*++}	y^{0}	y^{0++}	#
1	88.0393	44.5233	116.0342	58.5207	98.0237	49.5155	D							15
2	201.1234	101.0653	229.1183	115.0628	211.1077	106.0575	I	1504.8410	752.9241	1487.8144	744.4109	1486.8304	743.9189	14
3	300.1918	150.5995	328.1867	164.5970	310.1761	155.5917	V	1391.7569	696.3821	1374.7304	687.8688	1373.7464	687.3768	13
4	357.2132	179.1103	385.2082	193.1077	367.1976	184.1024	G	1292.6885	646.8479	1275.6620	638.3346	1274.6780	637.8426	12
5	426.2347	213.6210	454.2296	227.6184	436.2191	218.6132	S	1235.6671	618.3372	1218.6405	609.8239	1217.6565	609.3319	11
6	497.2718	249.1395	525.2667	263.1370	507.2562	254.1317	A	1166.6456	583.8264	1149.6190	575.3132	1148.6350	574.8211	10
7	660.3352	330.6712	688.3301	344.6687	670.3195	335.6634	Y	1095.6085	548.3079	1078.5819	539.7946	1077.5979	539.3026	9
8	823.3985	412.2029	851.3934	426.2003	833.3828	417.1951	Y	932.5451	466.7762	915.5186	458.2629	914.5346	457.7709	8
9	936.4825	468.7449	964.4775	482.7424	946.4669	473.7371	I	769.4818	385.2445	752.4553	376.7313	751.4713	376.2393	7
10	1007.5197	504.2635	1035.5146	518.2609	1017.5040	509.2556	A	656.3978	328.7025	639.3712	320.1892	638.3872	319.6972	6
11	1104.5724	552.7898	1132.5673	566.7873	1114.5568	557.7820	P	585.3606	293.1840	568.3341	284.6707	567.3501	284.1787	5
12	1233.6150	617.3111	1261.6099	631.3086	1243.5994	622.3033	E	488.3079	244.6576	471.2813	236.1443	470.2973	235.6523	4
13	1332.6834	666.8454	1360.6783	680.8428	1342.6678	671.8375	V	359.2653	180.1363	342.2387	171.6230			3
14	1445.7675	723.3874	1473.7624	737.3848	1455.7518	728.3796	L	260.1969	130.6021	243.1703	122.0888			2
15							K	147.1128	74.0600	130.0863	65.5468			1

MS/MS spectrum for the peptide including Tyr-231 from recombinant His-HvCPK2a obtained after trypsin digestion and TiO_2 enrichment.

*Hv*CPK2a Tyr-231: K.DIVGSA**pY**YIAPEVLK.R

The predominant b and y fragment ions are identified end the neutral mass losses of phosphate (-98 Da) are noted. MS/MS spectrum of DIVGSA**Y**YIAPEVLK peptide shows phosphorylation of Tyr-231

Matches : 24/253 fragment ions using 25 most intense peaks

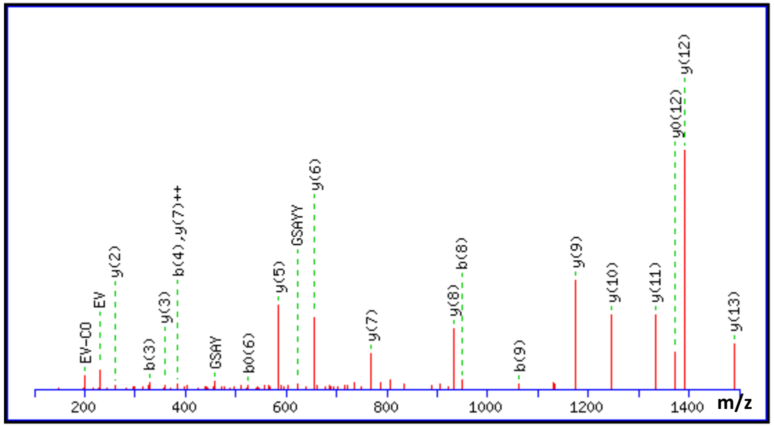

Observed	Mr(expt)	Mr(calc)	ppm	Score	Expect	Rank	Unique
859.4266	1716.8386	1716.8375	0.64	106	1.5e-07	1	U

#	a	a^{++}	b	b^{++}	b^{0}	b^{0++}	Seq.	y	y^{++}	y^{*}	y^{*++}	y^{0}	y^{0++}	#
1	88.0393	44.5233	116.0342	58.5207	98.0237	49.5155	D							15
2	201.1234	101.0653	229.1183	115.0628	211.1077	106.0575	I	1602.8179	801.9126	1585.7913	793.3993	1584.8073	792.9073	14
3	300.1918	150.5995	328.1867	164.5970	310.1761	155.5917	V	1489.7338	745.3706	1472.7073	736.8573	1471.7233	736.3653	13
4	357.2132	179.1103	385.2082	193.1077	367.1976	184.1024	G	1390.6654	695.8363	1373.6389	687.3231	1372.6548	686.8311	12
5	444.2453	222.6263	472.2402	236.6237	454.2296	227.6184	S	1333.6439	667.3256	1316.6174	658.8123	1315.6334	658.3203	11
6	515.2824	258.1448	543.2773	272.1423	525.2667	263.1370	A	1246.6119	623.8096	1229.5854	615.2963	1228.6014	614.8043	10
7	758.3120	379.6597	786.3070	393.6571	768.2964	384.6518	Y	1175.5748	588.2910	1158.5483	579.7778	1157.5642	579.2858	9
8	921.3754	461.1913	949.3703	475.1888	931.3597	466.1835	Y	932.5451	466.7762	915.5186	458.2629	914.5346	457.7709	8
9	1034.4594	517.7334	1062.4544	531.7308	1044.4438	522.7255	I	769.4818	385.2445	752.4553	376.7313	751.4713	376.2393	7
10	1105.4966	553.2519	1133.4915	567.2494	1115.4809	558.2441	A	656.3978	328.7025	639.3712	320.1892	638.3872	319.6972	6
11	1202.5493	601.7783	1230.5442	615.7758	1212.5337	606.7705	P	585.3606	293.1840	568.3341	284.6707	567.3501	284.1787	5
12	1331.5919	666.2996	1359.5868	680.2971	1341.5763	671.2918	E	488.3079	244.6576	471.2813	236.1443	470.2973	235.6523	4
13	1430.6603	715.8338	1458.6552	729.8313	1440.6447	720.8260	V	359.2653	180.1363	342.2387	171.6230			3
14	1543.7444	772.3758	1571.7393	786.3733	1553.7287	777.3680	L	260.1969	130.6021	243.1703	122.0888			2
15							K	147.1128	74.0600	130.0863	65.5468			1

MS/MS spectrum for the peptide including Ser-484 from recombinant His-HvCPK2a obtained after trypsin digestion and TiO_2 enrichment.

*Hv*CPK2a Ser-484: K.EIL**pS**DVDADNDGR.I
The predominant b and y fragment ions are identified end the neutral mass losses of phosphate (-98 Da) are noted. MS/MS spectrum of EIL**S**DVDADNDGR peptide shows phosphorylation of Ser-484
Matches : 32/346 fragment ions using 38 most intense peaks

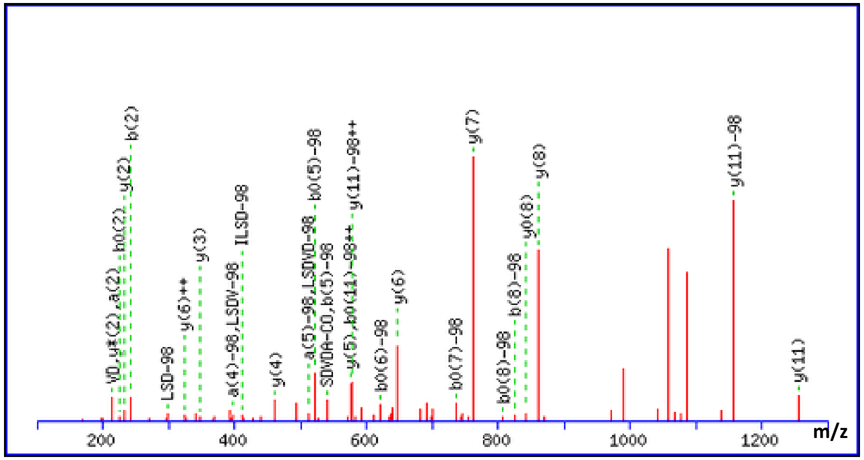

Observed	Mr(expt)	Mr(calc)	ppm	Score	Expect	Rank	Unique
749.8043	1497.5940	1497.5984	-2.93	55	0.013	1	U

#	a	a^{++}	b	b^{++}	b*	b^{*++}	b^0	b^{0++}	Seq.	y	y^{++}	y*	y^{*++}	y^0	y^{0++}	#
1	102.0550	51.5311	130.0499	65.5286			112.0393	56.5233	E							13
2	**215.1390**	108.0731	**243.1339**	122.0706			225.1234	113.0653	I	1271.5862	636.2968	1254.5597	627.7835	1253.5757	627.2915	12
3	328.2231	164.6152	356.2180	178.6126			338.2074	169.6074	L	***1158.5022***	579.7547	1141.4756	571.2414	1140.4916	570.7494	11
4	**397.2445**	199.1259	425.2395	213.1234			407.2289	204.1181	S	1045.4181	523.2127	1028.3916	514.6994	1027.4075	514.2074	10
5	**512.2715**	256.6394	**540.2664**	270.6368			522.2558	261.6316	D	976.3966	488.7020	959.3701	480.1887	958.3861	479.6967	9
6	611.3399	306.1736	639.3348	320.1710			621.3243	311.1658	V	***861.3697***	431.1885	844.3432	422.6752	843.3591	422.1832	8
7	726.3668	363.6871	754.3618	377.6845			736.3512	368.6792	D	***762.3013***	381.6543	745.2747	373.1410	744.2907	372.6490	7
8	797.4040	399.2056	**825.3989**	413.2031			807.3883	404.1978	A	***647.2743***	324.1408	630.2478	315.6275	629.2638	315.1355	6
9	912.4309	456.7191	940.4258	470.7165			922.4153	461.7113	D	***576.2372***	288.6223	559.2107	280.1090	558.2267	279.6170	5
10	1026.4738	513.7406	1054.4687	527.7380	1037.4422	519.2247	1036.4582	518.7327	N	***461.2103***	231.1088	444.1837	222.5955	443.1997	222.1035	4
11	1141.5008	571.2540	1169.4957	585.2515	1152.4691	576.7382	1151.4851	576.2462	D	***347.1674***	174.0873	330.1408	165.5740	329.1568	165.0820	3
12	1198.5222	599.7648	1226.5172	613.7622	1209.4906	605.2489	1208.5066	604.7569	G	***232.1404***	116.5738	215.1139	108.0606			2
13									R	175.1190	88.0631	158.0924	79.5498			1
