# Supplementary figures and images for: A Role for Barley Calcium-Dependent Protein Kinase CPK2a in the Response to Drought
Source: Front Plant Sci. 2016 Oct 25;7:1550. doi: 10.3389/fpls.2016.01550 (PMC5078816; doi:10.3389/fpls.2016.01550)

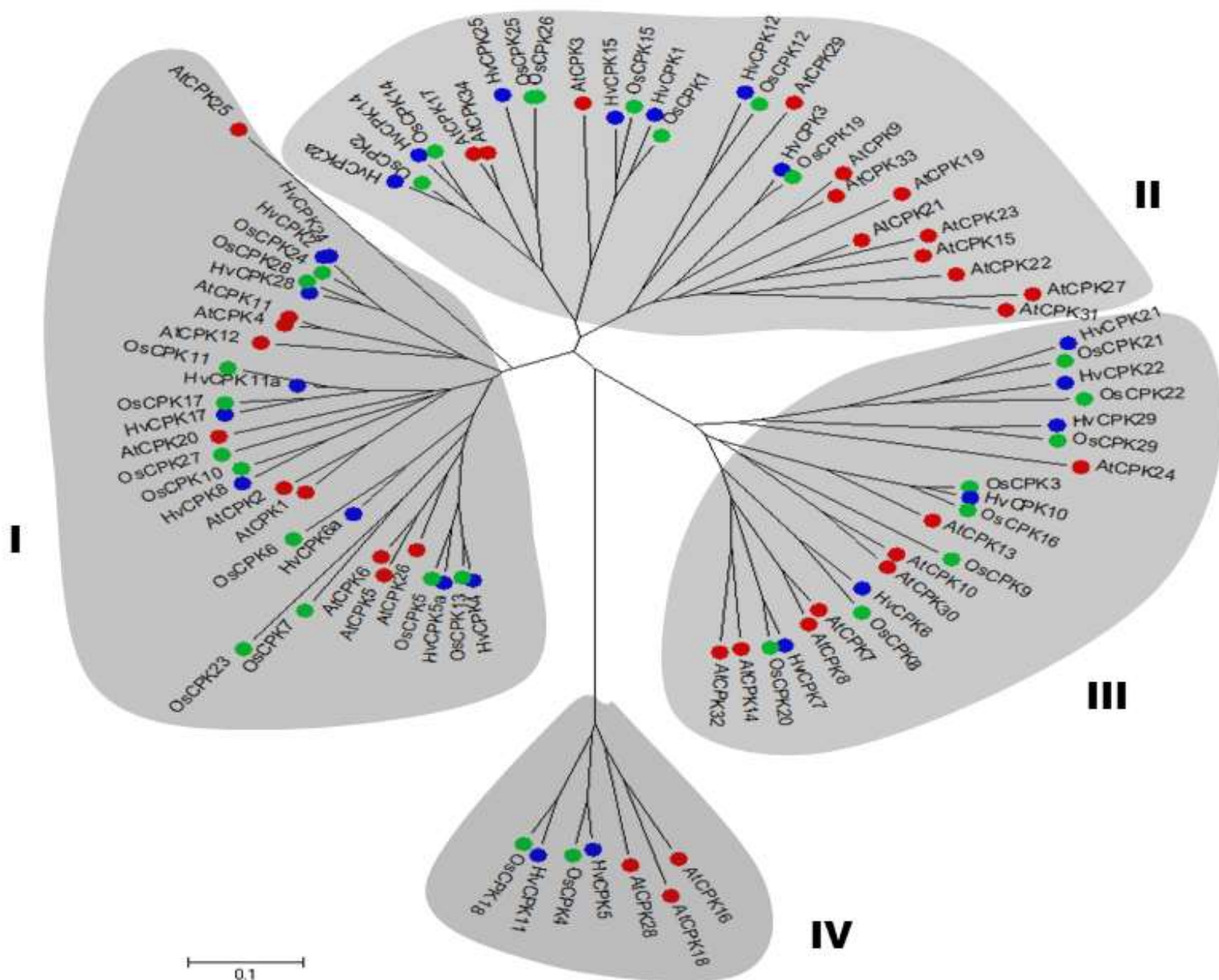

Supplement: Supplementary Figure S1 — Phylogenetic relationship of selected plant calcium-dependent protein kinases. A neighbor-joining phylogenetic tree with 1000 bootstrap replications was generated with MEGA6 software using the full-length ORFs of 34 Arabidopsis (shown in red), 24 rice (green), and 24 barley (blue) CPK isoforms. Amino acid sequences were obtained from NCBI and aligned using Clustal W. [file Image1.PDF]
