# Supplementary material for: A Role for Barley Calcium-Dependent Protein Kinase CPK2a in the Response to Drought
Source: Front Plant Sci. 2016 Oct 25;7:1550. doi: 10.3389/fpls.2016.01550 (PMC5078816; doi:10.3389/fpls.2016.01550)
Supplement: Supplementary Figure S2 — To identify drought-induced CPKs, we used custom-prepared antibodies (AS1 and AS2) against the conserved kinase domain. Antibodies were used to immunoprecipitate protein complexes containing CPKs from drought-treated barley. CPK enzyme activities associated with immune complexes were identified using an in vitro kinase assay. Myelin basic protein (MBP) was used as substrate. The AS1 immune complex revealed strong kinase activity in the drought-treated sample, while the AS2 immune complex showed no kinase activity under either control and drought conditions. CBB staining confirmed equal loading of MBP. [file Image2.PDF]

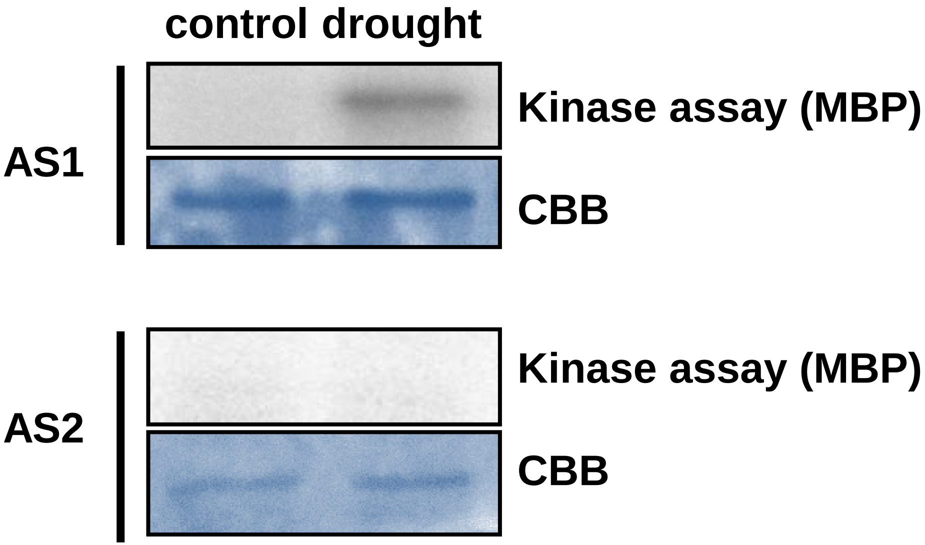

Supplemental Figure 2. To identify drought-induced CPKs, we used custom-prepared antibodies (AS1 and AS2) against the conserved kinase domain. Antibodies were used to immunoprecipitate protein complexes containing CPKs from drought-treated barley. CPK enzyme activities associated with immune complexes were identified using an *in vitro* kinase assay. Myelin basic protein (MBP) was used as substrate. The AS1 immune complex revealed strong kinase activity in the drought-treated sample, while the AS2 immune complex showed no kinase activity under either control and drought conditions. Coomassie staining confirmed equal loading of MBP.
