# Supplementary material for: A Role for Barley Calcium-Dependent Protein Kinase CPK2a in the Response to Drought
Source: Front Plant Sci. 2016 Oct 25;7:1550. doi: 10.3389/fpls.2016.01550 (PMC5078816; doi:10.3389/fpls.2016.01550)
Supplement: Supplementary Figure S3 — Subcellular localization of HvCPK2a in Arabidopsis protoplasts. The WT version of the HvCPK2a-GFP fusion protein is associated with the plasma membrane. HvCPK2aK94M-GFP is also present in the nucleus, while the HvCPK2aD189A variant is present in the cytoplasm and on the cytoplasmic side of the plasma membrane. Hoechst H33342 was used as nuclear localization marker. Scale bar: 10 μm. [file Image3.PDF]

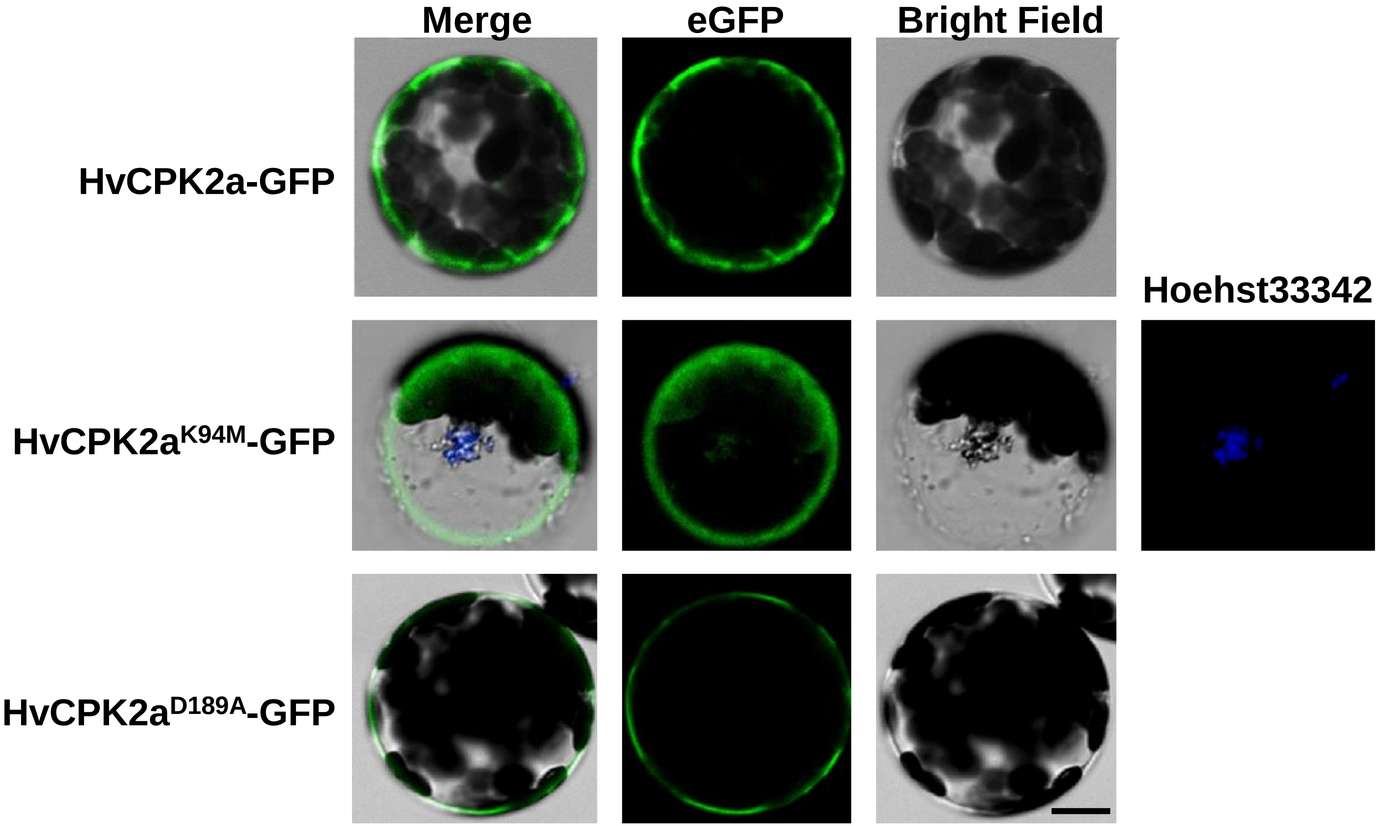

Supplementary Figure S3. Subcellular localization of HvCPK2a in Arabidopsis protoplasts

The WT version of the HvCPK2a-GFP fusion protein is associated with the plasma membrane. HvCPK2a^{K94M}-GFP is also present in the nucleus, while the HvCPK2a^{D189A} variant is present in the cytoplasm and on the cytoplasmic side of the plasma membrane. Hoechst H33342 was used as nuclear localization marker. Scale bar: 10 µm.
